# Ornamenting of Blue Thermally Activated Delayed Fluorescence Emitters by Anchor Groups for the Minimization of Solid-State Solvation and Conformation Disorder Corollaries in Non-Doped and Doped Organic Light-Emitting Diodes

**DOI:** 10.1021/acsami.2c12475

**Published:** 2022-08-24

**Authors:** Malek Mahmoudi, Dalius Gudeika, Stepan Kutsiy, Jurate Simokaitiene, Rita Butkute, Levani Skhirtladze, Kai Lin Woon, Dmytro Volyniuk, Juozas Vidas Grazulevicius

**Affiliations:** † Department of Polymer Chemistry and Technology, Kaunas University of Technology, Radvilenu pl.19, Kaunas LT-50254, Lithuania; ‡ Department of Electronic Devices, Lviv Polytechnic National University, S. Bandera 12, Lviv 79013, Ukraine; § Low Dimensional Material Research Centre, Department of Physics, University Malaya, Kuala Lumpur 50603, Malaysia

**Keywords:** donor, acceptor, trifluoromethyl group, *tert*-butyl carbazole, blue TADF, organic light-emitting diodes

## Abstract

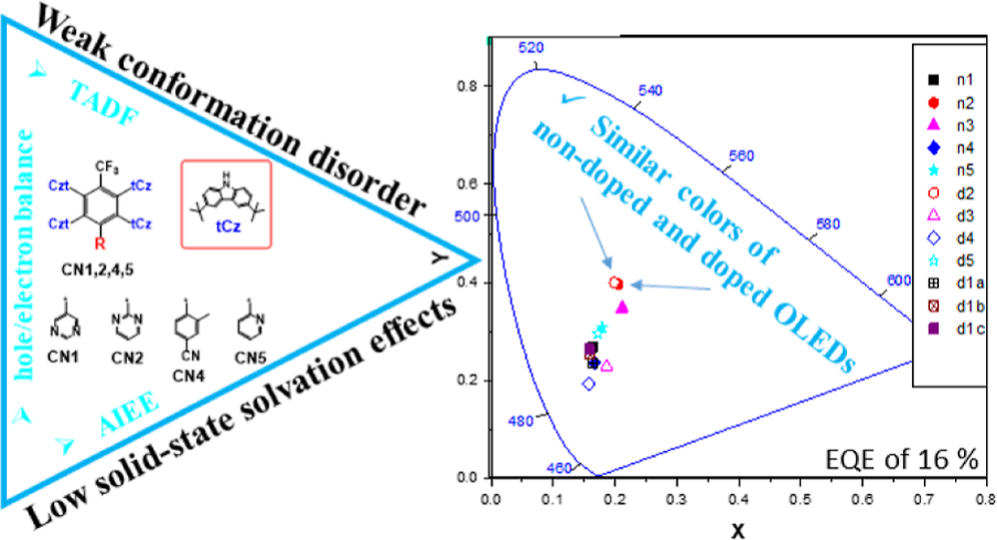

Motivated to minimize the effects of solid-state solvation and conformation disorder on emission properties of donor–acceptor-type emitters, we developed five new asymmetric multiple donor–acceptor type derivatives of *tert*-butyl carbazole and trifluoromethyl benzene exploiting different electron-accepting anchoring groups. Using this design strategy, for a compound containing four di-*tert*-butyl carbazole units as donors as well as 5-methyl pyrimidine and trifluoromethyl acceptor moieties, small singlet-triplet splitting of *ca.* 0.03 eV, reverse intersystem crossing rate of 1 × 10^6^ s^–1^, and high photoluminescence quantum yield of neat film of *ca.* 75% were achieved. This compound was also characterized by the high value of hole and electron mobilities of 8.9 × 10^–4^ and 5.8 × 10^–4^ cm^2^ V^–1^ s^–1^ at an electric field of 4.7 × 10^5^ V/cm, showing relatively good hole/electron balance, respectively. Due to the lowest conformational disorder and solid-state solvation effects, this compound demonstrated very similar emission properties (emission colors) in non-doped and differently doped organic light-emitting diodes (OLEDs). The lowest conformational disorder was observed for the compound with the additional accepting moiety inducing steric hindrance, limiting donor–acceptor dihedral rotational freedom. It can be exploited in the multi-donor–acceptor approach, increasing the efficiency. Using an emitter exhibiting the minimized solid-state solvation and conformation disorder effects, the sky blue OLED with the emitting layer of this compound dispersed in host 1,3-bis(*N*-carbazolyl)benzene displayed an emission peak at 477 nm, high brightness over 39 000 cd/m^2^, and external quantum efficiency up to 15.9% along with a maximum current efficiency of 42.6 cd/A and a maximum power efficiency of 24.1 lm/W.

## 1 Introduction

Organic light-emitting diodes (OLEDs) are promising devices for display and lighting applications thanks to their potentially long operational lifetime and low power consumption.^1,2^ Recently, OLEDs with external quantum efficiencies (EQEs) exceeding 30% have been reported utilizing luminophores with thermally activated delayed fluorescence (TADF).^3,4^ Utilization of TADF materials in OLEDs as the efficient approach to harvest non-emissive triplet excitons without usage of any complicated metal–organic frameworks, requiring relatively inexpensive starting reagents for the synthesis and being sufficiently stable can considerably boost the efficiency of devices, reduce power consumption, and reduce environmental problems in comparison with common prompt fluorescence (PF)-based OLEDs or in comparison with phosphorescent OLEDs.^5^ TADF materials now are considered as the third generation of OLED emitters which allow us to reach internal quantum efficiencies of the devices of 100% without involving noble-metal complexes and incorporation of triplet excitons in radiation which induce the elongated emission lifetimes.^6^ However, regardless of all the efforts dedicated, blue OLEDs remain bottlenecks for the wider application of OLEDs.^7^ To prevent these bottlenecks, the phenomenon of TADF has been studied extensively by the researchers whose interest is related to OLEDs.^8,9^ It received much attention in academic and industrial communities involved in the development of efficient and stable blue OLEDs.^10,11^

Multiple donor–acceptor-type carbazole derivatives as blue TADF emitters showed great potential for the improvement of efficiency and stability of sky-blue OLEDs.^12^ The state-of-art performance (device life-time T90 of *ca.* 600 h at brightness of 1000 cd×m^–2^ and maximum EQE of 29.3%) of sky-blue TADF OLEDs with electroluminescence (EL) spectrum peaking at 486 nm was observed using 9,9′,9″,9‴,9‴′-[6-(4,6-diphenyl-1,3,5-triazine-2-yl)benzene-1,2,3,4,5-pentayl]pentakis(9*H*-carbazole) as blue TADF emitter with extremely fast spin-flipping characterized by reverse intersystem crossing (RISC) rate of 1.5 × 10^7^ s^–1^.^13^ Exploiting strong electron-withdrawing inductive effects of the trifluoromethyl group, in our previous work, we developed asymmetric multi-carbazole-based emitters by utilizing two different types of electron-withdrawing moieties which helped to improve their TADF properties and to achieve efficient blue emission.^14^ These and other examples prove that efficient triplet exciton utilization is possible using multi-channel RISC of multi-donor–acceptor molecules as well as obtaining small singlet-triplet energy splitting (Δ*E*_ST_) resulting from through space and bond charge transfer effects of the molecules.^15^ It could be expected that the alternating arrangement of donor and acceptor units may lead to the coexistence of through-bond charge transfer (TBCT) and through-space charge transfer (TSCT) effects, resulting in a small Δ*E*_ST_ and high photoluminescence quantum yield (PLQY). On the other hand, the multi-(donor/acceptor) structures of the designed compounds promotes spin-vibronic mixing among the multiple excited states, which is indispensable to the efficient multi-channel RISC process.^16^ The carbazole unit is commonly used as electron donor in the design of functional materials for OLEDs.^15^ Carbazole derivatives show good amorphous film-forming features, high triplet energy, hole-transporting ability, low redox potential, and good thermal stability.^17^ The *tert*-butyl group has been widely used in the design of fluorophores.^5^ The attachment of tert-butyl groups to the carbazole moiety allowed us to reduce the concentration-quenching effect and increase solid-state PLQY and stability of the compounds.^18,19^ Using this approach, derivatives of carbazole and benzonitrile as blue TADF emitters were prepared.^9^ OLEDs based on these emitters showed improved device lifetimes and maximum EQE exceeding 21%.^9^ Variety of conjugated electron-accepting groups such cyanobenzene, triazine, oxadiazole, diphenyl sulfoxide, and so forth were used in the design of TADF emitters.^20,21^ However, TADF emitters containing strong electron-deficient trifluoromethyl group have been rarely reported.^22,23^

Molecular design of TADF compounds is performed in such a way, that their lowest excited singlet and triplet states lie very close energetically due to the spatial separation of molecular orbitals of donating and accepting moieties.^24^ This can lead to the thermally activated upconversion from the latter to the former state through RISC which is actually the inverse transition processes of phosphorescence.^25^ The first demonstration of exploitation of RISC phenomenon in OLEDs was reported by Adachi et al.^8,26^ The emission process of TADF compounds is controlled by intramolecular charge transfer (CT) transitions via triplet excitons.^8^ Hence, to meet the requirements of CT and small ΔE_ST_ values at the same time, the combination of electron-donating and electron-withdrawing moieties through a twisted structure is necessary for the rational molecular design of TADF compounds.^5^

**Figure 1:**
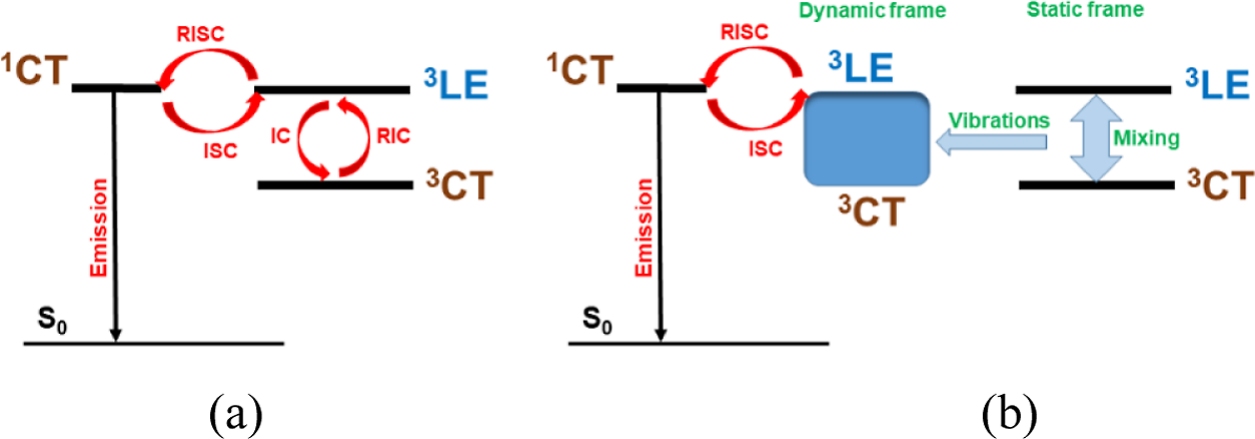
Schematic static (a) and dynamic (b) diagrams of excited states energies of TADF compounds.^27−30^ S_0_ is the ground state, ISC is intersystem crossing, IC is internal conversion, and RIC is reverse internal conversion.

It should be noted that the TADF mechanism is not as simple as it is briefly disclosed above. To better investigate/explain/understand the TADF mechanism, different excited state–energy diagrams were proposed including the “dynamic” one (Figure 1).^27,28^ The main components of those diagrams are not only singlet ^1^CT and triplet ^3^CT states of TADF molecules but also a local excited singlet (^1^LE) and triplet (^3^LE) states of electron-donating and/or electron-accepting species of the molecules (Figure 1).

According to the abovementioned diagrams, RISC/TADF efficiency is to the great extent predominated by energy differences between ^3^CT–^3^LE and ^1^CT–^3^LE (Figure 1). Highly efficient blue TADF can be achieved at equability ^1^CT = ^3^LE = ^3^CT when there are no energy barriers for RISC. Such equability can be achieved by molecular engineering (e.g., by smart molecular design^31^) or physical approaches (e.g., by an appropriate host selection^32^). Notably, ^1^CT and ^3^CT states are very sensitive to media polarity and are different in different surrounding conditions (solvents, hosts different concentrations, conformations, polymorphs, etc.).^33^ Meanwhile, ^1^LE and ^3^LE states are unchangeable under different environments and depend mainly on the chemical structures. As a result, it is easy to lose the “perfect” equability ^1^CT = ^3^LE = ^3^CT for the most efficient TADF materials especially if the CT-type compounds are characterized by very strong solid-state solvation effects and/or conformation disorder.^34,35^ In this work, we aimed to minimize the effects of solid-state solvation and conformation disorder on emission properties of TADF emitters exploiting multi-donor–acceptor molecular engineering.

With this fundamental concern, in this work, we designed and synthesized a series of highly efficient blue TADF emitters based on four 3,6-di-*tert*-butylcarbazole moieties as donors and two electron acceptors. The general electron acceptor for all the synthesized compounds was the trifluoromethyl phenyl group. Our aim in this work was mostly related to the study of the effect of an additional acceptor moiety attached through the para position of the trifluorotoluene moiety on the properties of the emitters. A compound containing four di-*tert*-butyl carbazole units as donors as well as 5-methyl pyrimidine and trifluoromethyl acceptor moieties demonstrated the similar photoluminescence (PL) and electroluminescent (EL) spectra in the layers of non-doped and doped compound and in OLEDs. Thus, the effects of solid-state solvation and conformation disorder of this compound on its TADF properties were considerably decreased which is not typical for TADF compounds.^36^

## 2 Experimental Section

### 2.1 Materials

1-Bromo-2,3,5,6-tetrafluoro-4-(trifluoromethyl)benzene, pyrimidine-5-boronic acid, pyrimidine-2-boronic acid, 2-methyl-4-cyanophenylboronic acid, 2-pyridineboronic acid, and cesium carbonate (Cs_2_CO_3_) were purchased from Sigma-Aldrich. 3,6-Di-*tert*-butyl-9*H*-carbazole (**tCz**) were synthesized according to the previously reported procedure.^16^

#### 2.1.1 General Procedure for Pd-Catalyzed Suzuki–Miyaura Cross-Coupling Reactions of 1-Bromo-2,3,5,6-tetrafluoro-4-(trifluoromethyl)benzene with Aryl Boronic Acids

The compounds of 1-bromo-2,3,5,6-tetrafluoro-4-(trifluoromethyl)benzene (1 equiv), aryl boronic acids (1.5 equiv.), and K_2_CO_3_ (2 M) were dissolved in toluene and stirred at room temperature, to which Pd(PPh_3_)_4_ (10 mol %) was added under nitrogen atmosphere protection. Then, the reaction mixture was refluxed at 90 °C for 16 h under a nitrogen atmosphere. After the mixture was cooled down, water was added to the resulting solution and the mixture was extracted with dichloromethane three times. The organic phase was dried over anhydrous magnesium sulfate and concentrated in vacuum. The dry crude products were used for the further reactions without purification.

5-[2,3,5,6-Tetrafluoro-4-(trifluoromethyl)phenyl]pyrimidine (CF1). Light yellow solid, yield (0.75 g, 76%). MS (APCI^+^, 20 V), *m*/*z*: 297 ([M + H]^+^).

2-[2,3,5,6-Tetrafluoro-4-(trifluoromethyl)phenyl]pyrimidine (CF2). Light yellow solid, yield (0.61 g, 61%). MS (APCI^+^, 20 V), *m*/*z*: 297 ([M + H]^+^).

2′,3′,5′,6′-Tetrafluoro-2-methyl-4′-(trifluoromethyl)-[1,1′-biphenyl]-4-carbonitrile (CF3). Light yellow solid, yield (0.81 g, 73%). MS (APCI^+^, 20 V), *m*/*z*: 334 ([M + H]^+^).

2-[2,3,5,6-Tetrafluoro-4-(trifluoromethyl)phenyl]pyridine (CF4). Light yellow solid, yield (0.68 g, 69%). MS (APCI^+^, 20 V), *m*/*z*: 296 ([M + H]^+^).

#### 2.1.2 General Procedure for Pd-Free Reaction of 1,2,4,5-Tetrafluoro-3-(trifluoromethyl)benzene with 3,6-Di-*tert*-butyl-9*H*-carbazole

9,9′,9″,9‴-[3-(Pyrimidin-5-yl)-6-(trifluoromethyl)benzene-1,2,4,5-tetrayl]tetrakis(3,6-di-*tert*-butyl-9*H*-carbazole) (CN1)*.* To a solution of 3,6-di-*tert*-butyl-9*H*-carbazole (1.27. g, 4.55 mmol) in dimethylformamide (DMF) (10 ml) under an argon atmosphere was added Cs_2_CO_3_ (1.32 g, 4.05 mmol) at 80 °C, and the mixture was stirred for 5 min. 5-[2,3,5,6-Tetrafluoro-4-(trifluoromethyl)phenyl]pyrimidine (**CF1**) (0.3 g, 1.01 mmol) was added, and the mixture was then stirred for 24 h at 120 °C. When the reaction was completed, it was quenched with water. The crude product was extracted with ethylacetate, excess solvent was removed under reduced pressure. The crude product was purified by column chromatography using ethylacetate/*n*-hexane (1:7) as an eluent to give compound **CN1** as a light yellow solid. Yield: 0.56 g, 42%. ^1^H NMR (400 MHz, CDCl_3_, δ, ppm): 8.29 (d, *J* = 8.6 Hz, 2H), 7.60 (d, *J* = 1.7 Hz, 3H), 7.47 (d, *J* = 1.7 Hz, 3H), 7.28 (d, *J* = 8.6 Hz, 2H), 7.22–7.18 (m, 3H), 7.15 (dd, *J*_1_ = 8.6 Hz, *J*_2_ = 1.7 Hz, 4H), 7.07–6.99 (m, 7H), 6.80 (d, *J* = 8.5 Hz, 3H), 1.38 (s, 36H), 1.32 (s, 36H). ^13^C NMR (101 MHz, CDCl_3_, δ, ppm): 157.7, 154.6, 143.0, 139.6, 139.4, 139.0, 138.0, 129.0, 128.2, 127.8, 125.3, 123.8, 123.3, 122.6, 115.6, 115.5, 109.9, 109.1, 34.4, 31.8. MS (APCI^+^, 20 V), *m*/*z*: 1334 ([M + H]^+^). Elemental analysis calcd (%) for C_91_H_99_F_3_N_6_: C, 81.94; H, 7.48; F, 4.27; N, 6.30. Found: C, 81.99; H, 7.52; N, 6.34.

9,9′,9″,9‴-[3-(Pyrimidin-2-yl)-6-(trifluoromethyl)benzene-1,2,4,5-tetrayl]tetrakis(3,6-di-*tert*-butyl-9*H*-carbazole) (CN2) was synthesized using the same procedure as **CN1** but using CF2 (0.3 g, 1.01 mmol) to replace CF1. The crude product was purified by column chromatography using ethylacetate/*n*-hexane (1:6) as an eluent to give compound **CN2** as a light yellow solid. Yield: 0.79 g, 59%. ^1^H NMR (400 MHz, CDCl_3_, δ, ppm): 7.90 (d, *J* = 4.9 Hz, 2H), 7.58 (d, *J* = 1.6 Hz, 3H), 7.41 (d, *J* = 1.6 Hz, 3H), 7.31–7.28 (m, 3H), 7.22–7.18 (m, 3H), 7.14–7.10 (m, 7H), 7.05–7.00 (m, 6H), 1.40 (s, 36H), 1.34 (s, 36H). ^13^C NMR (101 MHz, CDCl_3_, δ, ppm): 155.7, 149.2, 142.4, 142.1, 140.8, 140.8, 140.8, 129.0, 128.2, 125.3, 123.5, 123.1, 122.5, 122.3, 115.2, 114.5, 110.8, 109.9, 34.4, 31.9. MS (APCI^+^, 20 V), *m*/*z*: 1334 ([M + H]^+^). Elemental analysis calcd (%) for C_91_H_99_F_3_N_6_: C, 81.94; H, 7.48; F, 4.27; N, 6.30. Found: C, 81.97; H, 7.43; N, 6.33.

9,9′,9″,9‴-[3-Bromo-6-(trifluoromethyl)benzene-1,2,4,5-tetrayl]tetrakis(3,6-di-*tert*-butyl-9*H*-carbazole) (CN3) was synthesized using the same procedure as **CN1** but using 1-bromo-2,3,5,6-tetrafluoro-4-(trifluoromethyl)benzene (0.3 g, 1.01 mmol). The crude product was purified by column chromatography using ethylacetate/*n*-hexane (1:8) as an eluent to give compound **CN3** as a light yellow solid. Yield: 0.83 g, 62%. ^1^H NMR (400 MHz, CDCl_3_, δ, ppm): 7.62 (d, *J* = 1.8 Hz, 6H), 7.60 (d, *J* = 1.8 Hz, 2H), 7.14 (dd, *J*_1_ = 8.6 Hz, *J*_2_ = 1.8 Hz, 2H), 7.12 (d, *J* = 1.8 Hz, 4H), 7.11–7.08 (m, 3H), 7.08–7.01 (m, 5H), 6.90 (d, *J* = 8.6 Hz, 2H), 1.40 (s, 36H), 1.37 (s, 36H). ^13^C NMR (101 MHz, CDCl_3_, δ, ppm): 143.0, 139.1, 138.2, 137.1, 123.9, 123.2, 122.6, 122.4, 115.4, 109.7, 109.0, 34.5, 32.8. MS (APCI^+^, 20 V), *m*/*z*: 1334 ([M + H]^+^). Elemental analysis calcd (%) for C_87_H_96_BrF_3_N_4_: C, 78.29; H, 7.25; Br, 5.99; F, 4.27; N, 4.20. Found: C, 78.33; H, 7.30; N, 4.24.

2′,3′,5′,6′-Tetrakis(3,6-di-*tert*-butyl-9*H*-carbazol-9-yl)-2-methyl-4′-(trifluoromethyl)-[1,1′-biphenyl]-4-carbonitrile (CN4) was synthesized using the same procedure as **CN1** but using **CF1** was replaced by **CF3** (0.3 g, 0.90 mmol). The crude product was purified by column chromatography using ethylacetate/*n*-hexane (1:7) as an eluent to give compound **CN4** as a light yellow solid. Yield: 0.62 g, 51%. ^1^H NMR (400 MHz, CDCl_3_, δ, ppm): 7.68 (d, *J* = 1.6 Hz, 2H), 7.63–7.55 (m, 3H), 7.51 (dd, *J*_1_ = 5.9 Hz, *J*_2_ = 1.6 Hz, 3H), 7.44 (dd, *J*_1_ = 7.3 Hz, *J*_2_ = 5.9 Hz, 3H), 7.36 (t, *J* = 5.9 Hz, 2H), 7.21 (d, *J* = 7.3 Hz, 2H), 7.13–7.02 (m, 2H), 6.78–6.64 (m, 5H), 6.58–6.44 (m, 5H), 2.12 (s, 3H), 1.45 (s, 36H), 1.26 (s, 36H). ^13^C NMR (101 MHz, CDCl_3_, δ, ppm): 143.2, 142.7, 139.2, 138.5, 137.3, 132.5, 129.9, 128.4, 127.1, 123.7, 123.4, 123.2, 122.5, 121.8, 115.6, 115.1, 110.3, 109.4, 34.3, 31.9, 21.3. MS (APCI^+^, 20 V), *m*/*z*: 1372 ([M + H]^+^). Elemental analysis calcd (%) for C_95_H_102_F_3_N_5_: C, 83.23; H, 7.50; F, 4.16; N, 5.11. Found: C, 83.27; H, 7.54; N, 5.17.

9,9′,9″,9‴-[3-(Pyridin-2-yl)-6-(trifluoromethyl)benzene-1,2,4,5-tetrayl]tetrakis(3,6-di-*tert*-butyl-9*H*-carbazole) (CN5) was synthesized using the same procedure as **CN1** but using CF4 (0.3 g, 1.02 mmol). The crude product was purified by column chromatography using ethylacetate/*n*-hexane (1:9) as an eluent to give compound **CN5** as a light yellow solid. Yield: 0.93 g, 69%. ^1^H NMR (400 MHz, CDCl_3_, δ, ppm): 7.74 (d, *J* = 4.2 Hz, 1H), 7.59 (d, *J* = 1.6 Hz, 4H), 7.42 (d, *J* = 1.6 Hz, 4H), 7.14 (dd, *J*_1_ = 4.2 Hz, *J*_2_ = 1.6 Hz, 4H), 7.10–6.92 (m, 13H), 6.77–6.71 (m, 1H), 6.43–6.34 (m, 1H), 1.39 (s, 36H), 1.33 (s, 36H). ^13^C NMR (101 MHz, CDCl_3_, δ, ppm): 148.2, 142.5, 142.2, 139.6, 138.7, 138.2, 134.7, 123.6, 123.1, 122.7, 122.5, 122.3, 122.1, 115.2, 114.7, 110.2, 110.1, 34.4, 31.9. MS (APCI^+^, 20 V), *m*/*z*: 1333 ([M + H]^+^). Elemental analysis calcd (%) for C_92_H_100_F_3_N_6_: C, 82.91; H, 7.56; F, 4.28; N, 5.25. Found: C, 82.88; H, 7.54; N, 5.21.

## 3 Results and Discussion

### 3.1 Synthesis and Characterization

**Scheme 1:**
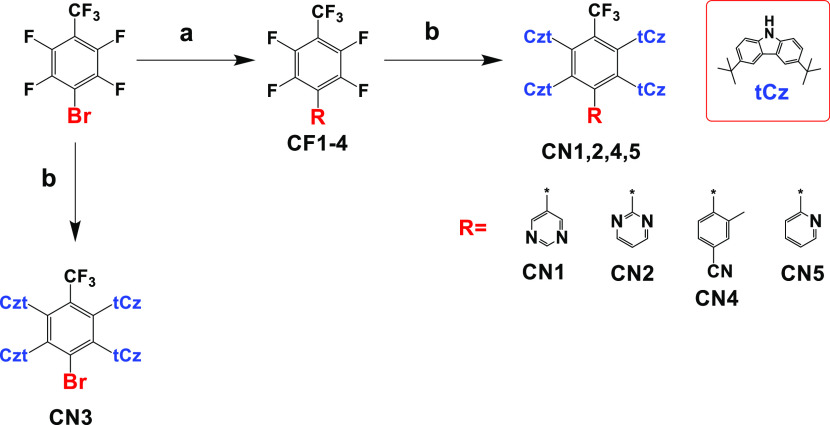
Synthesis of **CN1–CN5** Reagents and conditions: (a) aryl boronic acids (pyrimidine-5-boronic acid, pyrimidine-2-boronic acid, 2-methyl-4-cyanophenylboronic acid, 2-pyridineboronic acid), K_2_CO_3_, Pd(PPh_3_)_,_ toluene, 90 °C, 16 h; (b) Cs_2_CO_3_, DMF, 120 °C, 24 h

Compounds **CN1–CN5** were prepared by catalyst-free aromatic nucleophilic substitution reactions of bromo-2,3,5,6-tetrafluoro-4-(trifluoromethyl)benzene, 5-[2,3,5,6-Tetrafluoro-4-(trifluoromethyl)phenyl]pyrimidine (CF1), 2-[2,3,5,6-tetrafluoro-4-(trifluoromethyl)phenyl]pyrimidine (CF2), 2′,3′,5′,6′-tetrafluoro-2-methyl-4′-(trifluoromethyl)-[1,1′-biphenyl]-4-carbonitrile (CF3), and 2-[2,3,5,6-tetrafluoro-4-(trifluoromethyl)phenyl]pyridine (CF4) with 3,6-di-*tert*-butyl-9*H*-carbazole (Scheme 1, see also Supporting Information). 3,6-Di-*tert*-butyl-9*H*-carbazole was prepared according to the procedure reported in the literature.^16^ Intermediate compounds **CF1-4** were synthesized by Pd-catalyzed Suzuki cross-coupling reactions of 1-bromo-2,3,5,6-tetrafluoro-4-(trifluoromethyl)benzene with aryl boronic acids, that is, pyrimidine-5-boronic acid, pyrimidine-2-boronic acid, 2-methyl-4-cyanophenylboronic acid, and 2-pyridineboronic acid. All the compounds were obtained with relative good yields. The target derivatives were fully characterized by ^1^H NMR, ^13^C NMR spectroscopies, elemental analysis, and mass spectrometry. Synthetic procedures and characterization data for **CN1–CN5** can be found in the Supporting Information.

### 3.2 Frontier Orbitals

**Figure 2:**
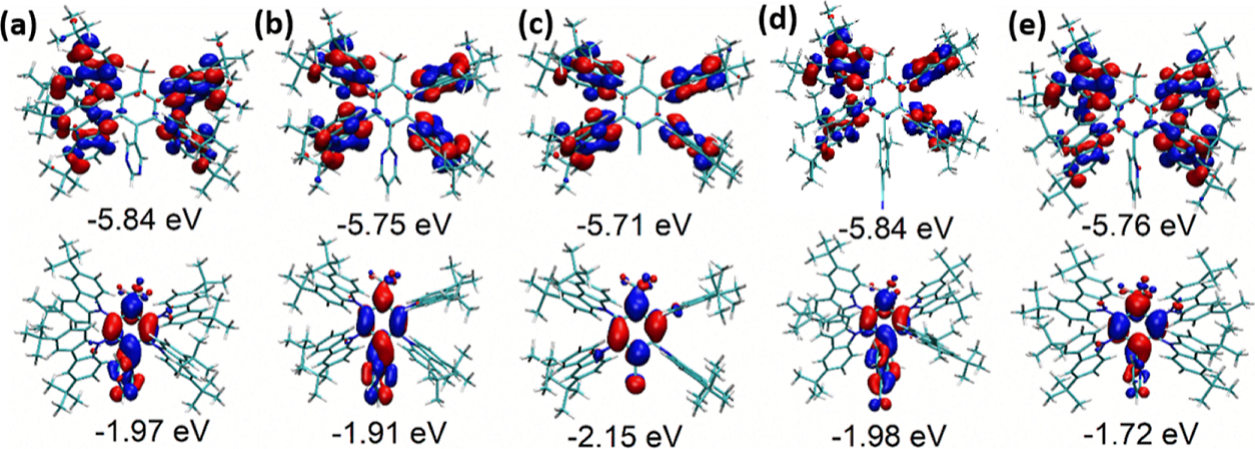
HOMO (top) and LUMO (bottom) along with their energy levels of (a) **CN1**, (b) **CN2**, (c) **CN3**, (d) **CN4**, and (e) **CN5**.

Density functional theory (DFT) using range-separated hybrid functional LC-ωPBEh was used to obtain the ground state molecular geometry at the def2-svp basis set. ω was tuned using the golden ratio algorithm under the polarizable continuum model (PCM) with a dielectric constant of 2.38 corresponding to toluene and a solvent radius of 3.48 Å. The optimum ω was determined to be of 0.0144 for **CN1–CN5**. Their optimized structures are shown in Figure 2. The **CN1–CN5** exhibited large dihedral angles of 72.7, 71.6, 72.2, 71.7, and 69.6° between 3,6-di-*tert*-butyl-9H-carbazoles which are located next to trifluoromethyl and the acceptor moieties, respectively. The dihedral angles between donor moieties close to **R** and the acceptors are of 51.8, 69.2, 69.2, 73.6, and 70.3°, respectively. The smaller dihedral angle in **CN1** is due to steric hindrance from the proximity of the hydrogen atoms at 5-[4-(trifluoromethyl)phenyl]pyrimidine. The shortest distance between the hydrogen atom at 4, 6 positions to the next nearest hydrogen atom in the donor is of 2.98 Å for **CN1**, while in **CN2**, the smallest distance between the nitrogen atoms to the hydrogen atom in the donor is of 3.85 Å. This results in the re-position of the donors next to sterically hindered acceptor moiety in **CN1**. Cyanobenzene, pyrimidine, and pyridine as **R** in Scheme 1 are additional electron acceptor moieties. Their dihedral angles with (trifluoromethyl)benzene moieties are of 64.8, 58.6, 68.9, and 70.5° for **CN1**, **CN2**, **CN4**, and **CN5**, respectively. The large dihedral angle should disrupt the conjugation. However, in this case, it behaves similar to a single acceptor with least unoccupied molecular orbital (LUMO) extending into the trifluoromethyl groups due to hyperconjugation (Figure 2). **CN3** has the deepest LUMO compared with the other compounds studied, indicating that Br is the strongest electron accepting substituent compared with cyanobenzene, pyrimidine, and pyridine. As expected, pyridine is the weakest electron acceptor among the groups as it has only one nitrogen atom and hence **CN5** has the shallowest LUMO level. The highest occupied molecular orbital (HOMO) levels are approximately the same for **CN1** to **CN5** with a variation between −5.71 and −5.84 eV as the HOMO level is dominated by the 3,6-di-*tert*-butyl-9H-carbazole moiety.

Vertical excited states were obtained using time-dependent-DFT/LC-ωPBEh//def2-svp at optimal ω under the same PCM model. The singlet and triplet transitions for **CN1–CN5** exhibited the charge-transfer character dominated by HOMO to LUMO transition. An excited state wavefunction is represented as the linear combination of the single-determinant configurations with associated coefficients. The coefficients are of 0.986, 0.986, 0.840, 0.972, and 0.992 for singlet transition from HOMO to LUMO in **CN1–CN5**, respectively. For **CN3**, singlet transitions are also contributed by HOMO → LUMO+1 with the associated coefficient of 0.503. The vertical excitation energies for **CN1–CN5** are of 2.83, 2.84, 2.91, 2.91, and 2.98 eV with singlet and triplet gaps of 0.03, 0.04, 0.04, 0.06, and 0.08 eV which are very small.

### 3.3 Electrochemical and Thermal Properties

**Figure 3:**
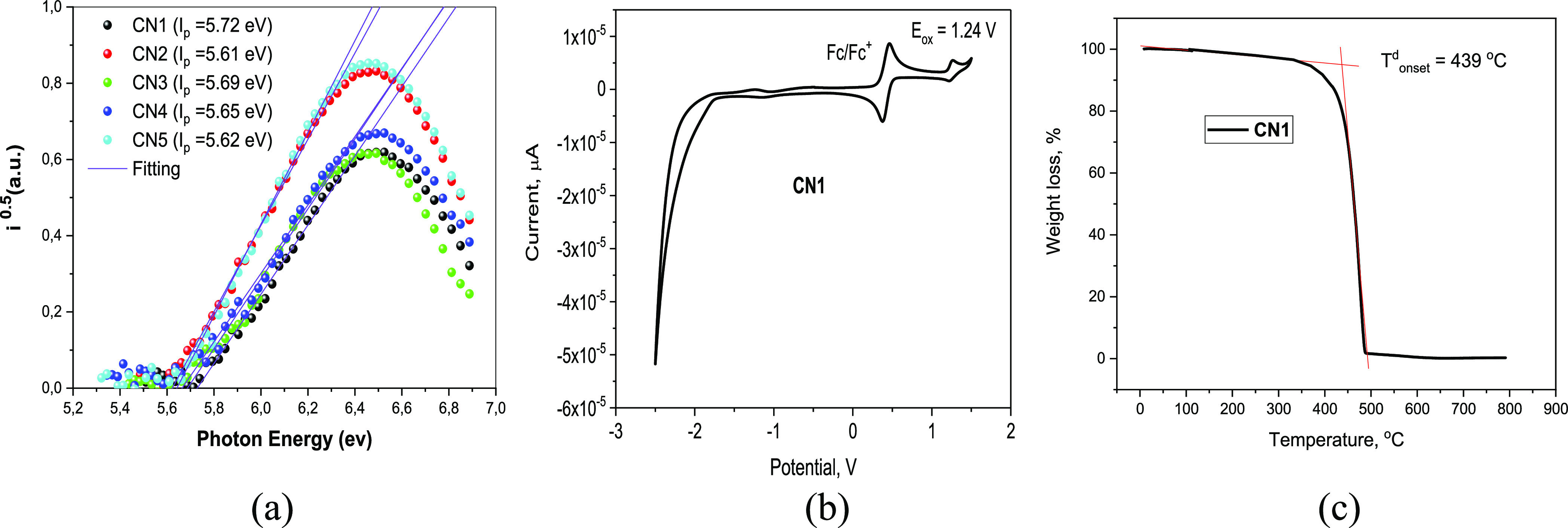
Photoelectron emission spectra of solid films of the compounds recorded in air (a), CV curve of dilute solutions of **CN1** in dichloromethane (100 mV/s) (b) and TGA curve of **CN1** (c).

The ionization potential (IP_CV_) and electron affinity (EA_CV_) values of **CN1–CN5** were estimated by cyclic voltammetry (CV) measurements. As shown in Figures 3 and S2, the similar single quasi-reversible oxidations were observed for the compounds corresponding to the oxidation of the di-*tert*-butyl carbazolyl moiety in the anodic scans. The onset oxidation potentials were found to be of 1.24 V for **CN1,** 1.31 V for **CN2,** 1.27 V for **CN3,** 1.20 V for **CN4,** and 1.23 V for **CN5**. Taking the results of the onset potentials versus the Fc/Fc^+^ of the oxidation curves, the IP_CV_ values were calculated to be in an extremely close range of 5.65–5.76 eV. This small variation is consistent with the DFT calculation. EA_CV_ values were determined according to the equation EA_CV_ = −(|IP_CV_| – *E*_g_) using the optical band gap energies taken from absorption spectra of the compounds. Photoelectron emission spectroscopy was employed to investigate the ionization energy (IP_PE_) of the solid samples of the compounds (Figure 3a and Table 1). The IP_PE_ values were obtained from the intersection points of the linearly extrapolated low binding energy sides of the spectra with the horizontal axis.^37^ The IP_PE_ values of the studied compounds were found to be in the close range of 5.61 to 5.72 eV.

**Table 1:** Oxidation Potential versus the Fc/Fc^+^, Ionization Potentials, Electron Affinities, and Optical Bad Gaps of **CN1**–CN**5**

derivative	*E*onsetox *vs* Fc,^a^ V	IP_CV_^a^, eV	*E*_g_^b^, eV	EA_CV_^c^, eV	IP_PE_^d^, eV	*E*_g_^e^, eV	EA_PE_^f^, eV
**CN1**	0.83	5.76	2.9	2.86	5.72	2.87	2.85
**CN2**	0.78	5.69	2.93	2.74	5.61	2.98	2.63
**CN3**	0.82	5.75	2.95	2.8	5.69	2.97	2.72
**CN4**	0.78	5.69	2.93	2.76	5.65	2.97	2.68
**CN5**	0.75	5.65	2.99	2.66	5.62	2.96	2.66

a Estimated by CV of solutions in CH_2_Cl_2_.^38^

b Taken from the absorption spectra of the dilute THF solutions.

c EA_CV_ = IP_CV_ – *E*_g_.

d Estimated by photoelectron emission spectrometry of solid films.

e Taken from the absorption onset of solid films.

f EA_PE_ = IP_PE_ – *E*_g_.

The temperatures of thermal transitions of compounds **CN1, CN2, CN3, CN4**, and **CN5** were measured by thermogravimetric analysis (TGA) and differential scanning calorimetry. The data obtained are given in Table 2. During the TGA experiments, **CN1–CN5** compounds exhibited complete weight losses, indicating sublimation. Their temperatures of the onsets of weight loss ranged from 433 to 462 °C (Supporting Information, see Figure S1). All the derivatives (**CN1–CN5)** did not show evident signals of glass transitions or meltings within the entire range from −40 to 425 °C.

**Table 2:**
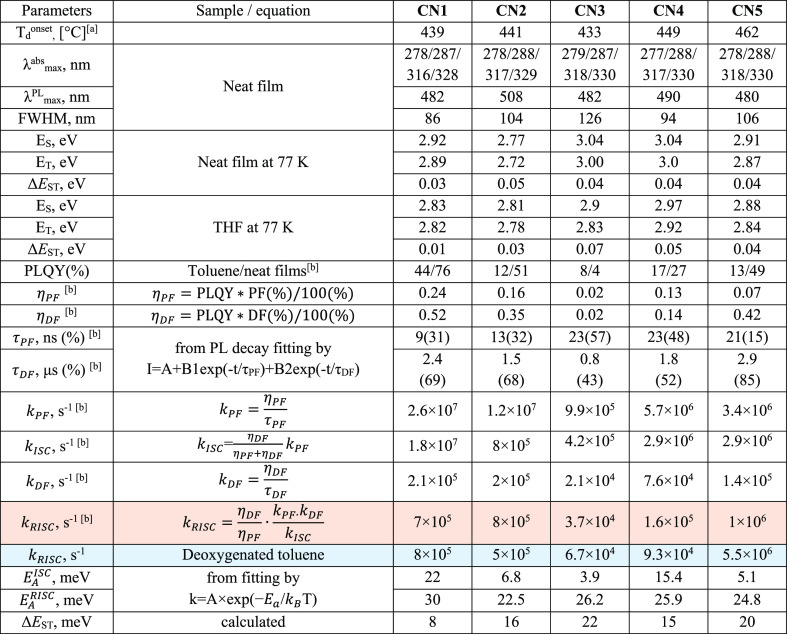
Photophysical and Thermal Properties of **CN1–CN5**

a *T*_d_^onset^ is the temperature of onset of weight loss (20 °C/min, nitrogen atmosphere).

b Neat films.

### 3.4 Photophysical Properties

**Figure 4:**
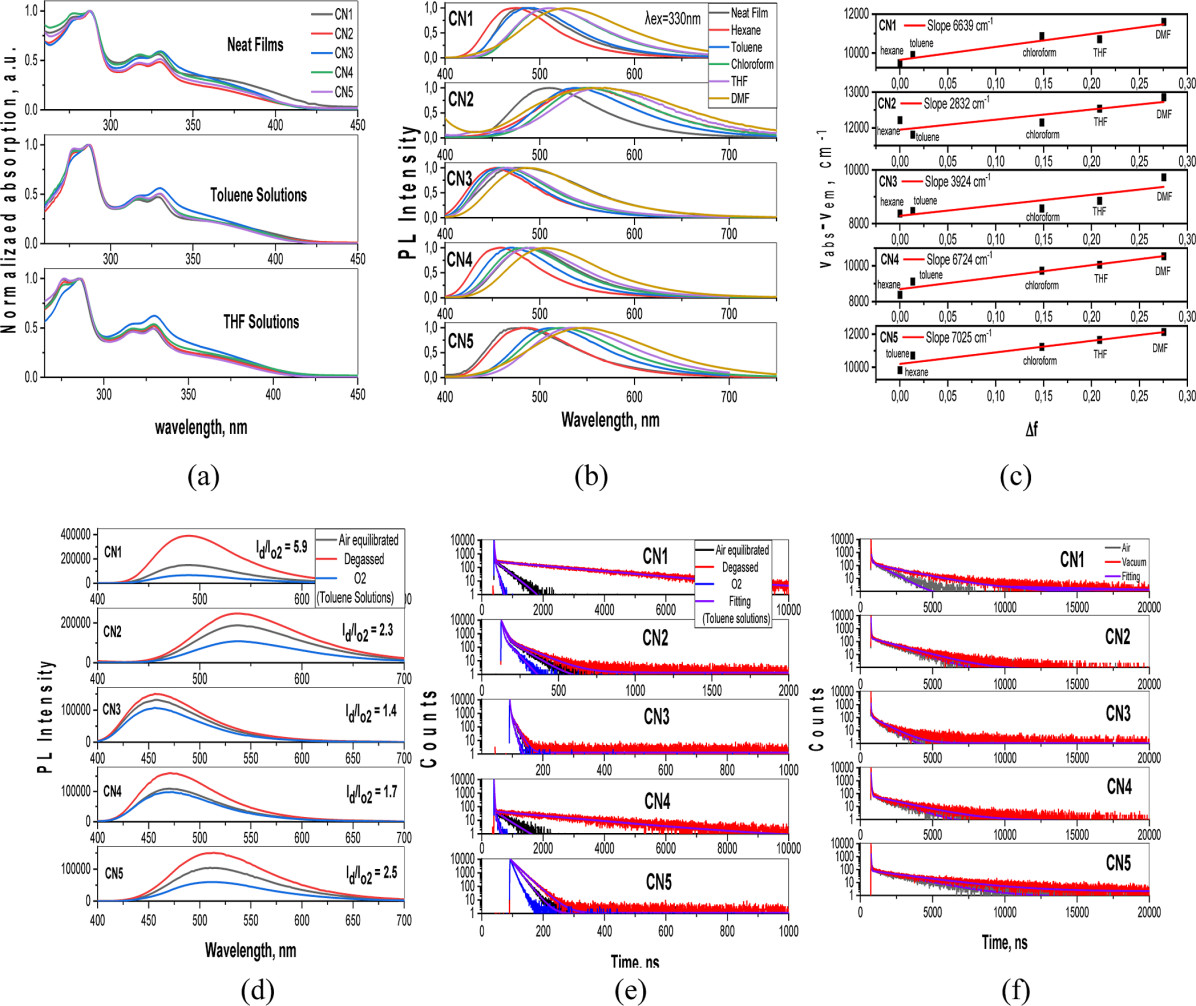
UV–vis (a) and PL (b) spectra of dilute solutions (concentration of 10^–5^ M) and films of compounds **CN1–CN5**, Lippert–Mataga plot of the compounds (Δν = ν_abs_ – ν_em_ is Stokes shift and Δ*f* is orientation polarizability of solvents). (c) PL spectra (d) and PL decay curves (e,f) of air-equilibrated, degassed, and oxygenated toluene solution of compounds (d,e) and films (f) of compounds **CN1–CN5** under air and vacuum. Excitation wavelength λ_ex_ = 330 nm.

UV–vis absorption and PL spectra of the dilute solutions and neat films of the compounds are presented in Figure 4a,b. The selected spectral data are summarized in Table 2. The similar absorption profiles were recorded for the toluene and THF solutions as well as neat films of the compounds. All the synthesized emitters showed broad absorption bands in the range of 340–425 nm, which apparently originate from intramolecular charge transfer transition between *tert*-butyl carbazole donors and trifluoromethyl phenyl and/or additional acceptor. In addition, all the synthesized compounds demonstrated absorption band in the high-energy region, that is, in the range of 276–287 nm attributed to the overlapping of π–π* transitions of electron-withdrawing and -donating units and the absorption bands observed in the range of 316–330 nm which can be ascribed to *n*–π* transitions of di-*tert*-butylcarbazole moieties of compounds **CN1–CN5**.^39^ Non-structured PL spectra of neat films of compounds **CN1–CN5** were observed with the peaks at 482, 508, 482, 490, and 480 nm, respectively (Figure 4b). In order to investigate the solvatochromic behavior of the compounds, PL spectra of the solutions in five different solvents were recorded. Such measurements allow us to obtain information on the emission nature of the compounds. CT emission of TADF compounds is highly sensitive to solvent polarity.^24^ Featureless emission spectra with a single broad band of the conventional TADF emitters must is red-shifted and broadened with the increase in solvent polarity due to the CT character of the first singlet excited state.^40,41^ No obvious shifts of absorption spectra, but considerable red shifts of the fluorescence spectra on going from non-polar hexane (dielectric constant ε = 1.88, Δ*f* = 0.0001) to highly polar DMF (ε = 36.7, Δ*f* = 0.2755) were observed. This observation indicates that the dipole moment changes were contributed by the excited states. The largest Stokes shift between steady state absorption and fluorescence spectra in the series was observed for **CN5** (Figure 4b). PL spectra of this compound with the maxima at 488 and 550 nm were recorded for the solutions in low-polarity hexane and highly polar DMF. Thus, the shift of 68 nm was recorded. Smaller red shifts of 50, 14, 30, and 50 were observed for PL spectra of the solutions of **CN1–CN4**. The plot of Stokes shift [Δ(ν)] versus orientation polarizability (Δ*f*) described by the Lippert–Mataga equation is displayed in Figure 4c.^42^ The linear dependence with a slope of 7025 cm^–1^ was obtained using linear fitting of the Lippert–Mataga plot showing the difference of dipole moments (μ) of CN5 in the ground and excited states. However, in general, the studied compounds were characterized by relatively small slopes of their Lippert–Mataga dependences. This observation can be explained by the weak CT character of PL of these compounds probably with not pure CT or LE emission nature. It should be noted that conventional TADF emitters are characterized by much higher slopes of their Lippert–Mataga dependences than 10 000 cm^–1^.^43,44^ This result additionally indicates that the emission of compounds **CN1–CN5** is less sensitive to media polarity in comparison to that of conventional TADF emitters.

Using the integrate sphere, PLQY values of the solutions of the synthesized compounds in toluene and of the solid samples under ambient and oxygen-free conditions were measured. The data are summarized in Table 2. The solid-state PLQY of compound **CN1** in oxygen-free conditions exceeded 75%, which is excellent value for OLED applications. The PLQY values of the degassed solutions of **CN1–CN5** in toluene were found to be of 44, 12.4, 7.5, 16.7, and 13.4%, respectively. These results indicate the phenomenon of aggregation-induced emission enhancement (AIEE). To support the statement concerning AIEE, we provided PL measurements for the dispersions of compounds **CN1–CN5** in the mixtures of THF and water (see “AIEE” section in the Supporting Information). Figure 4d shows PL spectra of air-equilibrated, degassed, and oxygenated toluene solutions of the compounds. The significant drop-off of intensities of the emission measured for oxygenated toluene solutions compared to those observed after deoxygenation with argon is obvious for all compounds. The oxygen sensitivity and emission enhancement after deoxygenation reveal the involvement of triplet states in emission. The PL intensity of the deoxygenated toluene solution of **CN1** was found to be by *ca.* 6 times higher than that of the oxygenated toluene solution (Figure 4d). We also conducted the PL decay measurements of the toluene solutions of the compounds at room temperature. As it is shown in Figure 4e, delayed emission of oxygenated toluene solutions of the compounds was effectively quenched by oxygen and could no longer be detected. Unlike oxygenated samples, degassed solutions of all the emitters exhibited double exponential decays containing both PF and delayed fluorescence (DF) components. The lifetimes of DF and PF (τ_DF_ and τ_PF_) estimated for air-equilibrated and degassed toluene solutions were obtained by fitting the transients with double exponential decay profiles (Figure 4e, Tables S1 and S4). The significant longer fluorescence lifetimes were found for the toluene solution of compounds **CN1** and **CN4**. They showed longer lifetimes of delayed components in comparison with other compounds. The PL decay curve of degassed solution of **CN1** displays a prompt emission with the lifetime (τ_PF_) of 15 ns together with delayed emission with the lifetime (τ_DF_) of 2.1 μs. The percentage of PF is 19.9% and that of DF is 80.1%. After exposition of the solution to air, the delayed component became negligible (τ_DF_ = 56 ns), demonstrating that the delayed emission of **CN1** increased from the triplet states. The determined lifetimes as well as PLQYs and DF/PF intensity ratios were further used to calculate the RISC rate (*k*_RISC_) according to the previously reported method assuming that non-radiative decay occurs mainly from the triplet states.^45,46^ The recorded *k*_RISC_ values of the studied compounds were found to be in the range from 6.7 × 10^4^ to 5.5 × 10^6^ s^–1^. Compounds **CN1** and **CN5** exhibited blue TADF with fast spin-flip and very high RISC rates of 8 × 10^5^ and 5.5×10^6^ s^–1^, respectively. The fast RISC process results in the reduction of the possibility of the degradation mechanisms in which triplet excitons are involved. The main degradation pathway of TADF OLEDs occurs owing to the instability of the TADF emitters and unwanted photophysical parameters such as a small RISC rate (*k*_RISC_) and a long DF lifetime (τ_d_).^47,48^ Steady-state fluorescence spectra and PL decay curves of the films of compounds **CN1–CN5** recorded at room temperature are shown in Figures 4f and S3. The data are collected in Table S2. Upon removing air, the PL intensities increased, while shapes of the spectra remained unchanged. This observation is another proof of the contribution of triplet excited states in whole PL spectra of the compounds. Figure S3 shows PL spectra of the films of **CN1–CN5** recorded in air and in vacuum. The highest increase of emission intensity after evacuation was recorded for the solid layer of compound **CN1** (*I*_vacuum_/*I*_air_ = 1.6). PL decay curves of the neat films recorded under air and in vacuum were biexponential, manifesting the combination of PF and DF components. PL decay curves of all the compounds showed an increase in the delayed component under the vacuum condition (Figure 4f). Taking into account that no phosphorescence was observed at room temperature (only at low temperatures) and the PL spectra of the delayed and prompt emissions were similar, the delayed component can mostly be assigned to the TADF mechanism. It is noteworthy that the DF lifetime of the neat film of **CN3** under vacuum is as short as 0.77 μs and there is very small difference between the contribution of DF in PL decay curves recorded under air and in vacuum conditions [τ_DF_ under air = 0.61 μs (42%), τ_DF_ under vacuum = 0.77 μs (43%)]. Taking into account that **CN3** was the only compound which had a bromine atom instead of additional conjugated acceptor, this observation can prove the considerable impact of additional conjugated acceptor moieties on delayed emission properties. Meanwhile, the results of PL decay measurements of neat films of other compounds under air and vacuum demonstrated increase of the shares of long-lived components after removal of oxygen. For example, high share delayed emission with a lifetime (τ_d_) of 2.9 μs was recorded for **CN5** neat film under vacuum. The contribution of DF in the PL decay curves of this compound increased from 65.3 to 85.16% after evacuation (Figure 4d and Table S2). The calculations were also performed to determine the rate constants and efficiencies of the key photophysical transitions of the films of **CN1–CN5** (Table 2). At 300K, the rates of intersystem crossing (*k*_ISC_) ranged between 4.2 × 10^5^ and 1.8 × 10^7^ s^–1^. The rates of RISC (*k*_RISC_) ranged between 3.7 × 10^4^ and 1 × 10^6^ s^–1^ which are favorable for the highly efficient up-conversion process of TADF.

**Figure 5:**
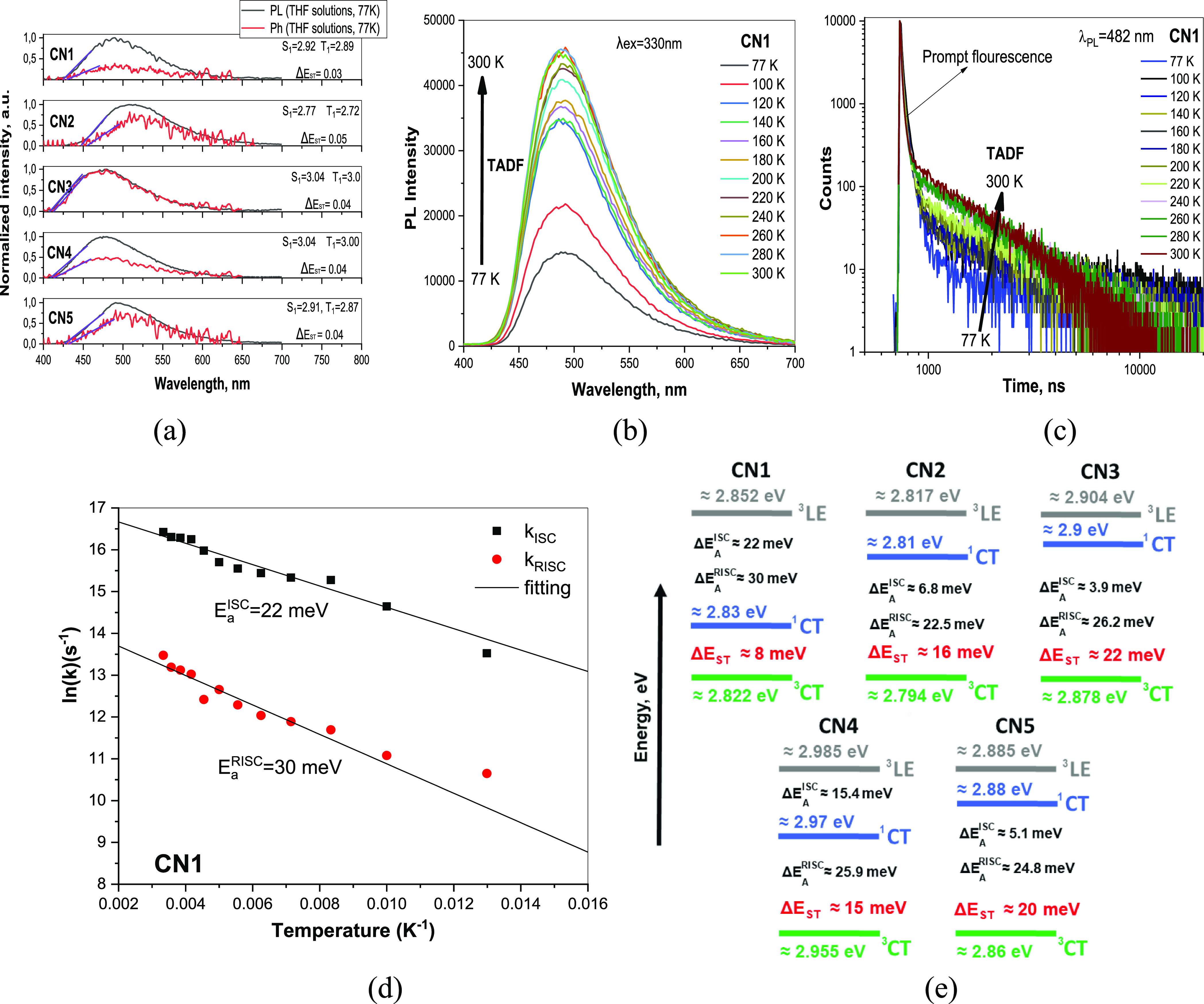
Fluorescence and phosphorescence spectra of the THF solutions of **CN1** recorded at 77 K (a), PL spectra (b), PL decay curves (c), temperature dependences of *k*_ISC_ and *k*_RISC_ (d) for the film of **CN1**, and energy diagram (e) of compounds **CN1–CN5.**

Using the temperature dependences of rate constants *k*_ISC_ and *k*_RISC_ (Figures 5d and S13, see also “Temperature dependent steady state and time resoled PL measurements” section in the Supporting Information), the ISC and RISC activation energies (*E*_A_^ISC^ and *E*_A_^RISC^) were calculated from the slopes of the plots (Table 2). The Arrhenius dependence *k* = *A* × exp(−*E*_a_/*k*_B_T) was used for the fitting in which *E*_a_ is the activation energy, *k*_B_ is the Boltzmann constant, and *A* is the frequency factor involving the spin–orbit coupling constant.^49^ The close *E*_A_^RISC^ values ranging from 22.5 to 30 meV were obtained for **CN1–CN5.** Meanwhile, the values of *E*_A_^ISC^ were found to be quite different. The highest *E*_A_^ISC^ of 22 eV was obtained for compound **CN1**. As a result, the smallest Δ*E*_ST_ of 8 meV was obtained for this compound as it is depicted in the energy diagram (Figure 5e). The smallest *E*_A_^ISC^ of 3.9 eV and the highest Δ*E*_ST_ of 20 meV were obtained for compound **CN3** suggesting its relatively poor TADF properties. The similar trends of experimental and calculated Δ*E*_ST_ values were obtained for **CN1–CN5** (Table 2). The presented energy diagram was constructed according to the procedure described in ref (34). ^1^CT levels were experimentally estimated. Taking into account that the intersystem and RISC are uphill processes in case of **CN1–CN5**, the ^3^LE, ^3^CT, and Δ*E*_ST_ energies were calculated by the questions ^3^LE = ^1^CT + *E*_A_^ISC^, ^3^CT = ^3^LE – *E*_A_^RISC^, and Δ*E*_ST_ = ^1^CT-^3^CT using the corresponding activation energies. The different energy maps of ^1^CT, ^3^LE, and ^3^CT of **CN1–CN5** are mainly responsible for their different TADF efficiencies.

To further investigate the photophysical properties of compounds **CN1–CN5**, PL and phosphorescence spectra of their THF solutions (10^–5^ M) were recorded at 77 K (Figure 5a). From the onsets of PL and phosphorescence spectra of the solutions of the emitters in THF, the Δ*E*_ST_ values of **CN1–CN5** were estimated to be 0.03, 0.05, 0.04, 0.04, and 0.04 eV. This observation confirms that in this type of multi-(donor/acceptor) molecules frontier orbitals can be separated which bring about small ΔE_ST_ (Table 2).

To gain more insights into the DF, temperature-dependent steady-state and time-resolved emission measurements were performed for neat film of compound **CN1**. Figure 5b shows PL spectra of the films of **CN1** recorded at the different temperatures. The intensity of DF increased with the increase of the temperature between 77 and 300 K. This observation confirms thermal activation of the delayed emission. As shown in Figure 5c, PL decay curves of the film of recorded at the different temperatures exhibit two clear components of PF in the nanosecond scale and DF in the microsecond region. The share of long-lived (DF) component was obviously temperature dependent on the temperature. The ratio of the delayed to prompt components slowly grew when the temperature was increased, demonstrating the occurrence of a thermally activated process. Following the temperature increase, the ratio of DF showed significant enhancement trend up to 51% at 300 K, proving the distinct TADF properties of **CN1** (Figure 5b). The small singlet-triplet splitting, very high PLQY, and fast RISC rate (*k*_RISC_) of **CN1** suggest that is a good candidate as a highly efficient TADF emitter for OLED.

**Figure 6:**
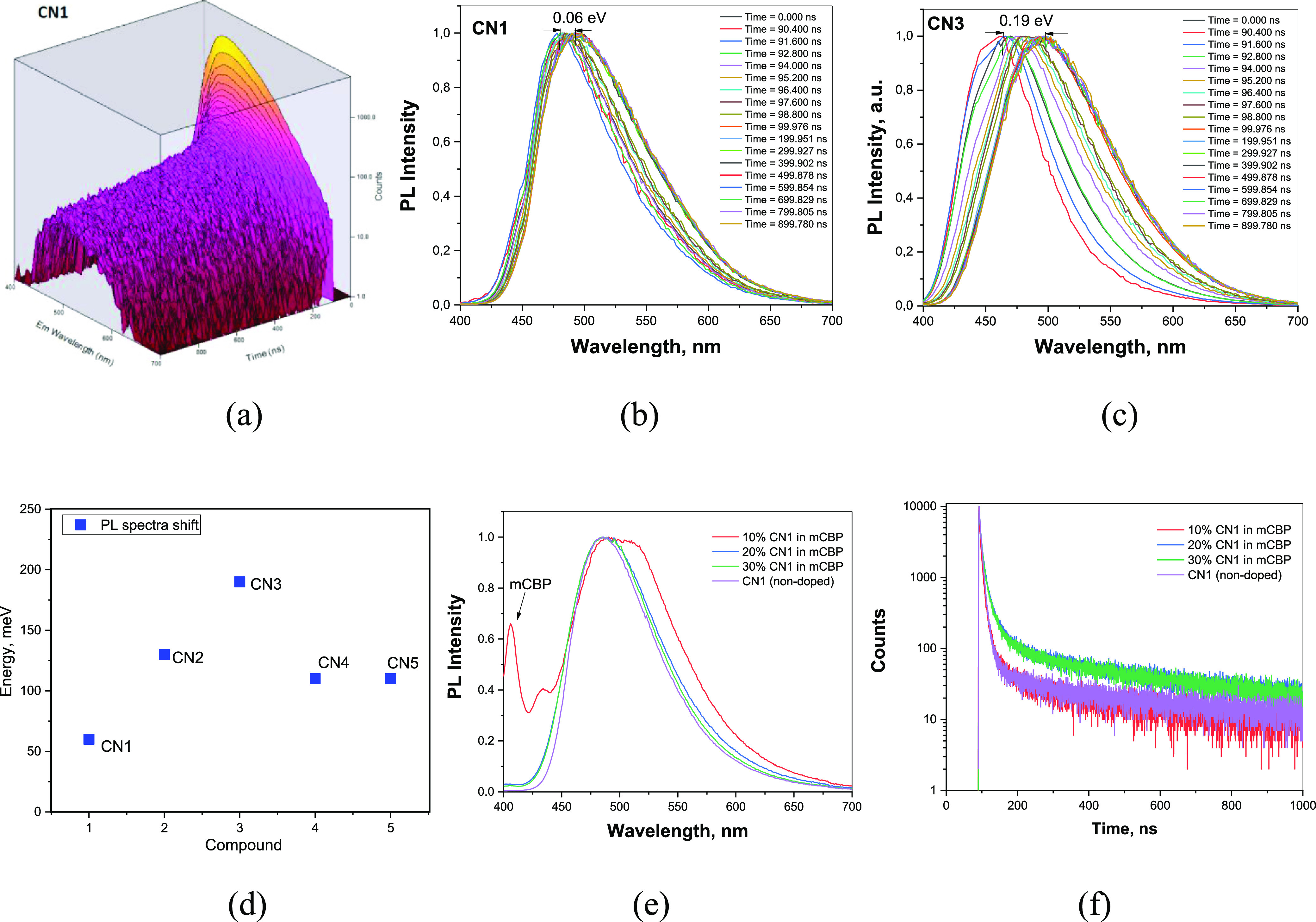
TRES data in 3D (a) and 2D (b,c) plots, the relative PL spectral shifts for the studied compounds (d), PL spectra (e), and PL decays (f) of the films of neat **CN1** and doped in the mCBP host.

We also investigated the conformer formation and hosting effects for **CN1–CN5** on their emission properties (Figure 6). First, time-resolved emission spectra (TRES) were recorded for vacuum-deposited neat films (Figures 6a–c and S4). According to TRES data in 3D (combination of all the spectra recorded in the time range of up to 1 μs) or in 2D (selected spectra recorded at the different delays after excitation), there are shifts of PL spectra which can be attributed to the different conformational disorders of **CN1–CN5**.^34^ The lowest conformational disorder was observed for compound **CN1** for which the lowest shift of PL spectra of 0.06 eV was observed (Figure 6d). The highest conformational disorder was obtained for compound **CN3** containing no additional accepting moiety. This observation well supports the selection of multi-donor–acceptor molecular engineering for the minimization of effects of solid-state solvation and conformation disorder on emission properties of TADF emitters. The similar PL spectra and PL decays were also recorded for the films of neat **CN1** and doped in the mCBP host (Figure 6e,f). Due to the low effect of conformation disorder, the similar EL spectra were also obtained for non-doped and doped devices as it will be discussed in the section “Fabrication and Characterization of OLEDs”. The high **CN3** conformational disorder indicated that the presence of additional accepting moiety increases steric hindrance between the additional accepting moiety with the donor reducing the freedom of torsional disorder between the donor and acceptor. The lower conformational disorder of **CN1** compared to that of **CN2** provided the further clue of the role of steric hindrance in the reduction of conformational disorder. In **CN1**, the hydrogen atoms at 4,6 positions gave rise to the steric repulsive forces against further dihedral distortion. In **CN2**, the hydrogen atoms at 4,6 positions are situated further from the donor moiety. This resulted in reduced steric hindrance.

### 3.5 Charge Transporting Properties

**Figure 7:**
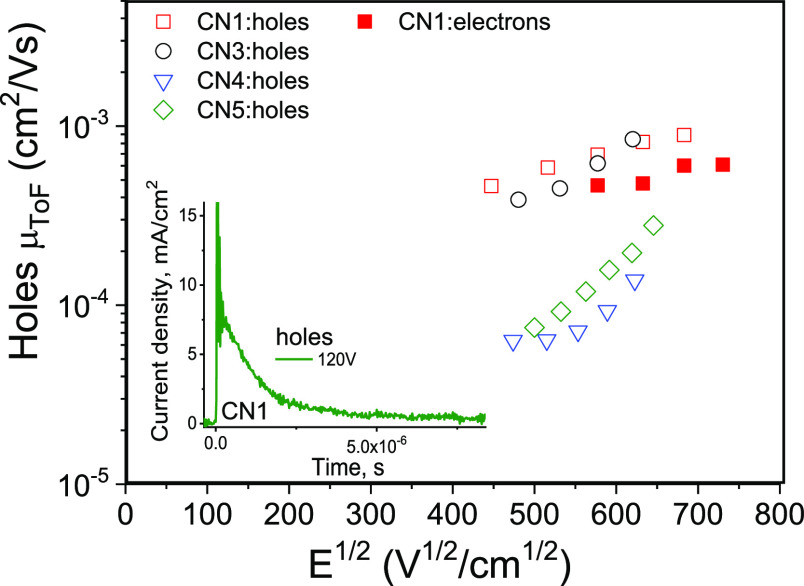
Hole (open symbols) and electron (filled symbols) mobilities of the vacuum-deposited films of **CN1–CN5** as the function of electric field plotted according to the Poole–Frenkel charge mobility dependence [μ = μ_0_ × exp(β × *E*^1/2^).^53^ Inset shows the TOF signal in linear scales for holes in the film of **CN1** recorded at 120 V.

Investigation of charge-transporting properties of potential functional materials for OLED applications is vitally important because holes and electrons have to recombine within light-emitting layers of OLEDs.^50^ In the ideal case, non-doped light-emitting layers of OLEDs are characterized by bipolar/ambipolar charge-transporting properties with well-balanced hole and electron mobilities. Otherwise, the appropriate host has to be selected.^51^ Therefore, to estimate the applicability of compounds **CN1–CN5** as emitters in OLEDs, their charge-transporting properties were studied by the time of flight (TOF) method. The samples with the structure indium tin oxide (ITO)/thick vacuum-deposited layer/Al were fabricated. Having TOF current transients in linear scales (Figure 7, inset), transit time (*t*_tr_) could not be accurately estimated due to the dispersive charge transport. However, for compounds **CN1** and **CN3-5**, *t*_tr_ were well observed in TOF current transients in log–log scales under applied positive voltages to the ITO side, indicating the hole transport (Figure S5). Charge transport in **CN2** was not detected by the TOF experiment apparently because of strong charge recombination. From TOF current transients recorded under the negative voltages, *t*_tr_ values were obtained only for compound **CN1** additionally, indicating its electron-transporting property (Figure S5). Thus, both p-type (hole) and n-type (electron) transport was confirmed for **CN1** by the TOF measurements. Hole and electron mobilities were calculated at the different electric fields using *t*_tr_ values from the corresponding TOF current transients (Figures 7 and S5). **CN1** was characterized by the highest value of hole mobility of 8.9 × 10^–4^ cm^2^ V^–1^ s^–1^ at electric field of 4.7 × 10^5^ V/cm. Its electron mobility of 5.8 × 10^–4^ cm^2^ V^–1^ s^–1^ was not much lower than hole mobility at the same electric field showing relatively good hole/electron balance and suitability for the application in non-doped OLEDs. The values of hole/electron mobilities of **CN1** were among the highest as for TADF emitters.^52^

Compound **CN3** demonstrated similar hole-transporting properties to those of **CN1**. Meanwhile, electron transport was not detected for **CN3**. Apparently the CF_3_ group is not strong enough acceptor to induce the electron-transporting ability for **CN3**. Compounds **CN4** and **CN5** were characterized by lower hole mobilities of 1.4 × 10^–4^ and 2 × 10^–4^ cm^2^ V^–1^ s^–1^, respectively, at an electric field of 3.9 × 10^5^ V/cm apparently because the different HOMO–HOMO overlappings caused by molecular packing properties (Figure 7). As a result, the best EL performances can be expected for **CN1**.

### 3.6 Fabrication and Characterization of OLEDs

**Figure 8:**
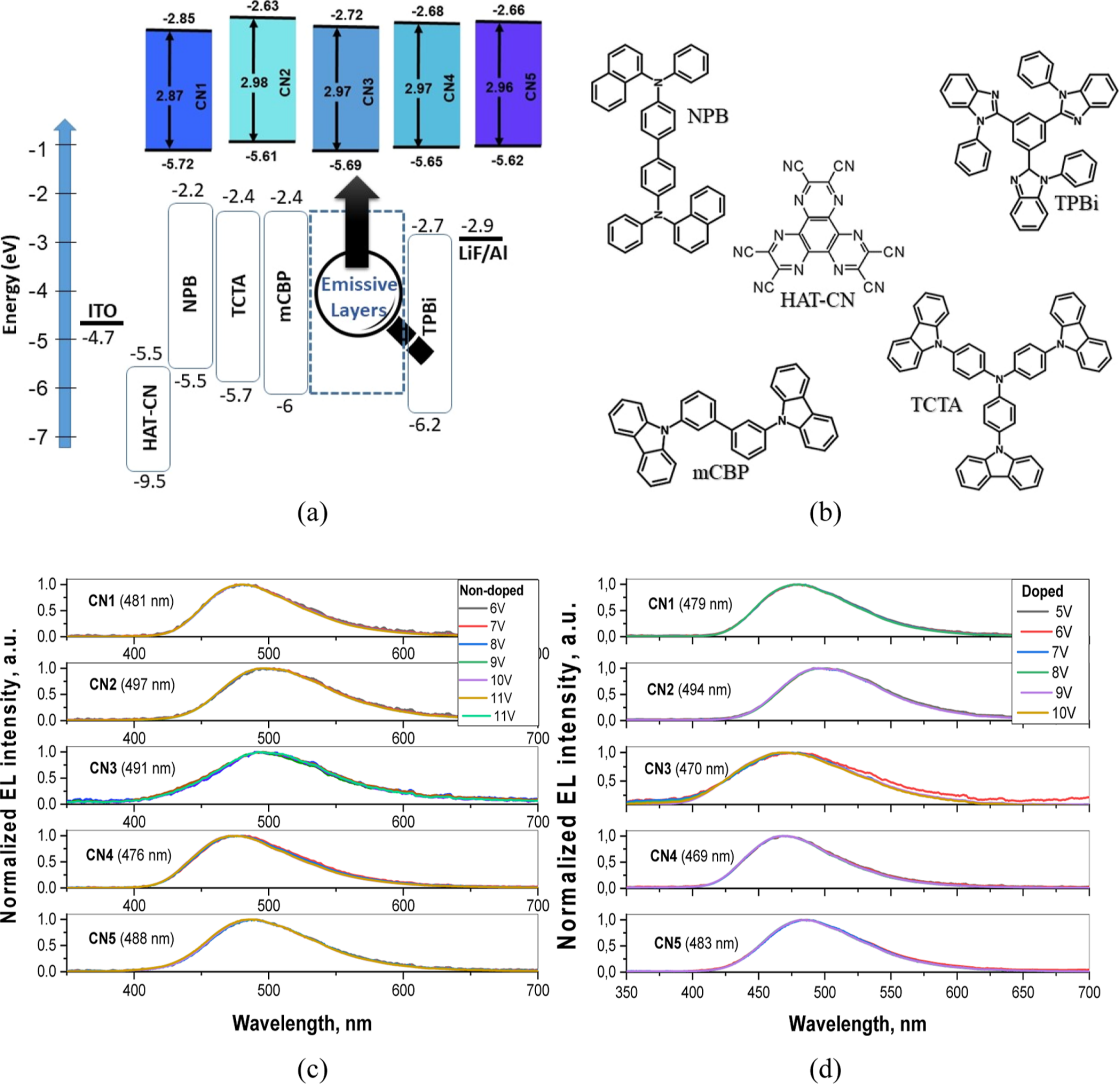
Visualized device structure with indication of energy levels of all functional layers (a) the molecular structures of the compounds used in the devices (b), EL spectra of non-doped (c), and doped devices (d) recorded at different voltages.

**Figure 9:**
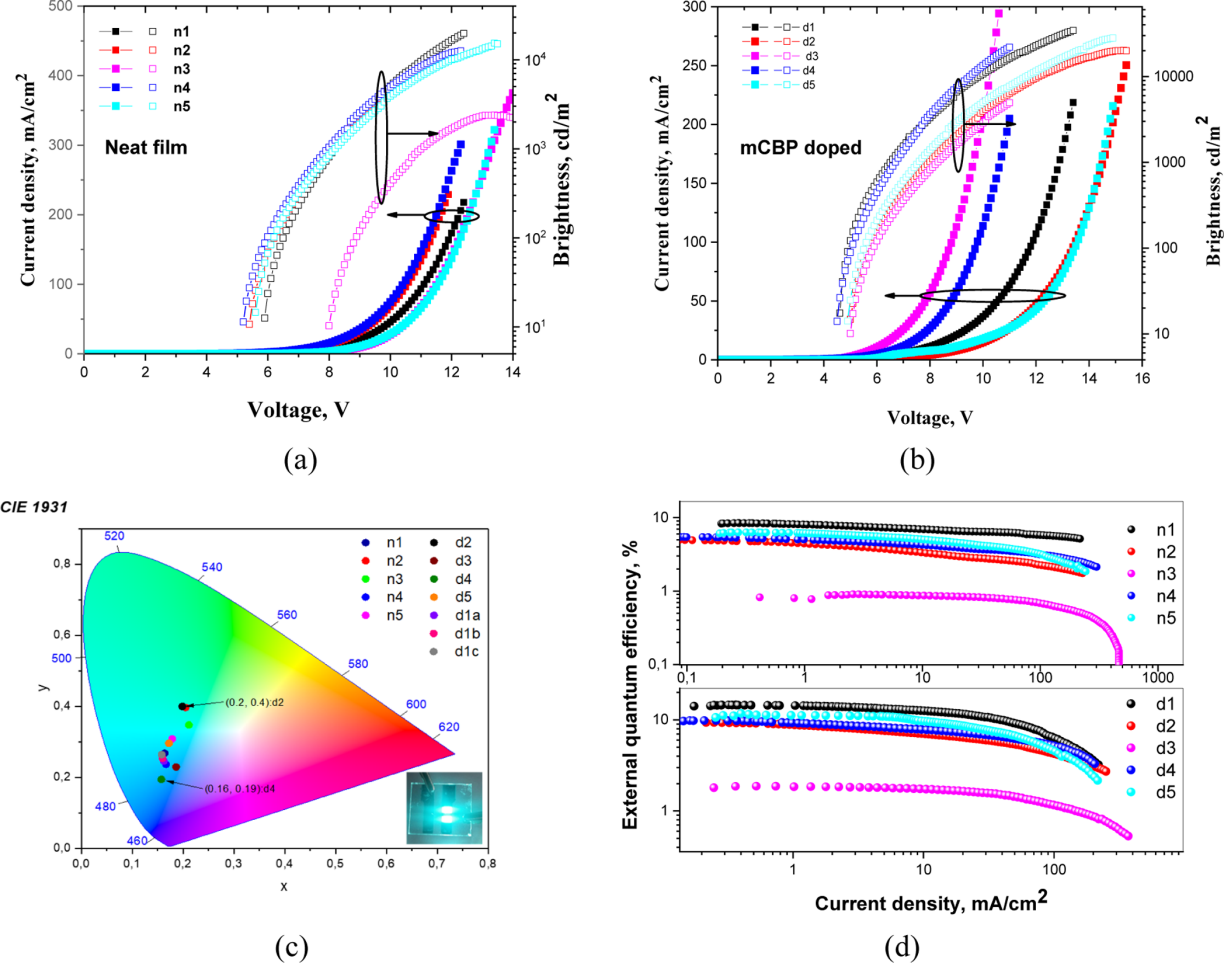
Current density and brightness vs voltage curves (a,b), CIE1931 coordinates (c), and EQE vs current density (d) of the fabricated OLEDs.

Owing to high PLQY values of neat films (exceeding 0.75) and high RISC rates up to 1 × 10^6^ s^–1^, the studied compounds can be regarded as promising blue TADF emitters for OLEDs. EL properties of the compounds were investigated using different device structures based on non-doped and doped emitters in device structures ITO/HAT-CN/NPB/TCTA/mCBP/**CNs**/TPBi/LiF:Al for non-doped and ITO/HAT-CN/NPB/TCTA/mCBP/mCBP: **CNs**/TPBi/LiF:Al for doped devices. The device structures, energy diagram, and chemical structures of organic materials are shown in Figure 8a,b. In this architecture, hexaazatriphenylene hexacarbonitrile (HAT-CN) was functionalized as a hole injection material, *N*,*N*′-di (1-naphthyl)-*N*,*N*′-diphenyl-(1,1′-biphenyl)-4,4′-diamine (NPB) as a hole-transporting material, tris(4-carbazoyl-9-ylphenyl)amine (TCTA) as an electron-blocking material, 3,3′-di(9H-carbazol-9-yl)-1,1′-biphenyl (mCBP) as a host and exciton blocking compound, 2,2′,2″-(1,3,5-benzine-triyl)-tris(1-phenyl-1-H-benzimidazole) (TPBi) as an electron-transporting material, and the layer of lithium fluoride (LiF) as an electron injection layer. Vacuum-deposited doped layers were fabricated by employing 10 wt % doping concentration of **CN1–CN5** emitters in the mCBP host. mCBP was selected as the host material because of its high singlet (3.6 eV) and triplet (2.9 eV) energies.^54^ mCBP is often combined with high band gap, blue-light emitters in the emissive layer of OLEDs.^54^ To evaluate the EL performance of **CN1** more precisely and with the aim of additional optimization of device structure OLEDs with the different concentrations of emitter (10, 20 to 30 wt %) in the host were fabricated. The device characteristics such as current density–voltage–luminance (*J*–*V*–*L*) characteristics, EQE versus current density curves as well as the EL spectra and CIE color coordinates are presented in Figures 8, 9, S7, and S8. The key parameters are summarized in Table 3. Figure 8c,d shows the EL spectra and emission color coordinates of OLEDs containing the layers of non-doped and doped **CN1–CN5**. All the fabricated devices exhibited unstructured bright blue emission with EL peaks (λ_EL_) at 473–497 nm and EL spectra similar to PL spectra in the neat films. Due to the low polarity of the host mCBP and due to the polarity-sensitive CT emission of the synthesized compounds, blue shifts were recorded for the EL spectra of the doped devices d1–d5 compared to the corresponding spectra of non-doped devices n1–n5. In order to evaluate the device stability, EL spectra were recorded at the different voltages. Very stable EL spectra were observed under the different driving voltages (Figure 8c,d). The stable EL spectra indicate the absence of effects of conformation disorder which could be present under the different driving voltages. The CIE coordinates of fabricated devices were also rather stable under various driving voltages. The CIE_*x*_ color coordinates of the fabricated devices were measured to be in the range of 0.16–0.21 and CIE_*y*_ color coordinates were in the range of 0.19–0.4 (Figure 9c and Table 3). Figure 9a,b and Table 3 indicate that relatively low turn-on voltages were observed for both fabricated non-doped and doped devices. This observation confirms efficient injection from electrodes and transport of charge carriers to the emissive layers. Doped devices d1–d5 were characterized by lower turn-on voltages than non-doped ones (n1–n5). This observation can most probably be assigned to good charge-transporting properties of light-emitting layers containing ambipolar host mCBP with relatively high hole and electron mobilities. The turn-on voltages (*V*_on_) from 5.2 to 7.9 V were observed for non-doped devices, while for the doped OLEDs, these values were considerably lower (4.1–5 V).

**Table 3:**
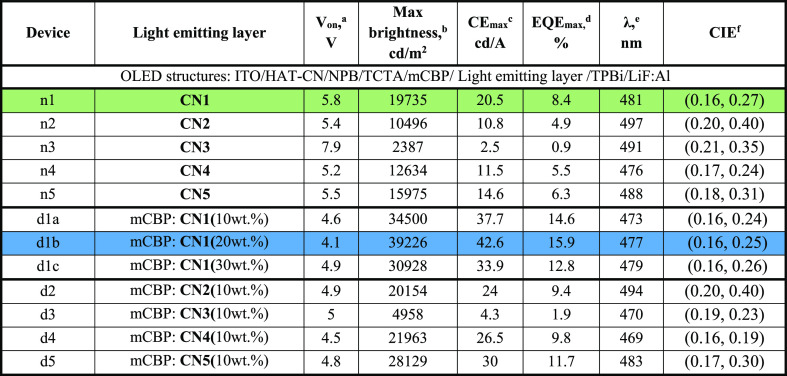
Parameters of OLEDs

a Turn-on voltage at a luminance of 10 cd m^–2^.

b Maximum brightness.

c Maximum current efficiency.

d Maximum EQE.

e Wavelength of the peak of EL spectrum at 6V.

f Commission Internationale de I’Eclairage (CIE) 1931 color coordinates.

Non-doped device n1 based on compound **CN1** showed sky-blue EL peaking at 481 nm with Commission Internationale de L’Eclairage (CIE) coordinates of (0.16, 0.27), maximum brightness (*B*_max_) of 19 735 cd/m^2^, maximum current efficiency (CE_max_) of 20.5 cd/A, maximum power efficiency (PE_max_) of 12.4 lm/W, and EQE_max_ of 8.4%. The optimal doping concentrations of compound **CN1** in mCBP was determined to be 20 wt %. The optimized device d1b with EL peak at 477 nm showed high maximum EQE, current efficiency (CE), and brightness of 15.9%, 42.6 cd/A, and 39 226 cd/m^2^, respectively (Figure S6 and Table 3). With the increased concentration of the emitter in the host of 30 wt %, device d1c showed slightly lower efficiency than d1a and d1b. This decrease of efficiency is apparently related to the concentration quenching and exciton annihilation effects governed by strong intermolecular interactions.^55^ This device exhibited maximum EQE of 12.8%, maximum current efficiency of 33.9 cd/A, and brightness of 30 928 cd/m^2^ with slightly red-shifted EL peak (at 479 nm) as compared to the devices d1a and d1b with lower concentration of emitter in the host. Because the relatively low triplet levels of mCBP (2.9 eV) and TPBi (2.74 eV), the studied OLEDs did not show high EQE values (Table 3).^56^ Considering high triplet levels (2.72–3.0 eV) of the developed emitters **CN1–CN5** (Table 2), further optimization of our device structures should be related to the replacement of used host mCBP and electron-transporting layer TPBi by appropriate materials with high triplet levels (at least on 0.2 eV higher triplet levels than that of **CN1–CN5**). To demonstrate how much the experimental EQE values are lower than the corresponding theoretical EQEs, we performed the additional analysis according to the formula η_ext_ = γ × Φ_PL_ × χ × η_out_,^57^ where γ corresponds to the charge-balance factor, Φ_PL_ is the PL quantum efficiency, χ is the efficiency of exciton production, and η_out_ corresponds to the outcoupling efficiency. The theoretical analysis of EQE values was carried out taking γ and χ as 1 which are typical values for TADF compounds. η_out_ is typically in the range from 0.2 to 0.3 for glass-based substrates. For the devices based on emitters **CN1**, **CN2**, **CN3**, **CN4**, and **CN5**, the theoretical η_ext_ values are expected in the ranges of 15.2–22.8, 10.2–15.3, 0.8–1.2, 5.4–8.1, and 9.8–14.7%, respectively. This statement is in very good agreement with the increased EQE value up to 15.9% for the optimized device d1b when concentration of emitter **CN1** of 20 wt % was used (Table 3). When novel functional layers and host materials are available,^56^ optimization of EL performance of the developed compounds are expected.

Finally, it should be noted that the similarity of EL performances of non-doped and doped OLEDs especially those based on emitter **CN1** well proved the proposed concept of minimization of effects of solid-state solvation and conformation disorder on emission properties of TADF emitters. To additionally support our molecular design strategy, we compared maxima of EL spectra of non-doped (**λ**_**nd**_) and doped (λ_**d**_) OLEDs based on the emitters designed using the multiple donor–acceptor strategy (this work) and on some other multi-carbazole-based emitters (Table S5). It should be noted that compound CN1 showed state-of-art performance with respect to the solid-state solvation and conformation disorder (the lowest difference between the wavelengths of maxima of EL spectra of non-doped and doped OLEDs of 4 nm was observed).

## 4 Conclusions

High-performance TADF materials were designed using the multiple donor–acceptor strategy. Highly efficient blue TADF emitters based on four 3,6-di-*tert*-butylcarbazole moieties as donors and two electron acceptors, one of which was trifluorotoluene unit were obtained. The effect of the additional acceptor moiety attached through para position of trifluorotoluene unit on the properties of the emitters was studied. The co-existence of through-space and TBCT ensured very small singlet-triplet energy gaps of 8–22 meV. The highly twisted structure of the multiple donor–acceptor-type derivatives 3,6-di-*tert*-butylcarbazole and trifluorotoluene lead to high PLQYs (up to 76% in solid state) and to the additional channels of RISC. The nearly identical first singlet and triplet excited-state configurations were observed for the synthesized derivative of 3,6-di-*tert*-butylcarbazole and trifluorotoluene with the additional pyrimidine acceptor moiety (**CN1**)**.** The singlet-triplet energy gap of this compound was lower than 0.03 eV due to the multiple RISC channels through either electronic or vibrational couplings. **CN1** exhibited a high RISC rate constant of *ca.* 7 × 10^5^ s^–1^. The fabricated sky-blue TADF non-doped and doped OLEDs based on **CN1** exhibited high maximum EQE values of 8.4 and 15.9%, maximum brightness values of 19 735 and 39 226 cd/m^2^, and maximum current efficiencies of 42.6 and 20.5 cd/A, respectively. The multi-donor–acceptor concept is appropriate for the further development of organic emitters with the absence of effects of solid-state solvation and conformation disorder on TADF emission properties.

## Supporting Information Available

“Instrumental” section; additional data and descriptions on thermal, electrochemical, photophysical, electrooptical, EL, and theoretical investigations of the studied compounds; molecular structures of multi-carbazole-based emitters and a comparison of their EL spectra in doped and non-doped devices; PL decay curve fitting parameters; rate constants of deoxygenated toluene solutions of compounds; PL spectra; and optimized molecular structure data for **CN1–5** compounds (PDF)
