# Supplementary material for: Ornamenting of Blue Thermally Activated Delayed Fluorescence Emitters by Anchor Groups for the Minimization of Solid-State Solvation and Conformation Disorder Corollaries in Non-Doped and Doped Organic Light-Emitting Diodes
Source: ACS Appl Mater Interfaces. 2022 Aug 24;14(35):40158–72. doi: 10.1021/acsami.2c12475 (PMC9460442; doi:10.1021/acsami.2c12475)
Supplement: Supplementary file 1 — am2c12475_si_001.pdf [file am2c12475_si_001.pdf]

Supporting Information

Ornamenting of blue TADF emitters by anchor groups for minimization of solid-state solvation and conformation disorder corollaries in non-doped and doped OLEDs

Malek Mahmoudi [a], Dalius Gudeika [a], Stepan Kutsiy [b], Jurate Simokaitiene [a], Rita Butkute [a], Levani Skhirtladze [a], Kai Lin Woon[c], Dmytro Volyniuk [a,*], Juozas Vidas Grazulevicius[a,*]

[a] *Department of Polymer Chemistry and Technology, Kaunas University of Technology, Radvilenu pl.19, LT-50254, Kaunas, Lithuania;*

[b] *Department of Electronic Devices, Lviv Polytechnic National University, S. Bandera 12, 79013, Lviv, Ukraine*

[c] *Low Dimensional Material Research Centre, Department of Physics, University Malaya, Kuala Lumpur, Malaysia.*

e-mail: dmytro.volyniuk@ktu.lt; juozas.grazulevicius@ktu.lt.

Contents

Instrumental........S1

Figures and tables........S4

AIEE........S14

Temperature dependent steady state and time resoled PL measurements........S16

Optimized Molecular structure data for CN1-5 compounds........S21

References........S41

Instrumental

^1H (400 MHz) and ^{13}C (101 MHz) NMR spectra were recorded on a Varian Unity Inova 300 apparatus at ambient temperature; spectra were analysed with the MestreNova program package. Infrared (IR), melting points, thermogravimetric analysis, differential scanning calorimetry (DSC) measurements were carried out as described earlier.[1] Mass spectra were recorded on a Waters ZQ 2000 analytical system. Elemental analysis was performed with an Exeter Analytical CE-440 Elemental Analyzer. The gas phase structures of the compounds in the ground electronic state were optimized at the DFT level using the B3LYP hybrid functional and 6-31G(d,p) basis set. The geometries of the S_1 and T_1 states were optimized using TDDFT gradients at the B3LYP/6-31G(d,p) level. All computations were carried out with the Gaussian 16 program package.[2] Cyclic voltammetry (CV) measurements were carried out as described earlier. [3]

All dilute solutions of samples were prepared with concentrations of 10^{-5} M for absorption and emission study. Absorption spectra were recorded at Room temperature (RT) using the UV–VIS–NIR Avantes (AvaSpec-2048XL) spectrophotometer with a 1 cm quartz cuvette. Steady state emission and time-resolved emission spectra were recorded at RT using an Edinburgh Instruments FLS980 spectrometer. Samples were excited at 330 nm using a Xenon lamp for steady-state measurements and at 374 nm using the PicoQuant LDH-D-C-375 laser for time-resolved emission spectra. FLS980 integrating sphere was used for recording the values of photoluminescence quantum yields (PLQY) by an absolute method at room temperature. PLQY values of the neat films in oxygen-free conditions were obtained by first performing measurements in the air using an integrating sphere, and then evaluating the increase of PL intensity by placing the films in a vacuum cryostat equipped with a turbo-molecular pump and capable of achieving 10^{-5} Torr pressure. Furthermore, by using the same method, the PL quantum yields for synthesized compounds in toluene under ambient and degassed conditions were measured. For preparing

oxygenated solutions, samples were bubbled using a compressed oxygen capsule for 10 minutes and degassed solutions were prepared via bubbling argon inert gas for 10 minutes using a quartz cuvette cell. The singlet-triplet energy splitting (ΔE_{ST}) was determined from the energy difference between the onsets of the fluorescence and phosphorescence spectra of the THF solutions recorded at 77 K using UV–VIS–NIR Avantes (AvaSpec-2048XL) spectrophotometer. An integral nitrogen reservoir cryostat Optistat DN2 providing a controlled low temperature exchange gas environment was used for the characterization of photophysical properties of the samples from 77 to 300 K in inert atmosphere (N_2). Ionization potentials (IP_{PE}) of designed compounds in solid-state were estimated by the electron photoemission spectroscopy in air. [4] Fluorine doped tin oxide (FTO) coated substrates were applied for the preparation of samples for photoelectron emission spectrometry. The layers of the compounds were vacuum deposited onto the substrates. Photoelectron emission spectra were investigated using Keithley 6517B electrometer/high resistance meter, ASBN-D130-CM deep UV deuterium light source and CM110 1/8 m monochromator.

OLEDs were fabricated by step-by-step thermal vacuum evaporation method. Kurt J. Lesker devices in-built in a MB EcoVap4G glove box were used for the deposition of organic and metal layers onto pre-cleaned patterned ITO-coated glass substrates under the vacuum of 2×10^{-6} mBar with the rate of ~ 0.1-0.2 nm/s for organic layers. Simultaneous electroluminescent characterization of OLEDs in the air at room temperature without passivation was done using Keithley 6517B electrometer, Keithley 2400C SourceMeter, calibrated PH100-Si-HA-D0 photodiode and a PC-Based Power and Energy Monitor 11S-LINK.

Figures and tables

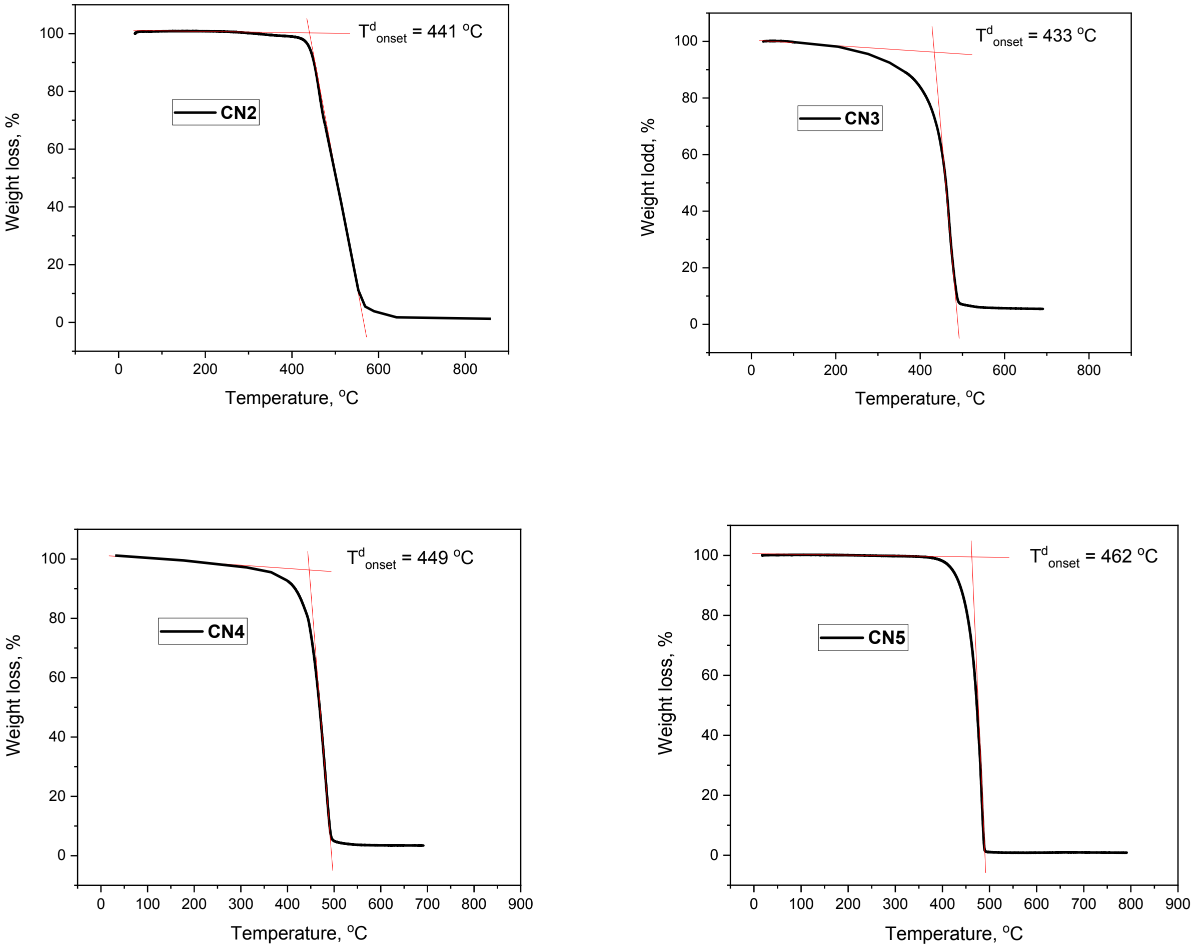

Figure S1. TGA curves of compounds **CN2-5** (scan rate 20 °C/min).

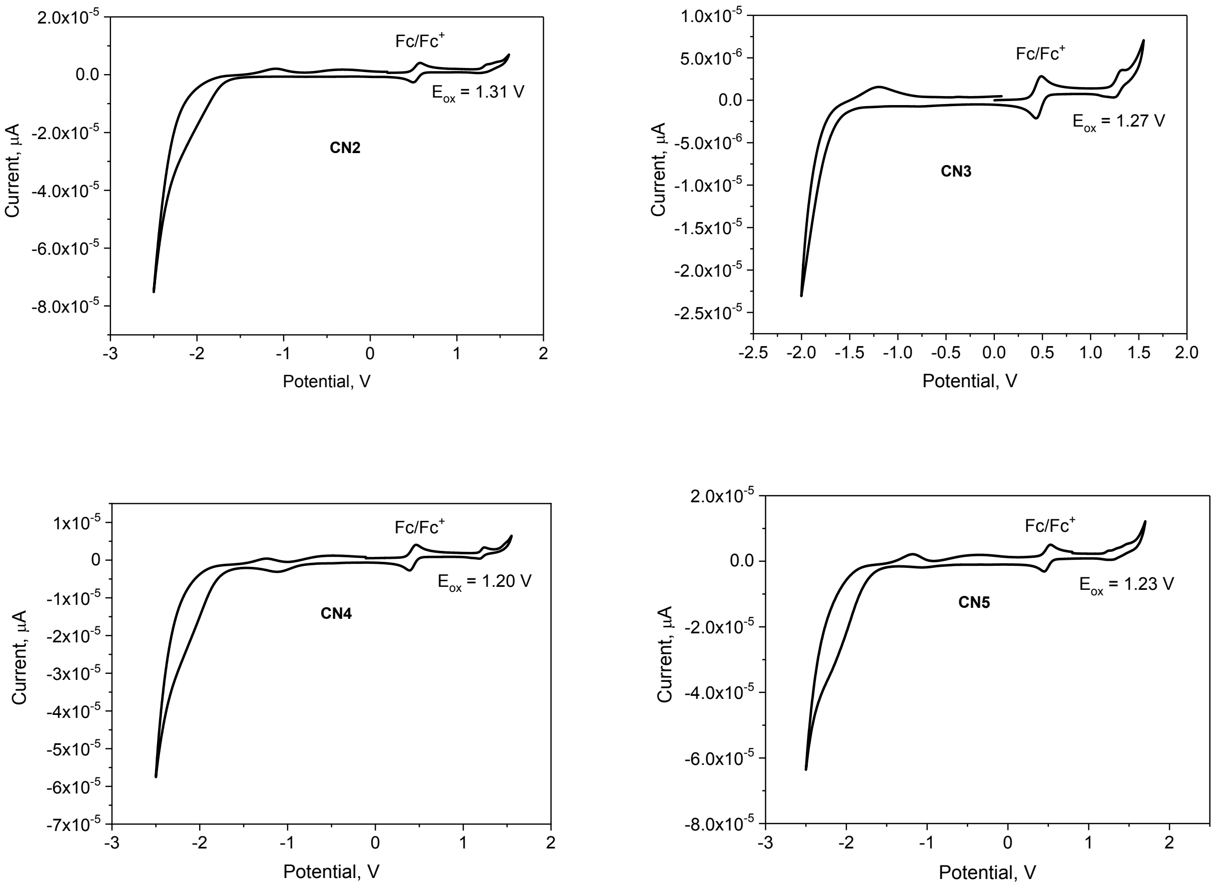

Figure S2. Cyclic voltammograms of dilute solutions of **CN1-5** in dichloromethane (scan rate 100 mV/s).

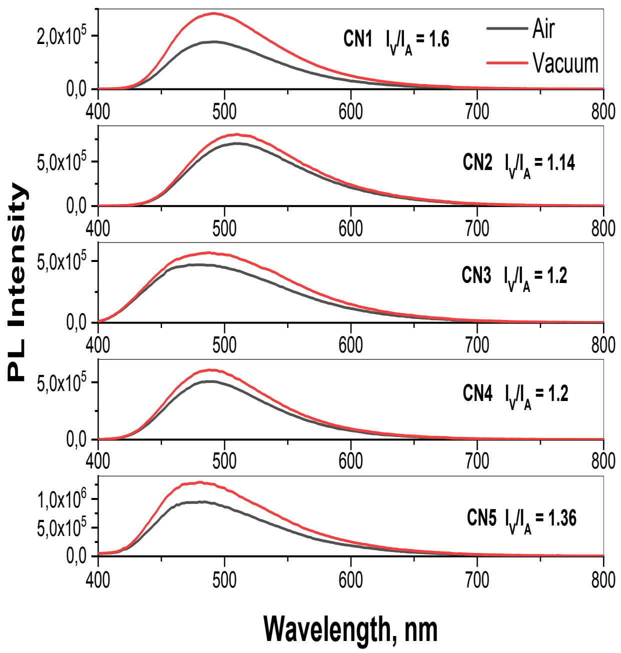

Figure S3. PL spectra of the films of compounds **CN1-5** under air and vacuum.

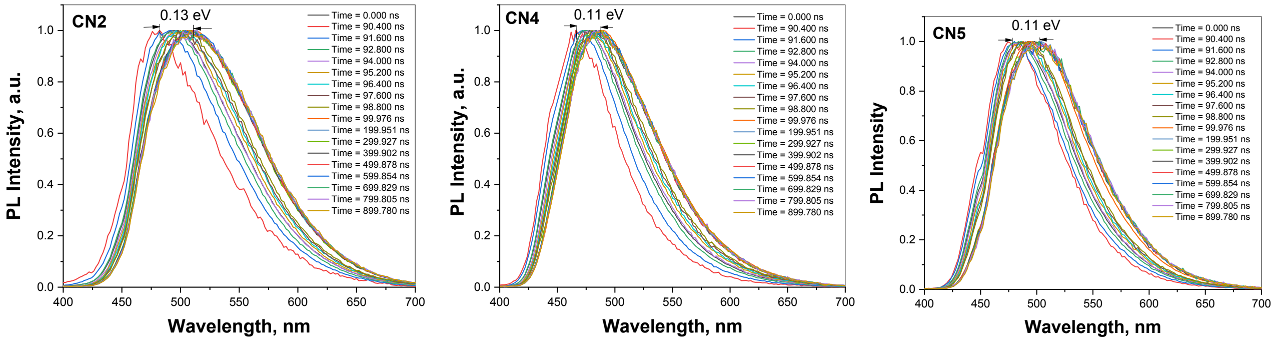

Figure S4. TRES data in 2D plots and the relative PL spectral shifts for the compounds **CN2**, **CN4** and **CN5**.

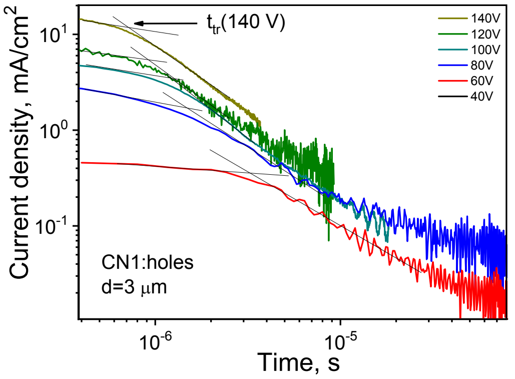

Current density, mA/cm2
Time, s
tr(140 V)
140V
120V
100V
80V
60V
40V
CN1:holes
d=3 μm

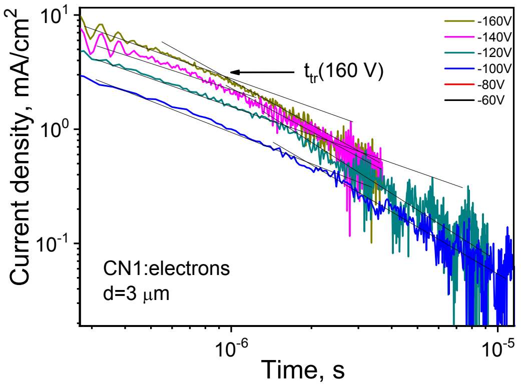

Current density, mA/cm2
Time, s
tr(160 V)
-160V
-140V
-120V
-100V
-80V
-60V
CN1:electrons
d=3 μm

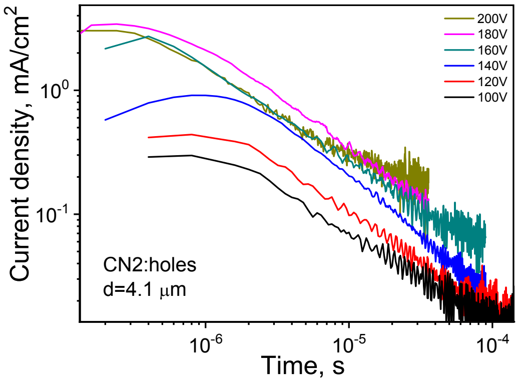

Current density, mA/cm2
Time, s
200V
180V
160V
140V
120V
100V
CN2:holes
d=4.1 μm

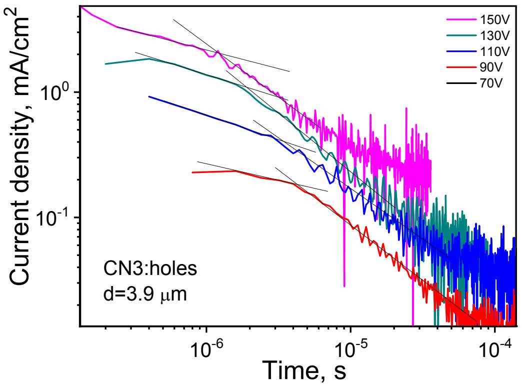

Current density, mA/cm2
Time, s
150V
130V
110V
90V
70V
CN3:holes
d=3.9 μm

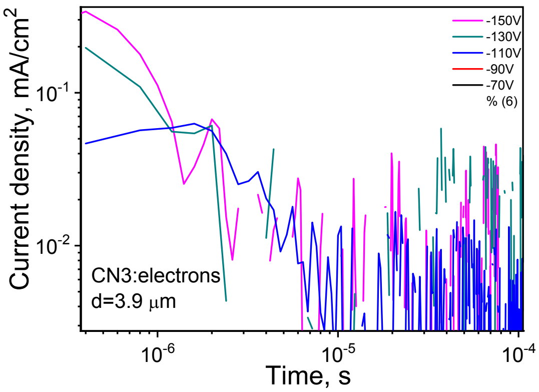

Current density, mA/cm2
Time, s
-150V
-130V
-110V
-90V
-70V
% (6)
CN3:electrons
d=3.9 μm

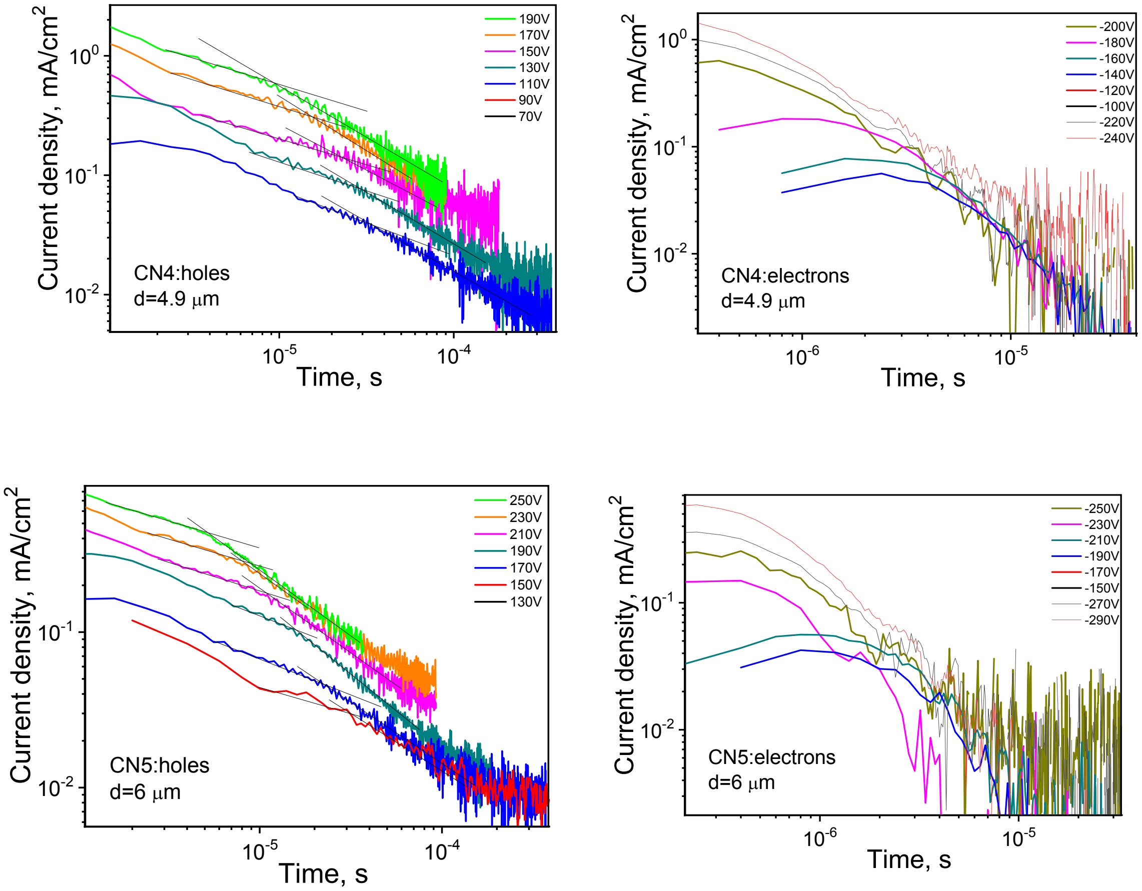

Figure S5. TOF signals for vacuum deposited films **CN1-5** at positive (for holes) and negative (for electrons) applied voltages at ITO electrode.

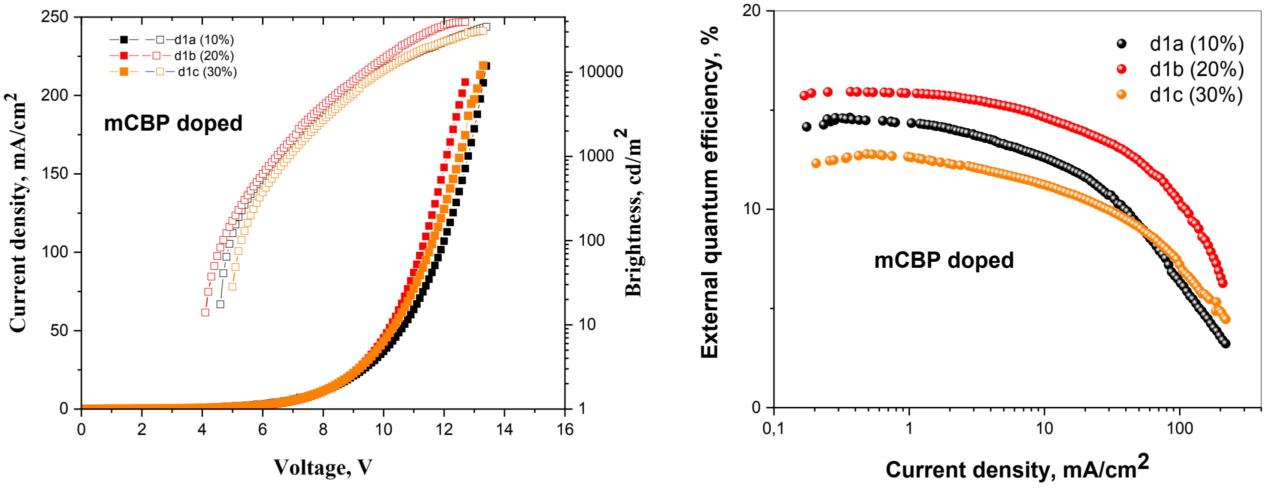

(a) (b)

Figure S6. Current density and brightness versus voltage curves (a) and external quantum efficiencies versus current density of fabricated doped devices at different concentrations of **CN1** (b).

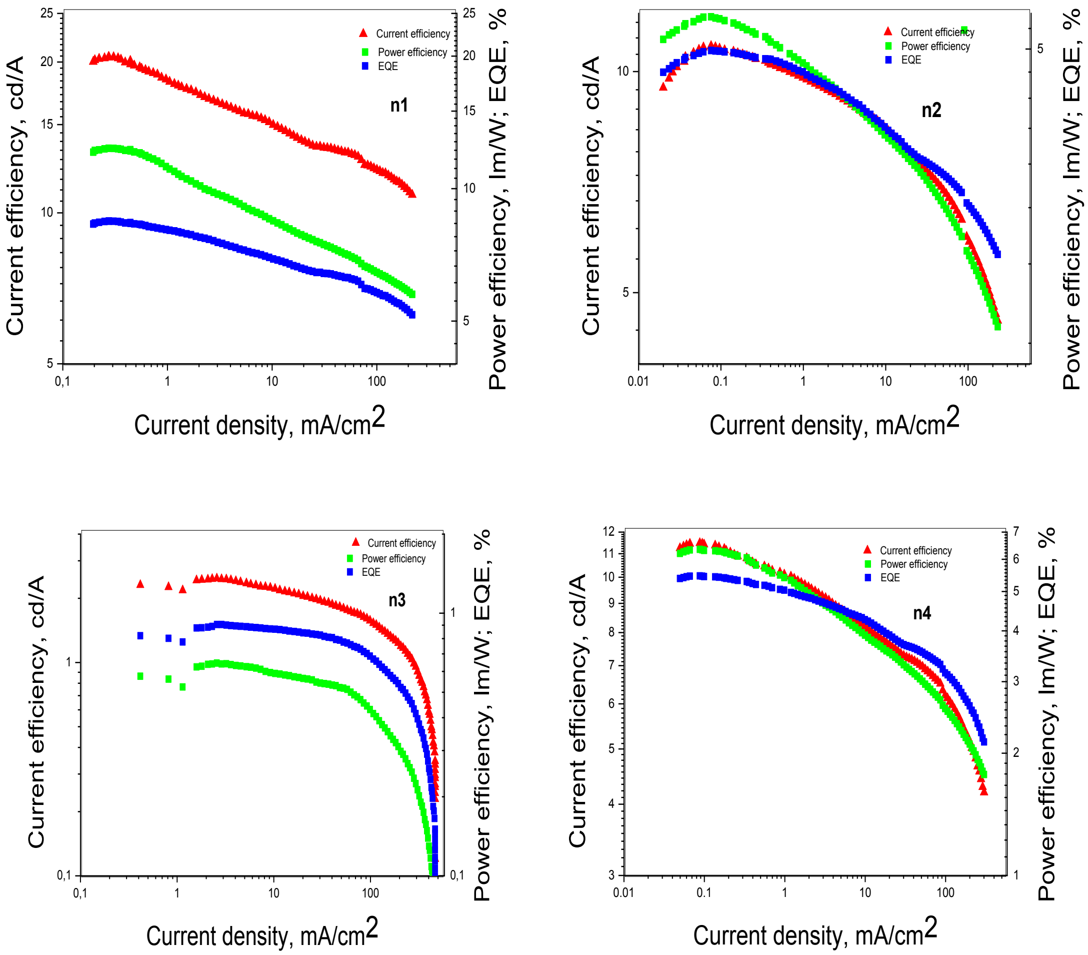

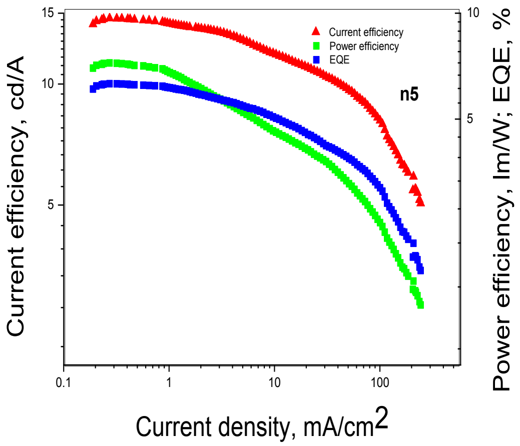

Figure S7. Current, power and external quantum efficiencies of non-doped devices

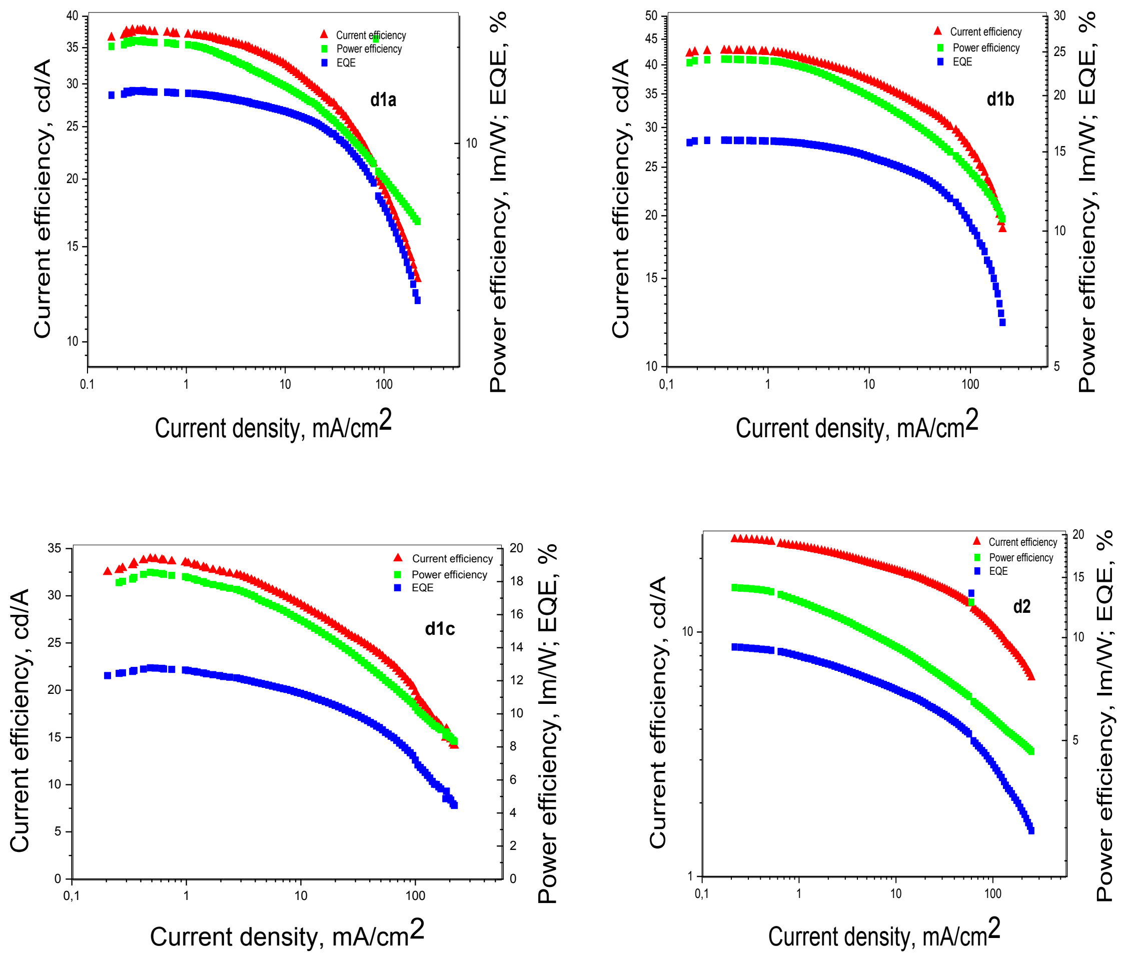

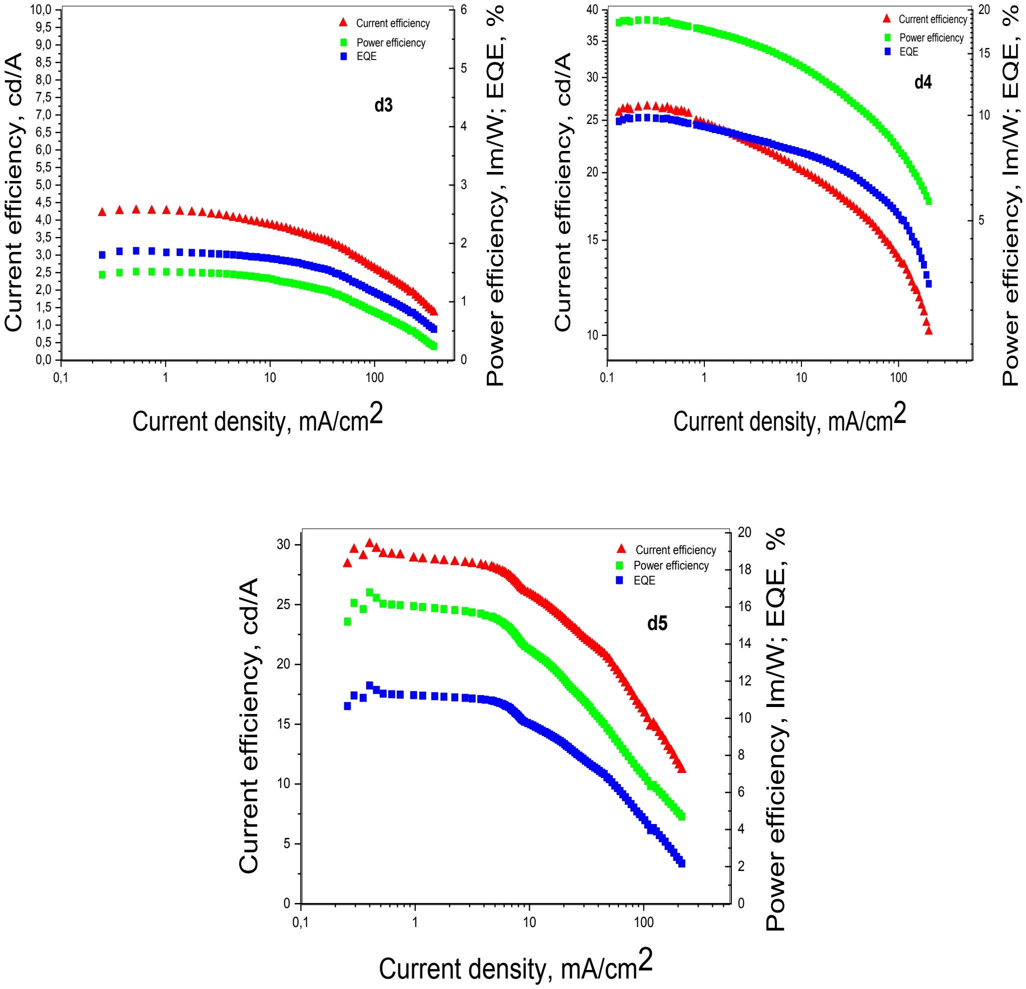

Figure S8. Current, power and external quantum efficiencies of mCBP-doped devices

Table S1. PL decay curve fitting parameters of air equilibrated, degassed and oxygenated toluene solution of compounds

	τ_1 (ns)/(percentage)	τ_2 (ns) / (percentage)	X^2
**CN1**			
Degassed	15(19.92)	2153(80.08)	1.2
**CN2**			
Degassed	16(72.56)	92(27.44)	1.0
**CN3**			
Degassed	2(99)	11(1)	1.0

**CN4**			
Degassed	10(46.53)	2147(53.47)	1.0
**CN5**			
Degassed	17(54.06)	24(45.94)	1.1

Table S2. PL decay curve fitting parameters of neat films of compounds under air and vacuum.

	τ_1 (ns)/(percentage)	τ_2 (ns) / (percentage)	X^2
**CN1**			
Air	9(40.72)	1493(59.28)	1.0
Vacuum	9(31.42)	2373(68.58)	1.1
**CN2**			
Air	13(36.04)	1223(63.96)	1.0
Vacuum	13(32.15)	1496(67.75)	1.2
**CN3**			
Air	16(58.02)	607(41.98)	1.0
Vacuum	23(57.11)	774(42.89)	1.3
**CN4**			
Air	19(62.36)	1216(37.64)	1.0
Vacuum	23(48.68)	1791(51.32)	1.2
**CN5**			
Air	12(34.65)	1986(65.35)	1.2
Vacuum	21(14.84)	2879(85.16)	1.3

Table S3. Compound **CN1** transient PL decay fitting parameters recorded at different temperatures

**CN1** TADF (20 μs)			
Temperature	τ_1 (ns)/(percentage)	τ_2 (ns) / (percentage)	X^2
77 K	41(85.27)	1005(14.73)	1.3
100 K	37(62.89)	3346(37.11)	1.3
120 K	30(67.04)	2407 (32.96)	1.1
140 K	29(65.82)	2196 (34.18)	1.3
160 K	28(64.91)	2056(35.09)	1.1
180 K	27(59.32)	2073(40.68)	1.1
200 K	26(55.16)	1846(44.84)	1.1
220 K	19(64.60)	1633(35.40)	1.2
240 K	16(53.71)	1363(46.29)	1.1
260 K	16(53.97)	1334(46.03)	1.2
280 K	16(52.69)	1322(47.31)	1.2
300 K	14(49.25)	1110(50.75)	1.2

Table S4. Rate constants of deoxygenated toluene solutions of compounds

Parameters	Sample / equation	**CN1**	**CN2**	**CN3**	**CN4**	**CN5**
PLQY(%)	neat films	44	12	8	17	13
η_{PF}	$\eta_{PF} = \eta_{\mathrm{PLQY}} * \mathrm{PF}(\%)/100(\%)$	0.09	0.09	0.08	0.08	0.07
η_{DF}	$\eta_{DF} = \eta_{\mathrm{PLQY}} * \mathrm{DF}(\%)/100(\%)$	0.35	0.03	0.00	0.09	0.06
τ_{PF}, ns (%)	from PL decay fitting by I=A+B1exp(-t/τ_{PF})+B2exp(-t/τ_{DF})	15(20)	16(72)	2(99)	10(46)	17(54)
τ_{DF}, µs (%)		2.153 (80)	0.09 (28)	0.012 (1)	2.147 (54)	0.024 (46)
k_{PF}, s^{-1}	$k_{PF} = \frac{\eta_{PF}}{\tau_{PF}}$	5.8×10^6	5.5×10^6	3.9×10^7	7.9×10^6	4.1×10^6
k_{ISC}, s^{-1}	$k_{ISC}=\frac{\eta_{DF}}{\eta_{PF}+\eta_{DF}}k_{PF}$	4.7×10^6	1.5×10^6	3.9×10^5	4.2×10^6	1.9×10^6
k_{DF}, s^{-1}	$k_{DF} = \frac{\eta_{DF}}{\tau_{DF}}$	1.6×10^5	3.6×10^5	6.6×10^4	4.3×10^4	3×10^6
k_{RISC}, s^{-1}	$k_{RISC} = \frac{\eta_{DF}}{\eta_{PF}} \cdot \frac{k_{PF}\,.\,k_{DF}}{k_{ISC}}$	8×10^5	5×10^5	6.7×10^4	9.3×10^4	5.5×10^6

Table S5. Molecular structures of multi-carbazole-based emitters and comparison of EL spectra of doped and non-doped devices.

Compound	Molecular structure	**λ_{nd}, nm**	**λ_{d}, nm**	**$\Delta\lambda_{n-nd}$, nm**	Ref.
CN1	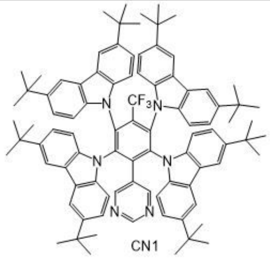	481	477	4	This work
5TCzBN	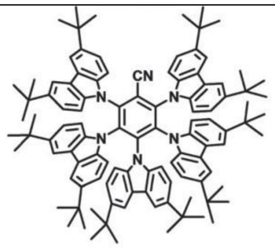	480*	490	10	[5]
2PhCz2CzBN	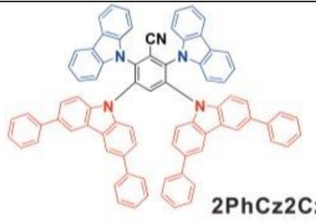	480	464	16	[6]

2tCz2CzBN	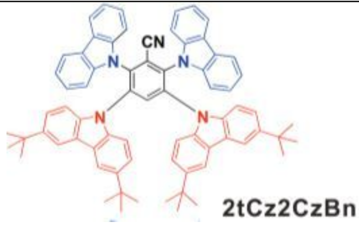	470	464	6	6
CNCN	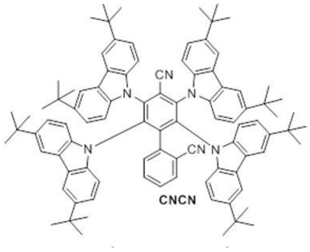	495	468	27	7
5tCzMeB	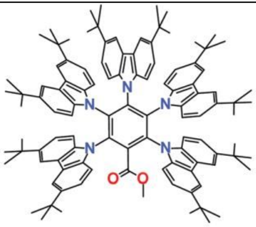	481	488	7	8
5tCzBP	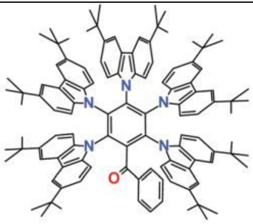	497	508	11	8

* Taken from the photoluminescence spectrum of the film of non-doped compound.

AIEE

PL spectra of the dispersions of **CN1-CN5** in the THF-water mixtures with various water fractions (f_w) from 0 to 90% were recorded (Figure S9). Except CN3, emissive aggregates of the studied compounds were formed at the certain concentrations of water (f_w of 50-60%) highlighting AIEE phenomenon. Relative dependences of PL intensities of the dispersions of compounds CN1-CN5 versus water fractions are shown in Figures S9b. In the case of the dispersion of compound CN5, the emission intensity constantly increased with the increase in water fraction, due to the increasing amount of aggregates. The dispersions of compounds CN1, CN2 and CN4 showed maximum emission intensity and higher blue shift at f_w of 60%. The further increase of f_w induced decrease of PL intensity. This observation is attributed to draping down of aggregates in quartz cuvette. The similar observation was previously reported for compounds exhibiting AIEE [9].

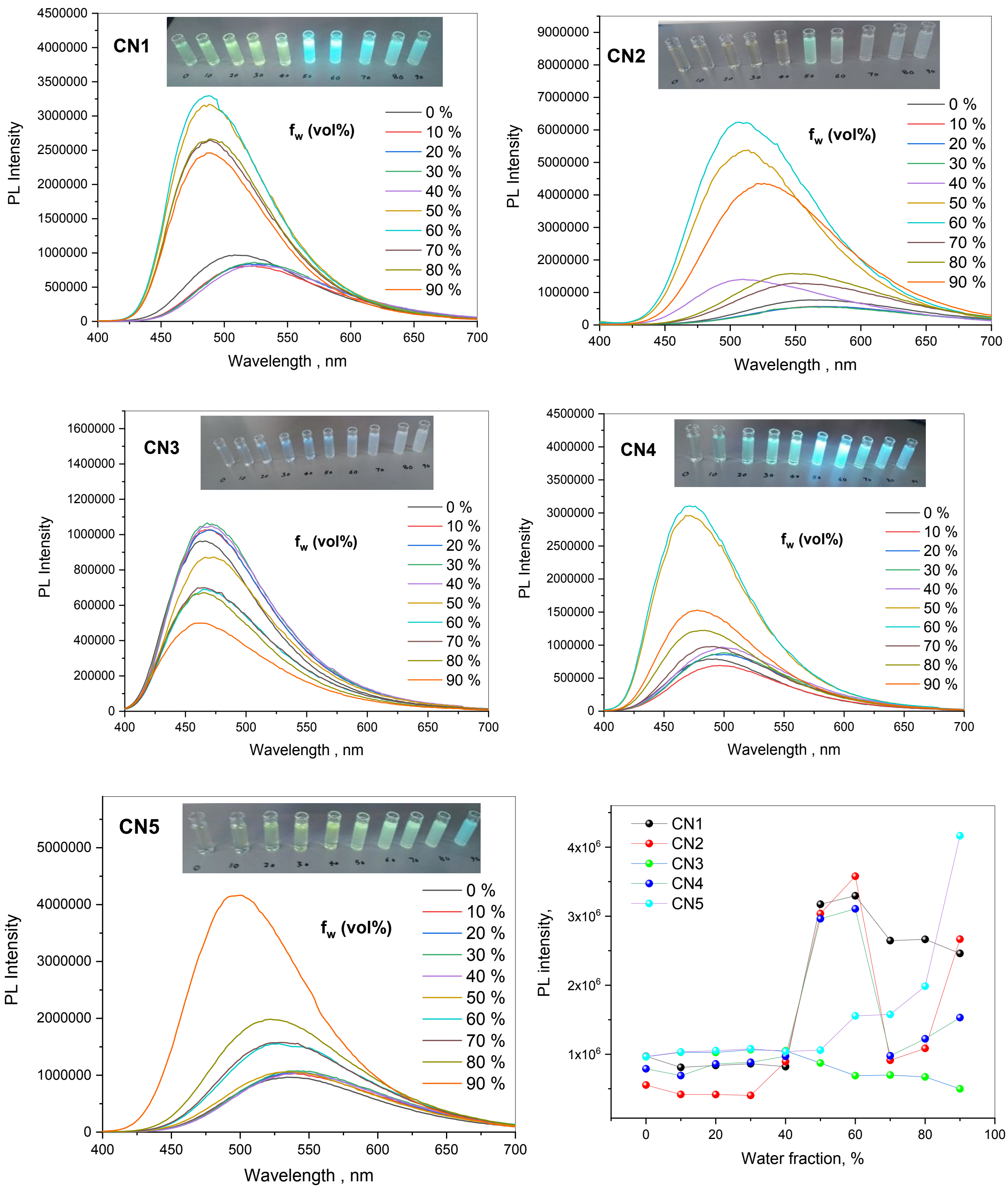

(a) (b)

Figure S9. PL spectra of the dispersions of compounds **CN1-CN5** in THF/water mixtures with various water fractions (a). Inset shows photographs of the dispersions in THF/water mixtures under UV excitation. PL maxima intensities versus water volume fractions for the dispersions of the compounds in the mixtures of THF and water (b).

Temperature dependent steady state and time resoled PL measurements.

PL spectra and PL decay curves of the neat films were recorded under inert atmosphere at the different temperatures (Figure S10, S11). As it was expected, the typical TADF decay curves were observed. They showed the prompt fluorescence component in nanosecond range and delayed fluorescence component in microsecond range. The intensity of delayed fluorescence constantly grew up with the increase of temperature from 77 to 260 K proving the TADF nature of emission. The rate constants k_{ISC} and k_{RISC} were calculated at the different temperatures by taking lifetimes of prompt florescence and delayed fluorescence from the exponential fitting of TADF decays recorded at the different temperatures (Table S6).

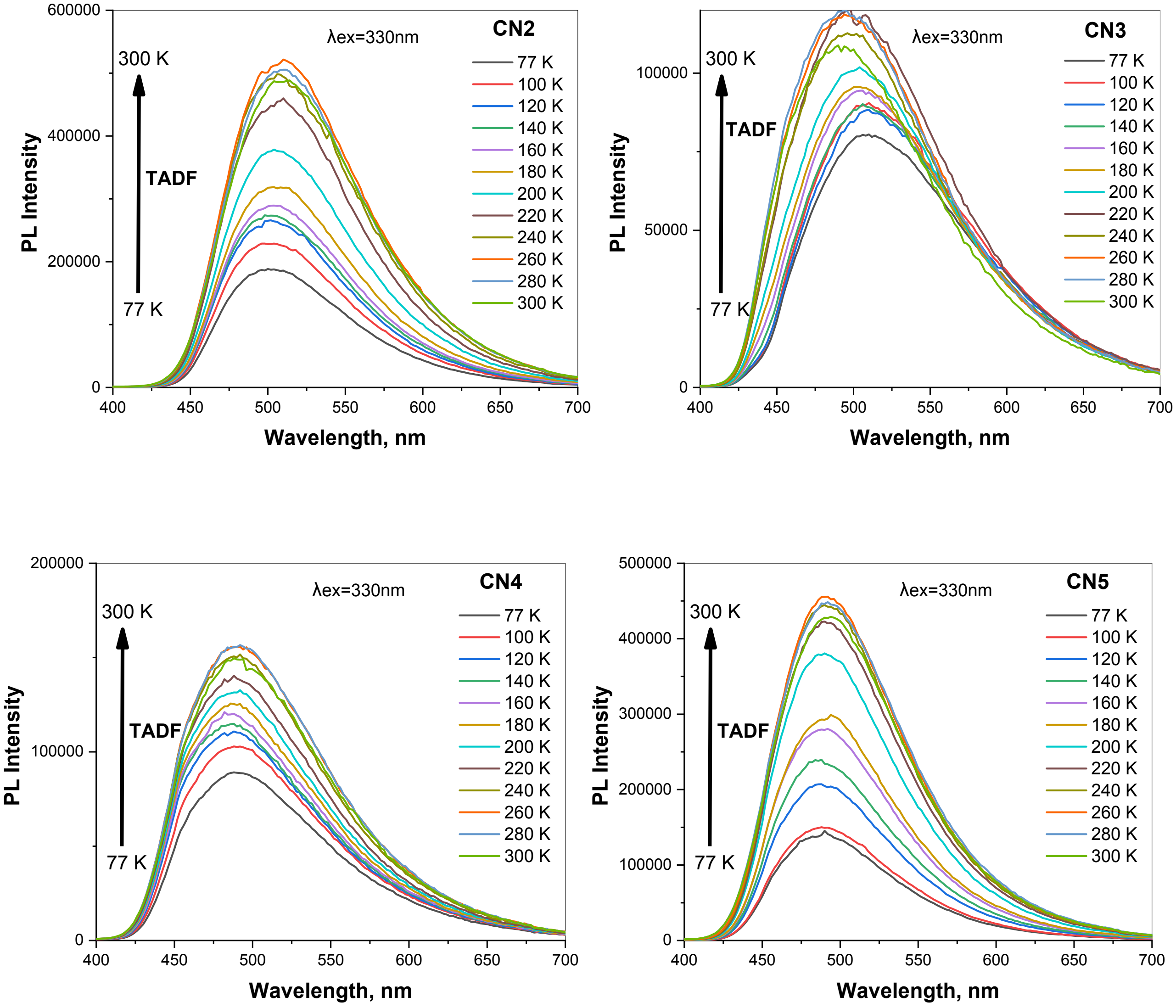

Figure S10. PL spectra of neat films of **CN2-CN5** recorded at the different temperatures.

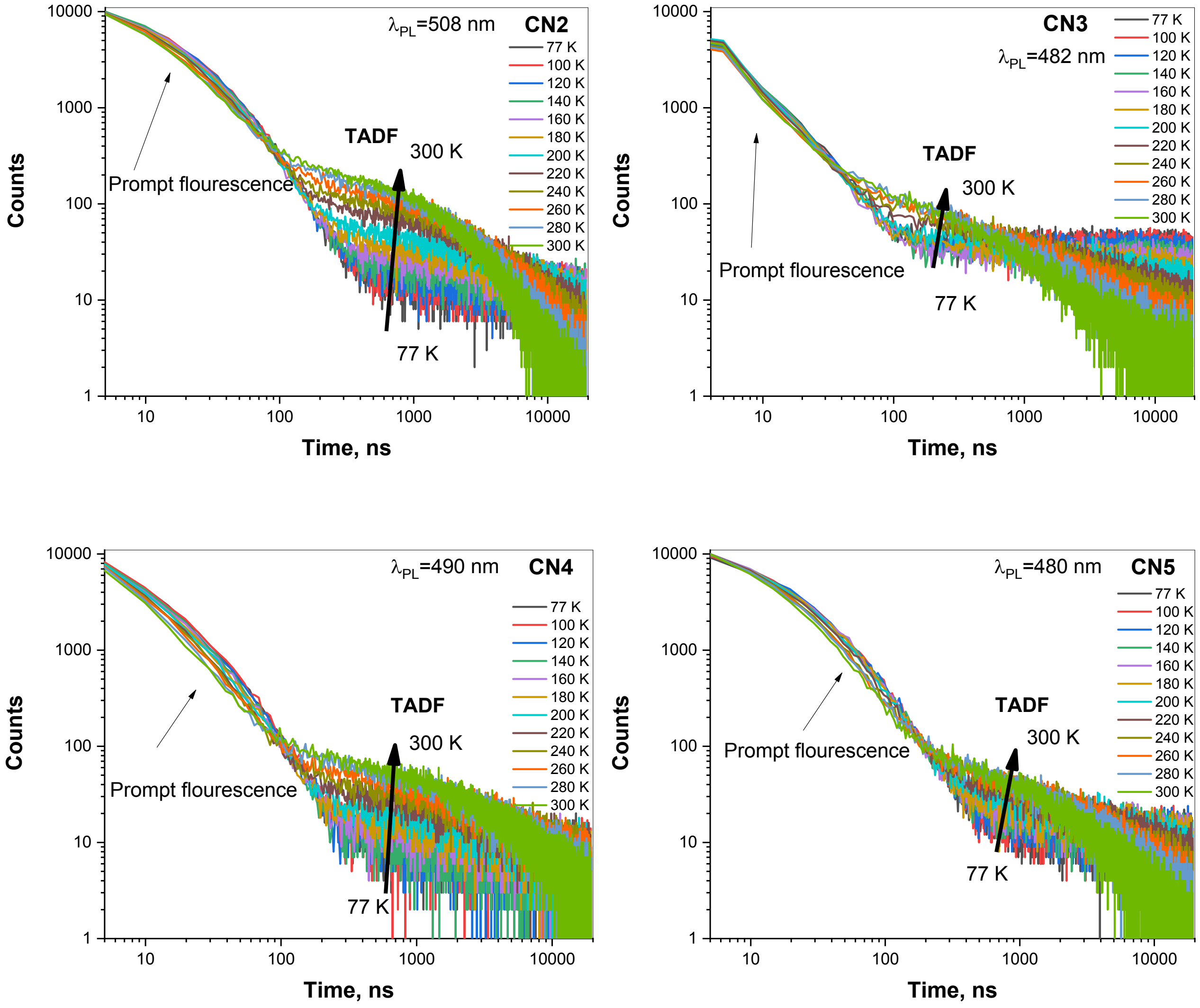

Figure S11. PL decay curves of neat films of **CN2-CN5** recorded at the different temperatures.

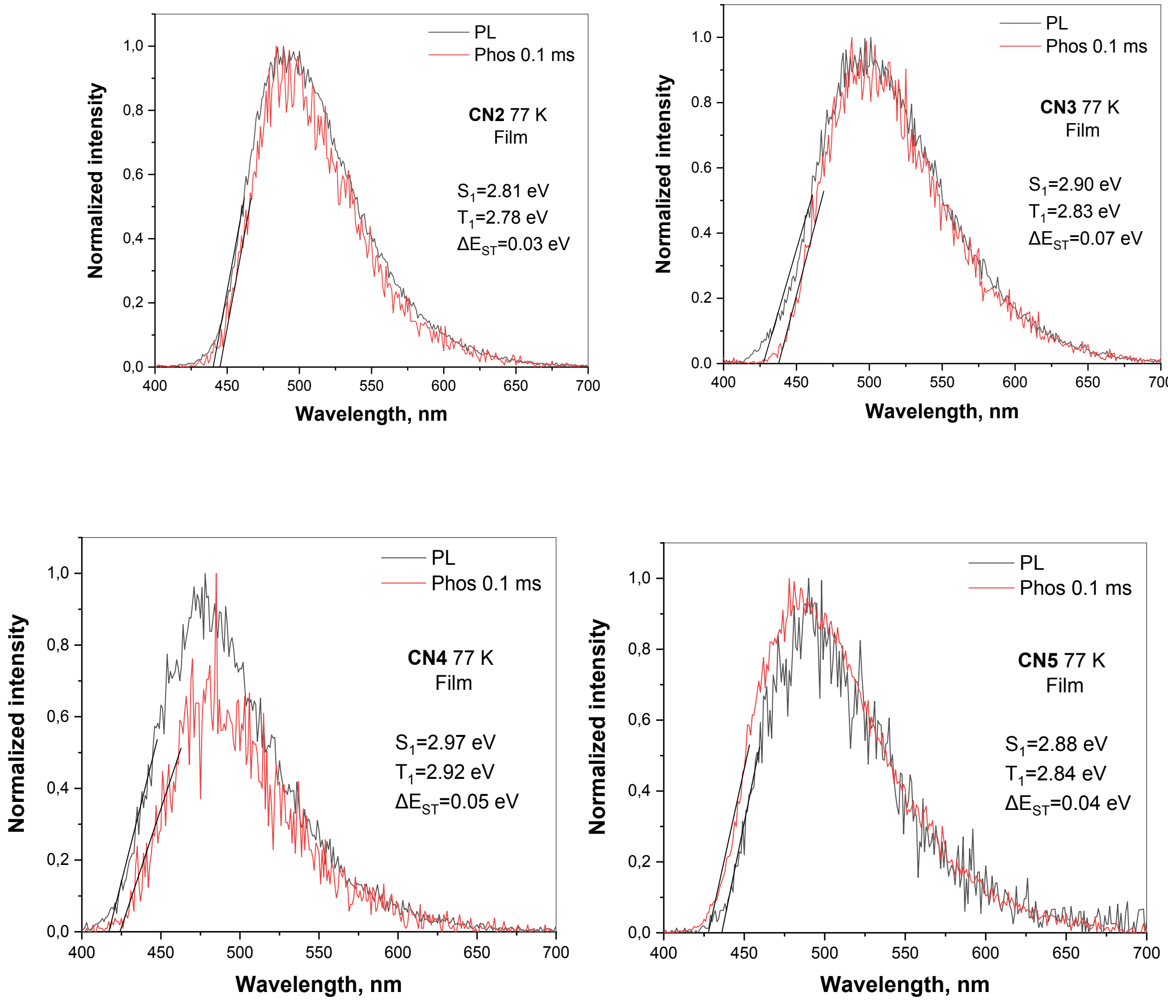

Figure S12. PL and phosphorescence spectra of the neat films of compounds **CN2-CN5** recorded at 77 K.

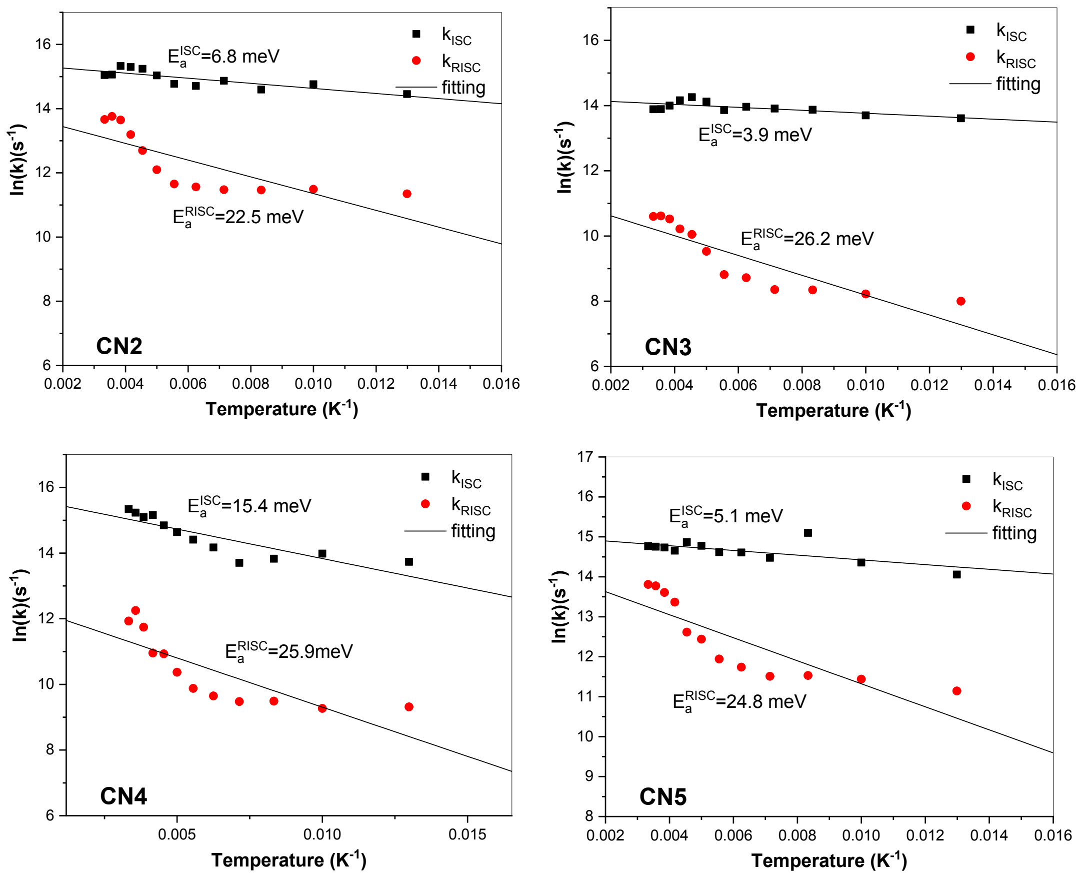

Figure S13. The temperature dependences of k_{ISC} and k_{RISC} for neat films of compounds **CN2-5**.

Table S6. The fitting parameters of PL decays of the film of **CN2-5** recorded at the different temperatures.

CN2			
Temperature	τ_1 (ns)/(percentage)	τ_2 (ns) / (percentage)	χ^2
77 K	25.32(61.38)	1543(38.62)	1.2
100 K	21.37(65.75)	1327(34.25)	1.2
120 K	30.14(63.66)	1681(36.34)	1.3
140 K	24.19(61.52)	1878(38.48)	1.3
160 K	31.48(54.05)	2504(45.95)	1.3
180 K	32.73(50.49)	2907(49.51)	1.3
200 K	29.56(48.22)	2416(51.78)	1.3
220 K	26.54(36.80)	2512(63.20)	1.3
240 K	24.95(29.83)	2267(70.17)	1.3
260 K	24.05(28.40)	1643(71.60)	1.3
280 K	25.33(21.06)	2101(78.94)	1.3
300 K	24.30(20.51)	2312(79.49)	1.3

CN3			
Temperature	τ_1 (ns)/(percentage)	τ_2 (ns) / (percentage)	χ^2
77 K	8.02(71.17)	4357.55(28.83)	1.1
100 K	9.42(65.73)	5000(34.27)	1.3
120 K	9.61(45.19)	1000(54.81)	1.3
140 K	8.76(50.78)	7952(49.22)	1.3
160 K	8.36(50.19)	5935(49.81)	1.3
180 K	8.45(44.01)	5236(55.99)	1.3
200 K	7.26(41.54)	4092(58.46)	1.3
220 K	7.1(37.73)	3361(62.27)	1.3
240 K	7.32(35.88)	2785(64.12)	1.3
260 K	8.21(31.59)	2566(68.41)	1.3
280 K	9.46(31.50)	2461(68.50)	1.3
300 K	8.48(31.18)	2272(68.82)	1.3

CN4			
Temperature	τ_1 (ns)/(percentage)	τ_2 (ns) / (percentage)	χ^2
77 K	8.55(82.38)	3045(17.62)	1.3
100 K	8.41(83.75)	3357(16.25)	1.3
120 K	8.16(81.21)	3478(18.79)	1.3
140 K	37.28(79.90)	4015(20.10)	1.3
160 K	27.70(76.06)	4391(23.94)	1.3
180 K	24.06(73.88)	4105(26.12)	1.3
200 K	23.27(66.22)	3776(33.78)	1.3
220 K	21.390(61.80)	2837(38.20)	1.3
240 K	17.32(57.58)	3497(42.42)	1.3
260 K	19.52(51.49)	2123(48.51)	1.3
280 K	17.04(50.87)	1307(49.13)	1.3
300 K	14.61(47.08)	1988(52.92)	1.3

CN5			
Temperature	τ_1 (ns)/(percentage)	τ_2 (ns) / (percentage)	χ^2
77 K	24.35(52.14)	2150(47.86)	1.3
100 K	32.58(50.19)	2496(49.81)	1.3
120 K	25.87(52.15)	3455(47.85)	1.3
140 K	49(51.40)	3611(48.60)	1.2
160 K	45.10(44.51)	3972(55.49)	1.3
180 K	45.51(43.21)	3547(56.79)	1.3
200 K	40.53(37.12)	3050(62.88)	1.3
220 K	37.27(34.79)	2926(65.21)	1.3
240 K	36.99(23.64)	2374(76.36)	1.3
260 K	34.35(22.35)	2795(77.65)	1.3
280 K	34.34(22.44)	2490(77.56)	1.3
300 K	32.86(22.47)	2010(77.53)	1.1

Optimized Molecular structure data for the CN1-5 compounds

The calculation was performed using Terachem 1.93 software [10] and a Graphic Processing Unit server that had 64 GB RAM installed to support eight Tesla K10 graphic cards at the Universiti Malaya Data Intensive Computing Center. The results were visualized using Visual Molecular Dynamics (VMD) software. Density functional theory (DFT) using range-separated hybrid functional LC-ωPBEh was used to obtain the ground state molecular geometry at def2-svp basis set. ω was tuned using the golden ratio algorithm under polarizable continuum model (PCM) with a dielectric constant of 2.38 corresponding to toluene and solvent radius of 3.48Å.

Optimized Molecular structure data for **CN1** using Terachem 1.93 software

E=-4.1478719217747730e+03a.u

H	-4.3894147381	6.4827340553	5.4479079116
C	-3.8512783646	5.8003479434	4.7688750801
H	-3.0288595558	6.3731074347	4.3086479380
H	-3.4109619816	4.9994757618	5.3862376181
C	-4.8194321968	5.2429099401	3.7197666646
C	-5.4088524573	6.4235524734	2.9275758671
H	-6.1403569184	6.0839443542	2.1763320515
H	-4.6168169405	6.9784822310	2.3976278981
H	-5.9260714991	7.1249874423	3.6054029048
C	-5.9540163540	4.5068055390	4.4551136754
H	-6.7185580830	4.1320891430	3.7555166924
H	-6.4581306539	5.1820044438	5.1686418622
H	-5.5642763703	3.6428759743	5.0200218574
C	-4.1301387607	4.2679776131	2.7532617289
C	-2.7561044639	3.9595449976	2.8357342778
H	-2.1378981991	4.4435260079	3.5952413452
C	-2.1343648897	3.0489772559	1.9781748370
H	-1.0647226013	2.8426491939	2.0759480888
C	-2.9152096497	2.4256281923	1.0069230443
N	-2.5761196398	1.4497426205	0.0661926075
C	-3.7113896446	1.1533185187	-0.6899532729
C	-4.7979331190	1.9162105705	-0.2095513101
C	-4.2927211557	2.7238768544	0.8805518739
C	-4.8843013052	3.6396249440	1.7529978464
H	-5.9533341735	3.8535458186	1.6521188370
C	-6.0575812177	1.7932936860	-0.8093716631
H	-6.8875243807	2.3857240339	-0.4169887195
C	-6.2438073923	0.9293399704	-1.8915789496
C	-5.1237461027	0.2049485656	-2.3626421784
H	-5.2419662288	-0.4659464160	-3.2193445318
C	-3.8614352562	0.3011419142	-1.7843783527
H	-3.0246354983	-0.2775066235	-2.1828232622
C	-7.6024895229	0.7455413879	-2.5798669550
C	-8.7016987150	1.5834983966	-1.9178784840
H	-8.4805683989	2.6628862906	-1.9638730399
H	-8.8483213696	1.3065996778	-0.8604754344

H	-9.6596152591	1.4198105452	-2.4392350219
C	-7.4949527429	1.1738182280	-4.0544392504
H	-6.7382933849	0.5856981298	-4.5988832180
H	-7.2150175314	2.2374951203	-4.1362210564
H	-8.4621471363	1.0331301476	-4.5677388245
C	-8.0167266511	-0.7351112930	-2.5039701250
H	-8.1042719281	-1.0662991717	-1.4555672478
H	-7.2848950134	-1.3933774185	-3.0000335084
H	-8.9927651477	-0.8888252549	-2.9963130218
C	-1.3058004017	0.8566441746	-0.0627984359
C	-1.1368848054	-0.4858890070	0.3604856610
N	-2.2502504201	-1.2602359807	0.7467381311
C	-3.0539857397	-1.1143368724	1.8812845547
C	-4.0878659277	-2.0816196325	1.8403529473
C	-5.0147184982	-2.1460586664	2.8835310405
H	-5.8099523913	-2.8971823050	2.8430523834
C	-4.9301819762	-1.2672163717	3.9723805660
C	-3.8748226686	-0.3327864680	3.9874805322
H	-3.7778602557	0.3616483092	4.8246574062
C	-2.9305595826	-0.2449942764	2.9625647633
H	-2.1319000526	0.4984521260	3.0157085330
C	-5.9715053886	-1.3534356726	5.0977132422
C	-7.3738096238	-1.1049824267	4.5143090168
H	-7.6329723074	-1.8461942235	3.7408241199
H	-7.4367564804	-0.1047203423	4.0539635154
H	-8.1391741096	-1.1673482560	5.3075651273
C	-5.9207685921	-2.7535632386	5.7347812891
H	-6.1427888651	-3.5469077079	5.0023885303
H	-6.6606794701	-2.8365620163	6.5501929402
H	-4.9227351619	-2.9587906960	6.1569646080
C	-5.7235757271	-0.3185864201	6.2017278512
H	-5.7651781999	0.7143292119	5.8166146848
H	-4.7460983626	-0.4654040024	6.6908058067
H	-6.5001434051	-0.4138662653	6.9788697257
C	-3.8980455686	-2.8497413577	0.6256113004
C	-2.7511889681	-2.3211663260	-0.0119368922
C	-2.2989954314	-2.8424995823	-1.2223608016
H	-1.4172443251	-2.4360141105	-1.7265745309
C	-3.0091920343	-3.9030526461	-1.7877765579
H	-2.6472552804	-4.3095945934	-2.7345138646
C	-4.1592900095	-4.4541920360	-1.1863101277
C	-4.5905093390	-3.9079997345	0.0312728399
H	-5.4814998793	-4.3054241879	0.5281227207
C	-4.9476914307	-5.6069033880	-1.8261056710
C	-5.0319068371	-6.7869999494	-0.8420753174
H	-5.5463031509	-6.5108961044	0.0929579462
H	-4.0261121927	-7.1529083673	-0.5759429776
H	-5.5924342709	-7.6236212624	-1.2939604666
C	-6.3689897836	-5.1218036767	-2.1647554917
H	-6.9074424422	-4.7763902535	-1.2670582980
H	-6.9598227099	-5.9372558035	-2.6175592666

H	-6.3397714022	-4.2829956552	-2.8806677355
C	-4.2920444508	-6.1117673162	-3.1167563583
H	-3.2700864542	-6.4861652011	-2.9386060619
H	-4.2431107037	-5.3270266011	-3.8900099312
H	-4.8821781449	-6.9457372520	-3.5321781521
C	0.1372400941	-1.0886285363	0.3553894182
C	0.3199505388	-2.4400442422	0.9416635215
C	0.0361966165	-2.6832494585	2.2949003105
H	-0.3280253753	-1.8739926752	2.9429070979
N	0.1984582702	-3.8761689509	2.8575908483
C	0.6508747553	-4.8474772041	2.0657949597
H	0.7891073577	-5.8351303247	2.5272267299
N	0.9530477733	-4.7442502995	0.7738124591
C	0.7857431398	-3.5471311503	0.2174988183
H	1.0270708690	-3.4609915173	-0.8495062353
C	1.2413674534	-0.3559926107	-0.1245894595
N	2.5313755270	-0.9321375415	-0.0807159182
C	3.3650817177	-0.9386640174	1.0375379784
C	4.5582313658	-1.6269239413	0.7213350691
C	5.5630088138	-1.7606875483	1.6892185283
H	6.4813901805	-2.2907577658	1.4259647633
C	5.3883202846	-1.2261562069	2.9680116777
C	4.1695674039	-0.5651780977	3.2524428480
H	4.0097771457	-0.1483803106	4.2517690084
C	3.1545743637	-0.4123011258	2.3132829282
H	2.2273318899	0.1060216750	2.5716652205
C	6.4630044804	-1.3202885374	4.0592760263
C	7.7093062767	-2.0713508713	3.5768895750
H	8.1786007527	-1.5708462237	2.7136710180
H	7.4778506804	-3.1106146746	3.2897780044
H	8.4576903824	-2.1085038364	4.3859423986
C	6.8919832513	0.0995374531	4.4708863645
H	6.0471954142	0.6884119197	4.8641041987
H	7.3083560484	0.6482634362	3.6095145273
H	7.6657561584	0.0569989941	5.2573032713
C	5.8950223895	-2.0609907673	5.2824031766
H	5.5954083753	-3.0887114111	5.0169348520
H	5.0097684062	-1.5510831485	5.6958193770
H	6.6516172586	-2.1204292841	6.0841800051
C	4.4263101541	-2.0810892191	-0.6458202023
C	3.1624491693	-1.6417950363	-1.1020886171
C	2.7049172526	-1.9795312191	-2.3779357354
H	1.7240343500	-1.6569671288	-2.7354768214
C	3.5406639281	-2.7408869414	-3.1896132463
H	3.1831153259	-3.0063346659	-4.1892820497
C	4.8210061952	-3.1798651729	-2.7769516819
C	5.2483470745	-2.8385735079	-1.4914868540
H	6.2223953827	-3.1681088478	-1.1227125245
C	5.6749569886	-4.0167491583	-3.7391328815
C	5.9237369999	-3.2206442695	-5.0323553152
H	6.4584028848	-2.2797358449	-4.8188602198

H	4.9821428359	-2.9623989939	-5.5440410145
H	6.5352277101	-3.8101660971	-5.7376177408
C	7.0346918310	-4.3804769343	-3.1324244674
H	7.6258203777	-3.4825734758	-2.8867660074
H	7.6186399049	-4.9760295311	-3.8539625167
H	6.9267330583	-4.9847050275	-2.2164351376
C	4.9328011816	-5.3228733790	-4.0757754039
H	3.9618264044	-5.1293830245	-4.5603931219
H	4.7413335602	-5.9124104890	-3.1636825209
H	5.5333344833	-5.9415191888	-4.7653285215
C	1.0774464161	0.9702882302	-0.5805052987
C	-0.2012335414	1.5700574539	-0.5737733809
C	-0.3404729382	2.9889471696	-1.1246457362
F	0.1006630018	3.8896618614	-0.2312105653
F	0.3730902790	3.1456391420	-2.2439559004
F	-1.5943246336	3.3149101135	-1.4355055518
N	2.2205183595	1.6915014475	-0.9951944052
C	2.8047380245	1.6447090958	-2.2611055103
C	4.0427430870	2.3293508646	-2.2230340982
C	4.8182001242	2.4186677207	-3.3823881277
H	5.7755256710	2.9483793618	-3.3411388242
C	4.3806772330	1.8438587716	-4.5830618917
C	3.1251435543	1.2006643165	-4.5885044970
H	2.7443260869	0.7652693943	-5.5151581286
C	2.3267344303	1.0978474016	-3.4489179934
H	1.3458833955	0.6175367062	-3.5012465528
C	5.2580391463	1.9508874716	-5.8395183737
C	5.3921620631	3.4312282548	-6.2397097634
H	5.8466683524	4.0322870271	-5.4350284800
H	4.4048890814	3.8680298730	-6.4657870167
H	6.0266192073	3.5366347506	-7.1374617874
C	6.6517088311	1.3700488203	-5.5430769157
H	7.1567676077	1.9086733903	-4.7253039950
H	7.2987342986	1.4381112939	-6.4352217769
H	6.5815976604	0.3085542442	-5.2512166565
C	4.6675944527	1.1838664796	-7.0283087657
H	3.6890629605	1.5878755442	-7.3370734506
H	4.5440338384	0.1113312311	-6.8023819183
H	5.3442688904	1.2658770878	-7.8953891605
C	4.1990753019	2.8346911691	-0.8743462262
C	3.0536535680	2.4311725624	-0.1527299194
C	2.8759688759	2.8074717163	1.1792259414
H	1.9772286151	2.5300691907	1.7367807754
C	3.8710206456	3.5756791449	1.7762892485
H	3.7271613539	3.8764086050	2.8189089243
C	5.0411454122	3.9834247676	1.0944813309
C	5.1867530750	3.6015791550	-0.2421520054
H	6.0680088713	3.8994526924	-0.8150182508
C	6.0787117870	4.8459380723	1.8264491275
C	6.5298465455	4.1401974869	3.1175610758
H	6.9886679356	3.1624866363	2.8944961335

H	5.6892333172	3.9641547389	3.8080562947
H	7.2744974891	4.7554907055	3.6518022106
C	7.3213808208	5.1023188437	0.9670505769
H	7.8215366012	4.1614191570	0.6825240974
H	8.0488977662	5.7080497781	1.5330260169
H	7.0798110236	5.6571682707	0.0452306449
C	5.4454762435	6.2038653603	2.1811328510
H	4.5651771875	6.0842592944	2.8338174933
H	5.1184378013	6.7347366870	1.2714518217
H	6.1722026316	6.8448832914	2.7103737170

Optimized Molecular structure data for **CN2** using Terachem 1.93 software
E=-4.1478748059861719e+03a.u

H	-5.0841319599	-0.8239267975	7.5979374637
C	-4.4374929306	-0.8386135874	6.7043494635
H	-4.1709733206	0.2062464686	6.4723997340
H	-3.5134090442	-1.3779522271	6.9728510607
C	-5.1778092241	-1.5102927981	5.5426044432
C	-6.4620775556	-0.7067796860	5.2687232785
H	-7.0659056984	-1.1581449484	4.4642292728
H	-6.2207008103	0.3255281067	4.9633951908
H	-7.0921104981	-0.6593351914	6.1745565217
C	-5.5473163569	-2.9408814524	5.9731116556
H	-6.1263893472	-3.4671627182	5.1968122842
H	-6.1598386431	-2.9238202556	6.8916781475
H	-4.6417620736	-3.5369837955	6.1760282379
C	-4.3311538860	-1.5583615407	4.2622053158
C	-3.0388277825	-0.9977328075	4.1860580580
H	-2.6013475399	-0.5243808712	5.0677635089
C	-2.2786218708	-1.0153534785	3.0161250460
H	-1.2788840078	-0.5740851530	2.9979788044
C	-2.8330195742	-1.6074005956	1.8848919159
N	-2.2985250712	-1.7628659867	0.6042679241
C	-3.2260763693	-2.4560636059	-0.1765693523
C	-4.3652774779	-2.7446259182	0.6077747458
C	-4.1178554541	-2.1986498384	1.9256878130
C	-4.8532106963	-2.1667784162	3.1129468693
H	-5.8489111654	-2.6214351287	3.1343678872
C	-5.4339500845	-3.4606393811	0.0526886632
H	-6.3034788321	-3.6765690549	0.6778595422
C	-5.3796502554	-3.9012108316	-1.2716816006
C	-4.2173629744	-3.6037199848	-2.0218387888
H	-4.1479908538	-3.9518375394	-3.0572173190
C	-3.1414696777	-2.8927866446	-1.4998621582
H	-2.2530535786	-2.7021936146	-2.1079336279
C	-6.5100976486	-4.7108157501	-1.9214556758
C	-7.7124127599	-4.8737111457	-0.9854120358
H	-8.1393862169	-3.8991787244	-0.6948710379
H	-7.4487100646	-5.4222375202	-0.0657001559

H	-8.5058211298	-5.4464388755	-1.4942749153
C	-6.9935592646	-4.0011375696	-3.1989565109
H	-7.3697069281	-2.9900772035	-2.9697894605
H	-7.8106789989	-4.5739076164	-3.6714170502
H	-6.1871449157	-3.8986388305	-3.9433505561
C	-5.9856227022	-6.1129849070	-2.2814078429
H	-5.6379352960	-6.6452330241	-1.3801015903
H	-5.1411112150	-6.0637906148	-2.9884151979
H	-6.7815974779	-6.7173920645	-2.7511638109
C	-1.1270677786	-1.1067524632	0.1567829880
C	-1.2140566594	0.2438145947	-0.2406311281
N	-2.4357504176	0.9467188710	-0.1403340342
C	-2.7293372953	1.8524607043	0.8810561584
C	-4.0065569023	2.4162587741	0.6458186661
C	-4.5276302979	3.3456626126	1.5502518551
H	-5.5183749637	3.7717201616	1.3615986325
C	-3.7952780459	3.7338282236	2.6799489161
C	-2.5165271686	3.1674841037	2.8661464185
H	-1.9155408021	3.4590215392	3.7302368847
C	-1.9689838498	2.2347163714	1.9841975474
H	-0.9654668842	1.8354064067	2.1529230222
C	-4.3940359365	4.7580946836	3.6559199749
C	-4.6227345950	6.0903891450	2.9194705281
H	-5.3130095923	5.9725145639	2.0682148381
H	-3.6725561602	6.4907695044	2.5276861873
H	-5.0562366716	6.8425303455	3.6018376105
C	-5.7377192081	4.2314862850	4.1906885229
H	-6.4633156924	4.0600405734	3.3792518215
H	-6.1874712876	4.9548366515	4.8933010788
H	-5.6010526488	3.2753921785	4.7238494773
C	-3.4759078377	5.0230500129	4.8541156134
H	-2.5034434517	5.4416959264	4.5452083903
H	-3.2862405576	4.1045313614	5.4342128901
H	-3.9481001888	5.7537898823	5.5323032849
C	-4.4857905653	1.8517408286	-0.5975542208
C	-3.4833521988	0.9700441646	-1.0613103771
C	-3.6161286722	0.3232805316	-2.2906637502
H	-2.8301953762	-0.3277411342	-2.6780768557
C	-4.7783505294	0.5456909060	-3.0217532875
H	-4.8821052482	0.0333494745	-3.9834066860
C	-5.8154017465	1.4010091943	-2.5789595580
C	-5.6465653103	2.0542573910	-1.3561241629
H	-6.4098708730	2.7379927761	-0.9777961143
C	-7.0619272756	1.5924967020	-3.4535119628
C	-7.7621705235	0.2369749651	-3.6574405269
H	-8.0851885927	-0.1880915229	-2.6921723071
H	-7.0988620658	-0.4986196914	-4.1409383421
H	-8.6544841609	0.3557721907	-4.2967504496
C	-8.0670405191	2.5596798785	-2.8184169165
H	-8.4257966944	2.1939166768	-1.8420200881

H	-8.9456427038	2.6708651371	-3.4757369970
H	-7.6335138980	3.5630585268	-2.6722261985
C	-6.6477703947	2.1632287302	-4.8220486064
H	-5.9467818333	1.4964451962	-5.3501338272
H	-6.1552163587	3.1434767181	-4.7069891970
H	-7.5327145831	2.2979024454	-5.4684515582
C	-0.0572296734	0.9382667098	-0.6302178910
C	-0.1578822297	2.3787728672	-1.0146342332
N	0.5133537628	3.2443303927	-0.2545556957
C	0.4088156752	4.5287308947	-0.5902436213
H	0.9566609956	5.2477145144	0.0324364929
C	-0.3589385161	4.9500567163	-1.6761262263
H	-0.4425385938	6.0058692251	-1.9464645229
C	-1.0134489280	3.9527392910	-2.3984090335
N	-0.9158109249	2.6644232844	-2.0730260781
H	-1.6366675353	4.1959833668	-3.2686837975
C	1.1945762347	0.3079521810	-0.5869976934
N	2.3377513557	1.0220363923	-1.0002097224
C	3.3820958759	1.4718551538	-0.1898639625
C	4.3482191976	2.1172816889	-0.9972867597
C	5.4981718174	2.6514960011	-0.4108915472
H	6.2401862527	3.1434362860	-1.0480925740
C	5.7017336392	2.5630627859	0.9723230493
C	4.7008418974	1.9418070972	1.7479193322
H	4.8211250081	1.8686471808	2.8310126376
C	3.5411741482	1.4009790821	1.1914086786
H	2.7877503535	0.9383928387	1.8319093594
C	6.9815603838	3.1474375554	1.5885613343
C	8.2103027515	2.4827418427	0.9424921911
H	8.2405029876	2.6474852869	-0.1468396285
H	8.2041118405	1.3935342372	1.1155987658
H	9.1421662366	2.8937769566	1.3688438877
C	7.0257010255	4.6645650840	1.3308370124
H	6.1576517195	5.1670069466	1.7896147553
H	7.0148474554	4.8940869293	0.2528049467
H	7.9427007016	5.1053155302	1.7598843552
C	7.0563908989	2.9134949668	3.1019644261
H	7.0439240321	1.8388056244	3.3497520668
H	6.2241235680	3.4009085892	3.6365812299
H	7.9952334988	3.3365232063	3.4971674845
C	3.8502457172	2.0789423118	-2.3567861381
C	2.6012643658	1.4137683134	-2.3151560990
C	1.8559878769	1.2197201505	-3.4772841823
H	0.8780963431	0.7298327149	-3.4585691042
C	2.3849179045	1.6891173105	-4.6821093805
H	1.7942182212	1.5317210737	-5.5876289238
C	3.6290793527	2.3478703814	-4.7642873655
C	4.3497846812	2.5380134134	-3.5771415722
H	5.3185061732	3.0479318792	-3.5953543128
C	4.2046520422	2.8675096726	-6.0897512110

C	4.3379764748	4.3995927894	-6.0184903501
H	5.0025348889	4.7138656836	-5.1969913749
H	3.3552697366	4.8726968587	-5.8541864570
H	4.7571312995	4.7976867794	-6.9592264800
C	5.5916621556	2.2450620177	-6.3297710166
H	6.3006711474	2.4993840045	-5.5254381650
H	6.0207722928	2.6093232016	-7.2795045193
H	5.5273590030	1.1451192966	-6.3825473218
C	3.3130811982	2.5194313951	-7.2867780836
H	2.3110275232	2.9719113301	-7.2009889816
H	3.1896753379	1.4296519295	-7.4035529940
H	3.7697076669	2.9032586109	-8.2144414058
C	1.3009086374	-1.0365923276	-0.1496750472
C	0.1296224012	-1.7534305012	0.1785859256
C	0.1624045278	-3.2259549960	0.5812645982
F	-0.7924044689	-3.9133099750	-0.0553282490
F	1.3115133646	-3.8368924785	0.2949719447
F	-0.0477911318	-3.3666056762	1.8997985866
N	2.5824950107	-1.6070675969	-0.0252890845
C	3.2156145389	-1.9042657404	1.1836018914
C	4.5111610817	-2.4044410635	0.9129512436
C	5.3528796915	-2.7534791027	1.9713512488
H	6.3531954907	-3.1379967085	1.7474855722
C	4.9284957126	-2.6174389488	3.2999859328
C	3.6287393814	-2.1207472066	3.5299641393
H	3.2653498228	-2.0081358344	4.5539475513
C	2.7636398907	-1.7629948862	2.4940971654
H	1.7609969557	-1.3908950266	2.7229393731
C	5.8753885015	-3.0161113788	4.4419700636
C	6.2272805810	-4.5090901943	4.3127853786
H	6.7184928024	-4.7310713605	3.3513721403
H	5.3214133930	-5.1350215973	4.3783209362
H	6.9158208901	-4.8166703965	5.1193949354
C	7.1650478969	-2.1798225867	4.3542856885
H	7.6844532346	-2.3342634833	3.3947394791
H	7.8635057650	-2.4588646943	5.1624871089
H	6.9451393594	-1.1026629563	4.4469715060
C	5.2480372587	-2.7912460227	5.8221475852
H	4.3337854241	-3.3925625874	5.9586574230
H	4.9929729255	-1.7316132272	5.9914771023
H	5.9616394130	-3.0906929183	6.6080795015
C	4.6561090067	-2.4311654471	-0.5273095810
C	3.4469342752	-1.9420779221	-1.0688518670
C	3.2490608232	-1.8923804399	-2.4494537743
H	2.3107982035	-1.5393410791	-2.8841541808
C	4.2934633493	-2.3074146208	-3.2707168280
H	4.1410527994	-2.2605036960	-4.3534916440
C	5.5284004226	-2.7776448829	-2.7666802145
C	5.6886991980	-2.8412004019	-1.3800142435
H	6.6170950541	-3.2137597825	-0.9411394759

C	6.6327917381	-3.1940395228	-3.7470345156
C	7.0303069015	-1.9830198145	-4.6109882129
H	7.4060167647	-1.1576832820	-3.9832893334
H	6.1772748486	-1.5966162965	-5.1929079123
H	7.8249616763	-2.2625009622	-5.3247641467
C	7.8862125638	-3.6948806627	-3.0211034487
H	8.3215707625	-2.9181126894	-2.3705424724
H	8.6560006072	-3.9781873531	-3.7583616108
H	7.6744080948	-4.5840719990	-2.4041208821
C	6.1206318993	-4.3252747995	-4.6562878396
H	5.2408276022	-4.0147297228	-5.2432079247
H	5.8330473424	-5.2089723516	-4.0622210166
H	6.9053760495	-4.6322482673	-5.3697271635

Optimized Molecular structure data for **CN3** using Terachem 1.93 software

E=-6.4581732351791143e+03a.u

H	-4.3769685166	6.4634899256	5.4437642333
C	-3.8421232084	5.7750621777	4.7683753047
H	-3.0145731131	6.3388776541	4.3066037917
H	-3.4094417450	4.9713538882	5.3873044284
C	-4.8137731883	5.2245467184	3.7191061007
C	-5.3929383550	6.4100053554	2.9260107504
H	-6.1216836472	6.0755444838	2.1697088279
H	-4.5935585304	6.9642135650	2.4058714945
H	-5.9101111461	7.1102841673	3.6049292630
C	-5.9549402158	4.4977365097	4.4534348268
H	-6.7219893162	4.1299686785	3.7529264290
H	-6.4516016824	5.1799095949	5.1653399616
H	-5.5719170387	3.6322239427	5.0208407509
C	-4.1307545837	4.2435961892	2.7537807373
C	-2.7594794897	3.9206613907	2.8437717823
H	-2.1417464572	4.3925282645	3.6114001495
C	-2.1405443137	3.0083012316	1.9857783074
H	-1.0760235304	2.7832569290	2.0967874910
C	-2.9209426848	2.4008828534	1.0035068971
N	-2.5813071009	1.4353845665	0.0512136624
C	-3.7165961123	1.1455355666	-0.7068662795
C	-4.8035916105	1.9050257402	-0.2202021778
C	-4.2972696502	2.7060977986	0.8755470058
C	-4.8873520752	3.6230530206	1.7496460851
H	-5.9545335219	3.8445893562	1.6447623043
C	-6.0651693111	1.7845906666	-0.8192708644
H	-6.8948731998	2.3746801887	-0.4224608393
C	-6.2510518824	0.9246087175	-1.9056779598
C	-5.1300276164	0.2044284624	-2.3838137429
H	-5.2488764646	-0.4609411910	-3.2449634341
C	-3.8659344317	0.3011521845	-1.8082577736
H	-3.0263246653	-0.2678448711	-2.2143280977
C	-7.6100482633	0.7427166945	-2.5939745397

C	-8.7080457907	1.5858560610	-1.9365156985
H	-8.4811436655	2.6640354098	-1.9816736393
H	-8.8606929920	1.3098594515	-0.8796094039
H	-9.6628311906	1.4250922286	-2.4644307794
C	-7.4984173705	1.1647270951	-4.0701903441
H	-6.7481757249	0.5657054689	-4.6117643613
H	-7.2107990835	2.2262567259	-4.1553702087
H	-8.4680687527	1.0303427321	-4.5800656544
C	-8.0279260395	-0.7369008731	-2.5159811875
H	-8.1323482936	-1.0623593481	-1.4671593076
H	-7.2907416039	-1.3978378575	-3.0004898153
H	-8.9974940269	-0.8879758944	-3.0212913807
C	-1.3085967335	0.8482065849	-0.0816522562
C	-1.1432864835	-0.5022429805	0.3216419609
N	-2.2515606602	-1.2800812228	0.6997572281
C	-3.0342027678	-1.1455804805	1.8499905213
C	-4.0848146438	-2.0943965830	1.8037561100
C	-5.0108188634	-2.1523870425	2.8486222930
H	-5.8221175344	-2.8854212513	2.7996594002
C	-4.9050078337	-1.2858382315	3.9459040958
C	-3.8243405043	-0.3795108841	3.9728335109
H	-3.7032439048	0.2941735894	4.8238525143
C	-2.8820671801	-0.2984873624	2.9449522329
H	-2.0553110890	0.4123084045	3.0107566372
C	-5.9495661721	-1.3575674861	5.0690058632
C	-7.3495072413	-1.1025182759	4.4822362486
H	-7.6156791543	-1.8482057995	3.7155788035
H	-7.4048139654	-0.1041059712	4.0164295006
H	-8.1132399669	-1.1522716101	5.2776926606
C	-5.9101074157	-2.7549587349	5.7130367810
H	-6.1212722902	-3.5504891462	4.9796124979
H	-6.6626862591	-2.8317571150	6.5171630410
H	-4.9188337243	-2.9563016488	6.1531474623
C	-5.6969663408	-0.3170921769	6.1664671517
H	-5.7228723143	0.7124029009	5.7707653004
H	-4.7243063011	-0.4711118984	6.6626093868
H	-6.4809098521	-0.3988563872	6.9375433466
C	-3.9136488056	-2.8535148019	0.5801823803
C	-2.7700616036	-2.3285873608	-0.0660204922
C	-2.3271466594	-2.8500745317	-1.2796404985
H	-1.4417308539	-2.4542546548	-1.7861192328
C	-3.0444727545	-3.9102112835	-1.8374172513
H	-2.6885102533	-4.3193933179	-2.7852613327
C	-4.1902843704	-4.4617879598	-1.2247399067
C	-4.6145480897	-3.9117974233	-0.0065592223
H	-5.4998763488	-4.3084197651	0.5007689393
C	-4.9764213428	-5.6248575299	-1.8488845448
C	-5.0375080710	-6.8009443867	-0.8578149344
H	-5.5436357860	-6.5239001209	0.0814583538
H	-4.0250294854	-7.1577913406	-0.6041514785

H	-5.5959764042	-7.6437187041	-1.3004978130
C	-6.4071056669	-5.1558982819	-2.1696359096
H	-6.9413019756	-4.8293275098	-1.2622449478
H	-6.9884355201	-5.9764583383	-2.6250295024
H	-6.3954028150	-4.3106621766	-2.8785988058
C	-4.3315233035	-6.1286406184	-3.1448764447
H	-3.3039209267	-6.4915249997	-2.9758318870
H	-4.2986543011	-5.3451252219	-3.9199222158
H	-4.9193614332	-6.9698894322	-3.5484907922
C	0.1447681828	-1.0644947826	0.3266112434
C	1.2601405578	-0.3254901753	-0.1040798899
N	2.5484080527	-0.8921887470	-0.0580858626
C	3.3943983319	-0.8789595700	1.0508322751
C	4.5764272891	-1.5881474111	0.7371156390
C	5.5839125133	-1.7232266600	1.7039387192
H	6.4942430883	-2.2686661372	1.4438491769
C	5.4149017569	-1.1805880551	2.9806632260
C	4.2051170334	-0.4996808958	3.2617315945
H	4.0474916192	-0.0806054437	4.2603972262
C	3.1920723809	-0.3381179945	2.3216740041
H	2.2669691624	0.1841827729	2.5786737046
C	6.4800512561	-1.2990632368	4.0791197137
C	7.7202898129	-2.0621576450	3.6001027251
H	8.2035535784	-1.5609686247	2.7450517005
H	7.4776393838	-3.0957273914	3.3017836105
H	8.4594995694	-2.1156316711	4.4164870461
C	6.9214220548	0.1102035831	4.5124375107
H	6.0800660965	0.6988248960	4.9135028948
H	7.3476719674	0.6676146605	3.6612241344
H	7.6917605812	0.0462849797	5.3005799402
C	5.8898197961	-2.0446132618	5.2890742176
H	5.5887979679	-3.0693242946	5.0126683284
H	5.0028490334	-1.5294635075	5.6922756981
H	6.6352772658	-2.1140316820	6.1003329522
C	4.4210259525	-2.0782492021	-0.6168081641
C	3.1456253401	-1.6573376419	-1.0589226132
C	2.6534053467	-2.0413036286	-2.3078998222
H	1.6519631164	-1.7538148784	-2.6377303968
C	3.4802500451	-2.8093056970	-3.1224096275
H	3.0987182883	-3.1097630189	-4.1032025326
C	4.7823954040	-3.2109473510	-2.7363005747
C	5.2346381317	-2.8426917037	-1.4661217189
H	6.2242534675	-3.1459549467	-1.1165891438
C	5.6410162730	-4.0198419187	-3.7184271776
C	5.8627672955	-3.1895487018	-4.9956635094
H	6.3859600534	-2.2456202184	-4.7659885043
H	4.9097244756	-2.9357021032	-5.4882394883
H	6.4754354105	-3.7532834799	-5.7205172379
C	7.0121970493	-4.3736152970	-3.1317533403
H	7.5938096040	-3.4726501472	-2.8750505203

H	7.5949336188	-4.9455453716	-3.8730557153
H	6.9230843166	-4.9971131771	-2.2265242053
C	4.9217736881	-5.3315189008	-4.0812325994
H	3.9462499685	-5.1456234320	-4.5593144275
H	4.7493866934	-5.9472738765	-3.1825179369
H	5.5337429417	-5.9192880858	-4.7872305286
C	1.0807313234	0.9990660857	-0.5551365129
C	-0.2050722054	1.5805999910	-0.5665374554
C	-0.3510221221	2.9982419063	-1.1129959379
F	0.0844368008	3.9025966865	-0.2184564589
F	0.3649468776	3.1634568192	-2.2309145777
F	-1.6054705375	3.3226639724	-1.4259825034
N	2.2204980737	1.7249305248	-0.9714166593
C	2.8091059690	1.6587401831	-2.2352486722
C	4.0444348746	2.3500176768	-2.2048675765
C	4.8201242542	2.4342198133	-3.3656906567
H	5.7729718137	2.9726809282	-3.3299694081
C	4.3849909560	1.8438444992	-4.5604209546
C	3.1377837346	1.1817681281	-4.5554895919
H	2.7628258812	0.7269319980	-5.4754285310
C	2.3400125963	1.0832299460	-3.4139424280
H	1.3699856540	0.5807562344	-3.4564709115
C	5.2573169048	1.9523060904	-5.8208498531
C	5.3941207415	3.4333538440	-6.2187831679
H	5.8593495041	4.0306719897	-5.4172433600
H	4.4071576794	3.8739958461	-6.4394666263
H	6.0227116384	3.5344778880	-7.1210425377
C	6.6526498853	1.3703996635	-5.5335464421
H	7.1634545239	1.9107409127	-4.7202699531
H	7.2906694127	1.4395427910	-6.4320250959
H	6.5853601296	0.3079208414	-5.2437652574
C	4.6587512465	1.1896386161	-7.0079512407
H	3.6734055840	1.5888246640	-7.3005352455
H	4.5456501223	0.1147345201	-6.7880616509
H	5.3257996737	1.2845942705	-7.8810362669
C	4.2009091673	2.8660774199	-0.8599101550
C	3.0545773768	2.4697807044	-0.1344796378
C	2.8791072150	2.8527474342	1.1966801955
H	1.9813810758	2.5780750296	1.7573316918
C	3.8806101746	3.6171035237	1.7895238721
H	3.7412232484	3.9220001688	2.8316168492
C	5.0535425739	4.0146750935	1.1040616549
C	5.1950825270	3.6298473128	-0.2323044730
H	6.0777160907	3.9188669271	-0.8079451038
C	6.1000524913	4.8687196145	1.8329504102
C	6.5546146406	4.1553231402	3.1185899891
H	7.0195821021	3.1819482374	2.8876320376
H	5.7146652988	3.9720537897	3.8078961313
H	7.2972023709	4.7720576611	3.6537074421
C	7.3401271243	5.1233609883	0.9691752464

H	7.8375064659	4.1816395505	0.6819133849
H	8.0691072448	5.7258775986	1.5366702668
H	7.0976809305	5.6801943955	0.0487997621
C	5.4726991424	6.2265512716	2.1979119608
H	4.5953580914	6.1043169227	2.8541552430
H	5.1457807795	6.7642050407	1.2919508135
H	6.2049555043	6.8604510792	2.7275905391
Br	0.4083125532	-2.7943307946	0.9933594292

Optimized Molecular structure data for **CN4** using Terachem 1.93 software
E=-4.2471577293650944e+03a.u

H	-3.4443482125	6.6510759810	5.3929669372
C	-3.0333474069	5.9088057803	4.6884258230
H	-2.2156322604	6.3987830327	4.1340322555
H	-2.6016922826	5.0884151730	5.2860611672
C	-4.1430816861	5.4117405652	3.7553110328
C	-4.7031832662	6.6222039543	2.9862102434
H	-5.5455581939	6.3368788119	2.3351714052
H	-3.9264424400	7.0775339729	2.3493768742
H	-5.0668586195	7.3923500502	3.6885600620
C	-5.2566633873	4.7985773577	4.6228312952
H	-6.1088912902	4.4515980808	4.0160466539
H	-5.6405432038	5.5438616254	5.3409727660
H	-4.8820107271	3.9335421576	5.1956652023
C	-3.6340820858	4.3678073885	2.7507335966
C	-2.2798477794	3.9790840070	2.6847875181
H	-1.5547706843	4.4219003340	3.3713834647
C	-1.8072133031	3.0453898306	1.7605915107
H	-0.7438870881	2.7888482573	1.7358154574
C	-2.7216847912	2.4775657965	0.8767065208
N	-2.5327453112	1.5168048608	-0.1211473962
C	-3.7520089448	1.3123378187	-0.7682699219
C	-4.7438648596	2.1062399606	-0.1511948032
C	-4.0863816898	2.8502225956	0.9038394101
C	-4.5283107350	3.7873350778	1.8413525934
H	-5.5867098561	4.0679616019	1.8521905452
C	-6.0613570501	2.0709186275	-0.6266868295
H	-6.8170028831	2.6874559836	-0.1340836779
C	-6.3986065714	1.2656913671	-1.7183928060
C	-5.3692775004	0.5137539266	-2.3311368305
H	-5.6052301604	-0.1084757823	-3.2000534277
C	-4.0530415413	0.5255348821	-1.8802415358
H	-3.2869992629	-0.0631842566	-2.3905498566
C	-7.8253172819	1.1796987782	-2.2774358065
C	-8.8095845386	2.0419105525	-1.4799074362
H	-8.5367919587	3.1101600210	-1.5060172834
H	-8.8683723395	1.7270056847	-0.4246292541
H	-9.8207226908	1.9492138549	-1.9103739058
C	-7.8332983833	1.6652895020	-3.7379601334

H	-7.4965945111	2.7132108162	-3.8066232603
H	-8.8511720383	1.6031278234	-4.1613742356
H	-7.1699595136	1.0583274469	-4.3756259401
C	-8.3112724197	-0.2799094346	-2.2209067847
H	-8.3185876061	-0.6554297517	-1.1838399937
H	-7.6665831830	-0.9507699094	-2.8120607102
H	-9.3360874592	-0.3599143979	-2.6234434064
C	-1.3193933402	0.8522334899	-0.3851583451
C	-1.2043740964	-0.5199154508	-0.0386014097
N	-2.3220465330	-1.1968329896	0.4995746308
C	-2.8413760671	-0.9931733324	1.7797937401
C	-3.9817996343	-1.8073679894	1.9563166170
C	-4.6842462175	-1.7709709305	3.1685754697
H	-5.5737803207	-2.3956504196	3.2819623412
C	-4.2530584035	-0.9467007646	4.2120542468
C	-3.0823296871	-0.1785279697	4.0111415632
H	-2.7183542914	0.4643411048	4.8186548017
C	-2.3674519821	-0.1885341361	2.8175603857
H	-1.4727018414	0.4284493024	2.7027766750
C	-4.9981095966	-0.8378625519	5.5488796665
C	-5.4153293012	0.6263847310	5.7770284882
H	-6.0668781397	0.9824128697	4.9615987430
H	-4.5436583142	1.2994444053	5.8228539127
H	-5.9663259017	0.7283849045	6.7282236756
C	-6.2629811161	-1.7030764136	5.5774022071
H	-6.9794718311	-1.4062502650	4.7933836508
H	-6.7686395269	-1.5874955914	6.5504874789
H	-6.0332513797	-2.7740947375	5.4493986379
C	-4.0772020311	-1.2932831504	6.6945363424
H	-3.1617518943	-0.6818458527	6.7551090210
H	-3.7706981533	-2.3443588868	6.5611464270
H	-4.5975876813	-1.2091418703	7.6646460088
C	-4.1490772615	-2.5574035607	0.7291631134
C	-3.1014786178	-2.1634512196	-0.1384309711
C	-2.9899761077	-2.7113346675	-1.4146151117
H	-2.1933218933	-2.4082705051	-2.0991037699
C	-3.9357524607	-3.6591109237	-1.8107345675
H	-3.8417135015	-4.0827960250	-2.8133325044
C	-4.9924241119	-4.0759167402	-0.9743355050
C	-5.0816229242	-3.5058932612	0.3026206396
H	-5.8884599286	-3.7985082743	0.9823102026
C	-6.0261654603	-5.1255241838	-1.4087649210
C	-5.9111060665	-6.3554144922	-0.4895496896
H	-6.0869285192	-6.0925872597	0.5664579237
H	-4.9063969527	-6.8049940853	-0.5585389970
H	-6.6510840837	-7.1246115890	-0.7724025415
C	-7.4417335167	-4.5324397261	-1.2988586857
H	-7.6867809804	-4.2370498859	-0.2657013189
H	-8.1961467413	-5.2717110203	-1.6200308410
H	-7.5468799703	-3.6375887230	-1.9353129969

C	-5.8144552546	-5.5863343507	-2.8552207409
H	-4.8330830868	-6.0696740456	-2.9946490296
H	-5.8917181106	-4.7486756769	-3.5685817672
H	-6.5866067269	-6.3256964871	-3.1260639147
C	0.0204220545	-1.2015829761	-0.1767749779
C	0.1078499747	-2.6438467750	0.2089017734
C	-0.0722221698	-2.9942233163	1.5586659047
H	-0.2701585811	-2.2121095721	2.2962405094
C	-0.0107994946	-4.3147728784	1.9804908611
H	-0.1508530370	-4.5692834612	3.0340903307
C	0.2327793110	-5.3244039839	1.0356308867
C	0.3010471906	-6.6983887509	1.4440010943
N	0.3558742096	-7.8113685542	1.7740857093
C	0.3937424476	-4.9851905253	-0.3132645452
H	0.5613257132	-5.7807550813	-1.0458999967
C	0.3297208301	-3.6561006942	-0.7500246643
C	0.4627858823	-3.3879389585	-2.2236102072
H	-0.2336147760	-4.0271696909	-2.7916977268
H	1.4819681444	-3.6136305027	-2.5784831045
H	0.2491863881	-2.3410360561	-2.4813769319
C	1.1627114573	-0.4821953853	-0.6043721697
N	2.4355362580	-1.0903920145	-0.5101532174
C	3.0705322868	-1.3609031787	0.7082231034
C	4.3444904433	-1.9203215710	0.4542580671
C	5.1696563470	-2.2849381386	1.5206119816
H	6.1512676487	-2.7201819054	1.3064356348
C	4.7511238675	-2.1024716591	2.8448573374
C	3.4855548350	-1.5186678504	3.0614557260
H	3.1364301590	-1.3485974766	4.0825168200
C	2.6413471915	-1.1410702957	2.0159022126
H	1.6726271287	-0.6820748924	2.2306325243
C	5.6682155081	-2.5415275712	3.9956103189
C	5.9471758370	-4.0505825508	3.8717937080
H	6.4371578413	-4.2995845631	2.9164352549
H	5.0112148725	-4.6314949326	3.9281109157
H	6.6109071150	-4.3900699290	4.6861612745
C	6.9968750686	-1.7685836810	3.9182650914
H	7.5175828968	-1.9494187211	2.9639057110
H	7.6728552905	-2.0806467570	4.7333460821
H	6.8294793343	-0.6818203824	4.0068267834
C	5.0394204090	-2.2858508189	5.3698866551
H	4.0939726331	-2.8394447064	5.4959048878
H	4.8393263108	-1.2147725778	5.5408394879
H	5.7284052826	-2.6236076088	6.1620653279
C	4.4945298636	-1.9791610119	-0.9830660135
C	3.3060006133	-1.4584592058	-1.5425296143
C	3.1380910921	-1.4186666046	-2.9277798091
H	2.2248063891	-1.0332579434	-3.3830130428
C	4.1790766463	-1.8770745247	-3.7298735461
H	4.0453763941	-1.8271186004	-4.8150620029

C	5.3898006082	-2.3853121940	-3.2039368139
C	5.5256656414	-2.4353992496	-1.8147993391
H	6.4356084527	-2.8289629449	-1.3555286409
C	6.4966785717	-2.8443661058	-4.1617140251
C	6.9397022788	-1.6554094794	-5.0337652045
H	7.3133332481	-0.8256501061	-4.4105601374
H	6.1097768167	-1.2660428185	-5.6455962749
H	7.7475376046	-1.9626263900	-5.7204462405
C	7.7255549956	-3.3700222074	-3.4113143235
H	8.1713135337	-2.5980090974	-2.7621820220
H	8.4983722393	-3.6792273261	-4.1347045336
H	7.4832836173	-4.2480011992	-2.7894106125
C	5.9692350049	-3.9712890040	-5.0676527482
H	5.1076443697	-3.6441822418	-5.6725525595
H	5.6504031241	-4.8428396763	-4.4714531171
H	6.7575378543	-4.3038692854	-5.7652935192
C	1.0469800900	0.8696160915	-1.0004886062
C	-0.2051709566	1.5249864181	-0.9248617786
C	-0.2831371608	2.9698765232	-1.4220975913
F	0.4531778730	3.7827268880	-0.6525659498
F	0.1887419930	3.0480200596	-2.6724293831
F	-1.5191379948	3.4612052096	-1.4581831785
N	2.2170432160	1.5923126050	-1.3444843388
C	2.7495065427	1.8579723764	-2.6110526404
C	3.9638164660	2.5643930102	-2.4584865796
C	4.6986261550	2.9505553375	-3.5881880583
H	5.6356420183	3.4937640604	-3.4434473027
C	4.2349076004	2.6548090194	-4.8713807537
C	3.0036453412	1.9656549282	-4.9862735420
H	2.6078966400	1.7418797161	-5.9822413683
C	2.2543287387	1.5666493159	-3.8844711402
H	1.2927850560	1.0645541336	-4.0221866720
C	4.9857848047	3.0784509010	-6.1416345591
C	4.1271798061	4.0951078683	-6.9156383638
H	3.9387298064	4.9932359463	-6.3040631042
H	3.1492263063	3.6724726923	-7.1989279979
H	4.6372195474	4.4123376613	-7.8423355688
C	6.3338402114	3.7341499915	-5.8216605404
H	6.9971908672	3.0549854968	-5.2603437530
H	6.2114893315	4.6596189548	-5.2350435479
H	6.8486249188	4.0037666325	-6.7589778198
C	5.2505499435	1.8490732055	-7.0276607166
H	4.3157941389	1.3564155069	-7.3411623523
H	5.8620505091	1.1025228936	-6.4937170281
H	5.7927748917	2.1421409853	-7.9436393661
C	4.1642321127	2.7604675528	-1.0391576665
C	3.0757404483	2.1418076302	-0.3881802706
C	2.9514472989	2.1817601161	1.0007293565
H	2.1122397432	1.7056784767	1.5140084007
C	3.9304378848	2.8584820188	1.7216980348

H	3.8333761922	2.8901390587	2.8113424604
C	5.0251053227	3.5097722978	1.1058317355
C	5.1299376814	3.4422120696	-0.2862478323
H	5.9548511376	3.9334040988	-0.8079987085
C	6.0413681310	4.2641509246	1.9746999523
C	6.8175714364	3.2563403439	2.8407427725
H	7.3784761246	2.5474428412	2.2088902970
H	6.1433240512	2.6667244187	3.4841754000
H	7.5382119984	3.7781300565	3.4948733081
C	7.0475796230	5.0508905000	1.1276226098
H	7.6643355667	4.3905819346	0.4956631708
H	7.7338690160	5.6094406204	1.7860707748
H	6.5451832546	5.7826974883	0.4729481890
C	5.3063900945	5.2659659583	2.8835586565
H	4.6184557543	4.7672789399	3.5853758046
H	4.7160398206	5.9811633288	2.2865130641
H	6.0297896387	5.8411251990	3.4874771556

Optimized Molecular structure data for **CN5** using Terachem 1.93 software
E=-4.1318628311502325e+03a.u

H	-3.7022135156	6.5130348392	5.4983952264
C	-3.2601183972	5.7830888239	4.7995214584
H	-2.3965915107	6.2691485823	4.3155409429
H	-2.8864080418	4.9371984245	5.4006177033
C	-4.3113614831	5.3402499270	3.7756511987
C	-4.8202215790	6.5939234638	3.0417293774
H	-5.6199911008	6.3542889675	2.3224098354
H	-4.0044111177	7.0848423627	2.4850899377
H	-5.2289435419	7.3224902725	3.7634297744
C	-5.4784010890	4.6821429418	4.5335424773
H	-6.2843421622	4.3704015906	3.8493510414
H	-5.9133416467	5.3836785197	5.2670388866
H	-5.1383928462	3.7854222737	5.0790763702
C	-3.7440704494	4.3395642463	2.7575015256
C	-2.4209944113	3.8573878769	2.8304495425
H	-1.7601398755	4.2049009345	3.6277067770
C	-1.9014590616	2.9412471560	1.9141565858
H	-0.8677866217	2.5969268366	2.0067273311
C	-2.7330047541	2.4936247388	0.8899268590
N	-2.4868157537	1.5640403457	-0.1233277081
C	-3.6400870834	1.4454674013	-0.9014472487
C	-4.6442130102	2.2898050037	-0.3691754186
C	-4.0668155007	2.9534974320	0.7831909697
C	-4.5576646194	3.8695336965	1.7168991372
H	-5.5930803573	4.2143479086	1.6282180246
C	-5.8905753552	2.3613253245	-0.9957087597
H	-6.6608381811	3.0174761724	-0.5774377812
C	-6.1539645186	1.6129844704	-2.1512211628
C	-5.1217461116	0.7988255790	-2.6616727986

H	-5.2908835387	0.2107992640	-3.5665236302
C	-3.8660210814	0.7070526298	-2.0600008080
H	-3.0920954270	0.0731854445	-2.4992608996
C	-7.5304017414	1.7181402484	-2.8239366631
C	-8.6233891607	1.2861372686	-1.8303458535
H	-8.6309161633	1.9140566816	-0.9241471551
H	-8.4736561053	0.2412964178	-1.5107616707
H	-9.6216151075	1.3628883215	-2.2960298002
C	-7.7751994798	3.1751325408	-3.2561721230
H	-7.7552689026	3.8668358954	-2.3980421732
H	-8.7608056677	3.2748558891	-3.7437561067
H	-7.0042700512	3.5079903358	-3.9712781895
C	-7.6400522125	0.8266366277	-4.0664031202
H	-7.5000574194	-0.2396109187	-3.8209154882
H	-6.9017711839	1.1018069296	-4.8379955508
H	-8.6421057072	0.9357154564	-4.5141468831
C	-1.2786141984	0.8593153964	-0.2736592929
C	-1.2598724320	-0.5330764821	-0.0159337463
N	-2.4556699034	-1.2314961146	0.2593860950
C	-3.2434731435	-1.1310664130	1.4065476499
C	-4.3466237396	-2.0081779480	1.2877071396
C	-5.2916467733	-2.0824497780	2.3205536273
H	-6.1421458006	-2.7589780750	2.2079441573
C	-5.1484856480	-1.3003481243	3.4698675402
C	-4.0148063360	-0.4594358171	3.5641390538
H	-3.8758609753	0.1561696893	4.4583345976
C	-3.0573901525	-0.3647237142	2.5587939229
H	-2.2019600890	0.3048430021	2.6761582459
C	-6.1719624208	-1.3097131117	4.6135016671
C	-6.7870616642	0.0951324542	4.7544050664
H	-7.3042388215	0.3939914457	3.8272376119
H	-6.0230705289	0.8604004610	4.9696378056
H	-7.5217745228	0.1133775176	5.5783219651
C	-7.3100735739	-2.3053310872	4.3626426331
H	-7.8755350011	-2.0586098126	3.4488096562
H	-8.0188305012	-2.2808372875	5.2071459187
H	-6.9393281405	-3.3398775207	4.2698870900
C	-5.4752175892	-1.6957041152	5.9304644488
H	-4.6699569233	-0.9895626027	6.1897868455
H	-5.0302519277	-2.7026682455	5.8625727015
H	-6.1983905417	-1.6963865851	6.7646290496
C	-4.2124561459	-2.6793097320	0.0118320248
C	-3.0357583034	-2.1785062200	-0.5880895836
C	-2.6255583521	-2.6170051764	-1.8488588195
H	-1.7061740930	-2.2481118993	-2.3110173385
C	-3.4120332034	-3.5674590555	-2.4910805857
H	-3.0923621311	-3.9131602309	-3.4790408890
C	-4.5893407873	-4.1057458992	-1.9191000757
C	-4.9776892175	-3.6449803843	-0.6578319335
H	-5.8805170944	-4.0298355651	-0.1770698652

C	-5.3959135454	-5.1464792862	-2.7077792521
C	-6.5395842883	-5.7417989695	-1.8785942453
H	-7.2812576515	-4.9797926683	-1.5870395620
H	-6.1677316378	-6.2291113247	-0.9617192631
H	-7.0700174924	-6.5056401378	-2.4714444153
C	-5.9975950170	-4.4710980885	-3.9537039283
H	-6.6639782477	-3.6405777947	-3.6659192934
H	-6.5853089552	-5.1945937614	-4.5458887118
H	-5.2131334790	-4.0578286839	-4.6093297037
C	-4.4784147787	-6.3016683790	-3.1468890119
H	-4.0159609492	-6.7949740023	-2.2754260255
H	-3.6665608321	-5.9587837496	-3.8082587848
H	-5.0558674157	-7.0609606186	-3.7024736924
C	-0.0474013193	-1.2426499853	-0.0208810455
C	-0.0330042499	-2.6828054654	0.3882469019
C	-0.2412822061	-3.0281640776	1.7306243180
H	-0.4180659925	-2.2538894870	2.4827060412
C	-0.2219798669	-4.3781303318	2.0759647406
H	-0.3819014641	-4.6841378385	3.1147041395
C	0.0031727496	-5.3244035869	1.0764183885
H	0.0250940642	-6.3946678912	1.3020853721
C	0.2002105733	-4.8733843871	-0.2302437367
N	0.1818385813	-3.5851775153	-0.5699310487
H	0.3812611957	-5.5867841820	-1.0446618237
C	1.1592159638	-0.5703462950	-0.2815340063
N	2.3845210804	-1.2682416104	-0.1738266226
C	3.2481104507	-1.1841705937	0.9177513923
C	4.4207322409	-1.9234851946	0.6425603310
C	5.4420274432	-1.9894379992	1.6006655033
H	6.3467465399	-2.5563141788	1.3676209165
C	5.3013030903	-1.3427129100	2.8316248203
C	4.0972609302	-0.6424443672	3.0816961136
H	3.9597137206	-0.1408185223	4.0448520160
C	3.0684700096	-0.5534699153	2.1496803393
H	2.1534464772	-0.0022802345	2.3807575671
C	6.3922203671	-1.3658557602	3.9109253209
C	7.6400111804	-2.1291410721	3.4520130472
H	8.0879312112	-1.6730824833	2.5535682021
H	7.4170511773	-3.1853947872	3.2274549660
H	8.4018121505	-2.1128199967	4.2493094668
C	6.8127664047	0.0768249700	4.2458815696
H	5.9702578510	0.6726445458	4.6329213168
H	7.2018946219	0.5916291505	3.3513595776
H	7.6040332760	0.0788764149	5.0159403389
C	5.8478489750	-2.0460620270	5.1794381945
H	5.5557927019	-3.0892833545	4.9726338528
H	4.9620709450	-1.5226630180	5.5752938724
H	6.6147697190	-2.0546692818	5.9737138928
C	4.2474419847	-2.4992558114	-0.6737846336
C	2.9798930451	-2.0830146248	-1.1401069383

C	2.5005553263	-2.5018949736	-2.3832633953
H	1.5046856261	-2.2180678832	-2.7305824065
C	3.3137836252	-3.3308723386	-3.1475960196
H	2.9365467178	-3.6660530581	-4.1191495787
C	4.5889396154	-3.7709042257	-2.7154776914
C	5.0422314160	-3.3397413591	-1.4669720661
H	6.0156519352	-3.6582685921	-1.0864158549
C	5.4047854229	-4.7095279419	-3.6154030778
C	5.6853230687	-4.0177895005	-4.9611079597
H	6.2586253881	-3.0879120686	-4.8125607616
H	4.7529270875	-3.7562160514	-5.4879488603
H	6.2692529111	-4.6803098461	-5.6238409577
C	6.7477596433	-5.0901681393	-2.9824558649
H	7.3817339272	-4.2055103706	-2.8058060304
H	7.3011301885	-5.7665866979	-3.6554081605
H	6.6129180386	-5.6151586163	-2.0221882309
C	4.6072999720	-6.0028422543	-3.8631186326
H	3.6454115063	-5.8017904485	-4.3624034041
H	4.3913384419	-6.5211352495	-2.9136974865
H	5.1796225258	-6.6932937197	-4.5073810124
C	1.1507410641	0.8169862881	-0.5524359824
C	-0.0754626009	1.5178597330	-0.6096349235
C	-0.0741239737	2.9499572405	-1.1443238495
F	0.3491089686	3.8415665053	-0.2375702923
F	0.7275846456	3.0483623139	-2.2122173868
F	-1.2829312980	3.3463994409	-1.5501324354
N	2.3755589923	1.4966547451	-0.7317073959
C	3.2343702869	1.3707155831	-1.8244600143
C	4.4254198756	2.0836281428	-1.5645294079
C	5.4403976044	2.1195567321	-2.5309095900
H	6.3584110152	2.6699195834	-2.3108372291
C	5.2742115323	1.4662278731	-3.7547908140
C	4.0528371485	0.7905184602	-3.9880510061
H	3.8960330814	0.2832757584	-4.9452732557
C	3.0282079059	0.7363641706	-3.0493532005
H	2.0957861548	0.2124923720	-3.2721484431
C	6.3518438986	1.4649812285	-4.8473483120
C	5.8012950957	2.1491769080	-6.1110905939
H	5.5267430262	3.1970689208	-5.9039601951
H	4.9028150281	1.6377829728	-6.4936156042
H	6.5579792930	2.1440735883	-6.9152040784
C	7.6178046490	2.2097749662	-4.4083503945
H	7.4146693322	3.2703374446	-4.1852064988
H	8.3683398077	2.1786537159	-5.2159238894
H	8.0709735423	1.7500311957	-3.5143968460
C	6.7434242012	0.0142060400	-5.1815862860
H	5.8857711654	-0.5680769004	-5.5556259643
H	7.1347121176	-0.5040275841	-4.2900180904
H	7.5248541014	-0.0040634137	-5.9614481588
C	4.2783431617	2.6769023401	-0.2523758808

C	3.0048706968	2.2972392145	0.2268988418
C	2.5588940122	2.7146766911	1.4824997840
H	1.5738966503	2.4299452221	1.8614322162
C	3.4014951482	3.5213369529	2.2394900987
H	3.0485877751	3.8550556334	3.2205151435
C	4.6785874447	3.9333617517	1.7895537855
C	5.1041146637	3.4930998835	0.5335724069
H	6.0827571666	3.7821434482	0.1429495732
C	5.5302030643	4.8472919027	2.6815129951
C	5.8150904662	4.1428763885	4.0197785732
H	6.3643079133	3.2004300975	3.8593392941
H	4.8855302698	3.9018383125	4.5612919102
H	6.4249277903	4.7890430384	4.6753058888
C	6.8718326306	5.1988509162	2.0294228715
H	7.4833979654	4.3008134445	1.8414811182
H	7.4500596517	5.8609310395	2.6955907140
H	6.7344981216	5.7291872350	1.0724738349
C	4.7667212309	6.1574894582	2.9469867142
H	3.8071088219	5.9759199190	3.4577926099
H	4.5496789435	6.6862529519	2.0037537564
H	5.3638763495	6.8299832092	3.5875985813

References

(1) Gudeika, D.; Volyniuk, D.; Grazulevicius, J. V.; Skuodis, E.; Yu, S. Y.; Liou, W. T.; Chen, L. Y.; Shiu, Y. J. Derivative of Oxygafluorene and Di-Tert-Butyl Carbazole as the Host with Very High Hole Mobility for High-Efficiency Blue Phosphorescent Organic Light-Emitting Diodes. *Dye. Pigment.* **2016**, *130*, 298–305. https://doi.org/10.1016/j.dyepig.2016.03.039.

(2) Rev. A.03 Citation | Gaussian.com https://gaussian.com/citation_a03/ (accessed Dec 11, 2020).

(3) Tomkeviciene, A.; Sutaite, J.; Volyniuk, D.; Kostiv, N.; Simkus, G.; Mimaite, V.; Grazulevicius, J. V. Aggregation-Induced Emission Enhancement in Charge-Transporting Derivatives of Carbazole and Tetra(Tri)Phenylethylene. *Dye. Pigment.* **2017**, *140*, 363–374. https://doi.org/10.1016/j.dyepig.2017.01.056.

(4) Kearns, D. R.; Calvin, M. Solid State Ionization Potentials of Some Aromatic Organic Compounds. *J. Chem. Phys.* **1961**, *34* (6), 2026–2030. https://doi.org/10.1063/1.1731815.

(5) Zhang, D.; Cai, M.; Zhang, Y.; Zhang, D.; Duan, L. Sterically Shielded Blue Thermally Activated Delayed Fluorescence Emitters with Improved Efficiency and Stability. *Mater. Horizons* **2016**, *3* (2), 145–151. https://doi.org/10.1039/c5mh00258c.

(6) Zou, S. J.; Xie, F. M.; Xie, M.; Li, Y. Q.; Cheng, T.; Zhang, X. H.; Lee, C. S.; Tang, J. X. High-Performance Nondoped Blue Delayed Fluorescence Organic Light-Emitting Diodes Featuring Low Driving Voltage and High Brightness. *Adv. Sci.* **2020**, *7* (3). https://doi.org/10.1002/ADVS.201902508.

(7) Mahmoudi, M.; Gudeika, D.; Volyniuk, D.; Leitonas, K.; Butkute, R.; Danyliv, I.; Grazulevicius, J. V. Tuning of Spin-Flip Efficiency of Blue Emitting Multicarbazolyl-Substituted Benzonitriles by Exploitation of the Different Additional Electron Accepting Moieties. *Chem. Eng. J.* **2021**, *423*, 130236. https://doi.org/10.1016/j.cej.2021.130236.

(8) Kreiza, G.; Banevičius, D.; Jovaišaitė, J.; Maleckaitė, K.; Gudeika, D.; Volyniuk, D.; Gražulevičius, J. V.; Juršėnas, S.; Kazlauskas, K. Suppression of Benzophenone-Induced Triplet Quenching for Enhanced TADF Performance. *J. Mater. Chem. C* **2019**, *7* (37), 11522–11531. https://doi.org/10.1039/c9tc02408e.

(9) Zhang, X.; Chi, Z.; Xu, B.; Chen, C.; Zhou, X.; Zhang, Y.; Liu, S.; Xu, J. End-Group Effects of Piezofluorochromic Aggregation-Induced Enhanced Emission Compounds Containing Distyrylanthracene. *J. Mater. Chem.* **2012**, *22* (35), 18505–18513. https://doi.org/10.1039/c2jm33140c.

(10) Ufimtsev, I. S.; Martinez, T. J. Quantum Chemistry on Graphical Processing Units. 3. Analytical Energy Gradients, Geometry Optimization, and First Principles Molecular Dynamics. *J. Chem. Theory Comput.* **2009**, *5* (10), 2619–2628. https://doi.org/10.1021/CT9003004/SUPPL_FILE/CT9003004_SI_001.PDF.
